# Supplementary material for: Chemically routed interpore molecular diffusion in metal-organic framework thin films
Source: Nat Commun. 2023 Apr 18;14:2212. doi: 10.1038/s41467-023-37739-8 (PMC10113335; doi:10.1038/s41467-023-37739-8)
Supplement: Supplementary file 1 — Supplementary Information [file 41467_2023_37739_MOESM1_ESM.docx]

Supplementary Information

**Chemically routed interpore molecular diffusion in metal-organic framework thin films**

Tanmoy Maity^1^,^±^ Pratibha Malik^1^,^±^ Sumit Bawari^1^, Soumya Ghosh^1^, Jagannath Mondal^1^, Ritesh Haldar^1✉^

^±^ Contributed equally

^✉^Email: riteshhaldar@tifrh.res.in

^1^Tata Institute of Fundamental Research Hyderabad, Gopanpally, Hyderabad 500046, Telangana, India

**General Information**

**Material**

Cu(OAc)_2_·H_2_O, 1,4-benzenedicarboxylic acid, 1,2-di(4-pyridyl)ethylene, diazabicyclo[2.2.2]octane and 4,4'-dithiodipyridine were purchased from Sigma-Aldrich. Anhydrous methanol and 1-butanol were also purchased from Sigma-Aldrich. All chemicals were used as received without further purification. QCM Sensors (5MHz) were purchased from Open qcm.


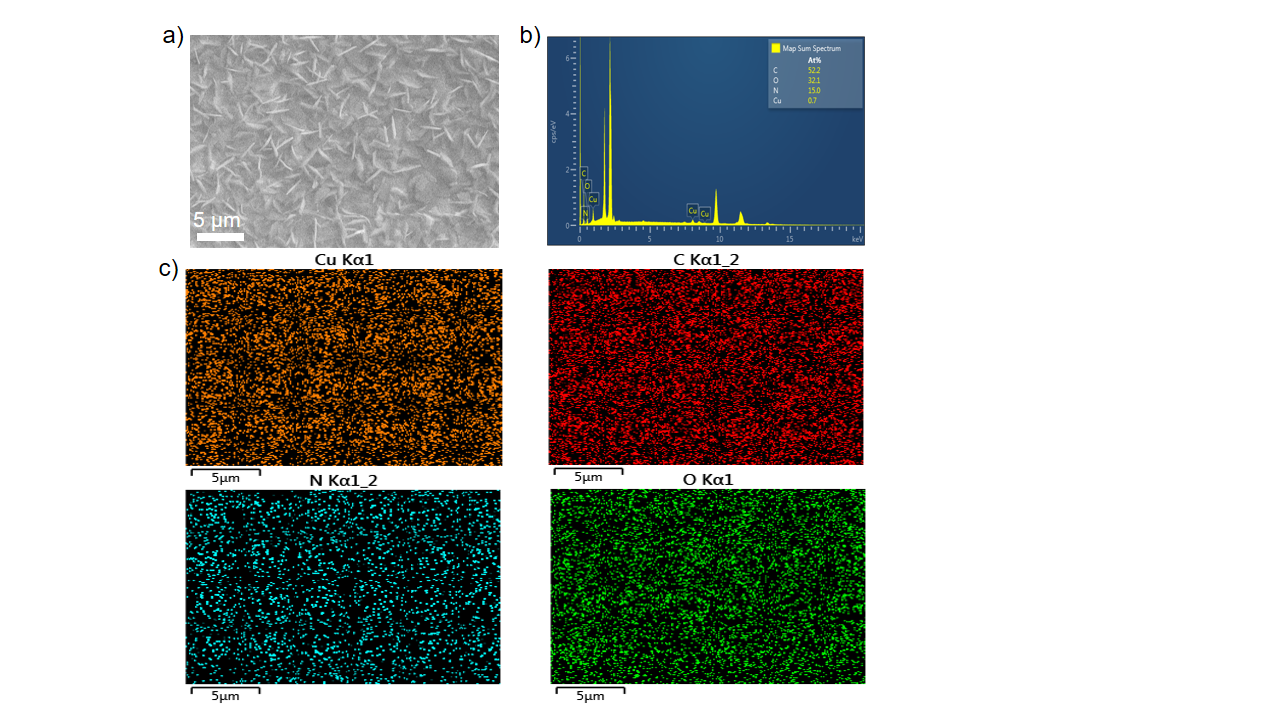


**Supplementary Figure 1**: a) SEM image, b) Elemental analyses and c) Elemental mapping for PL_C=C_.


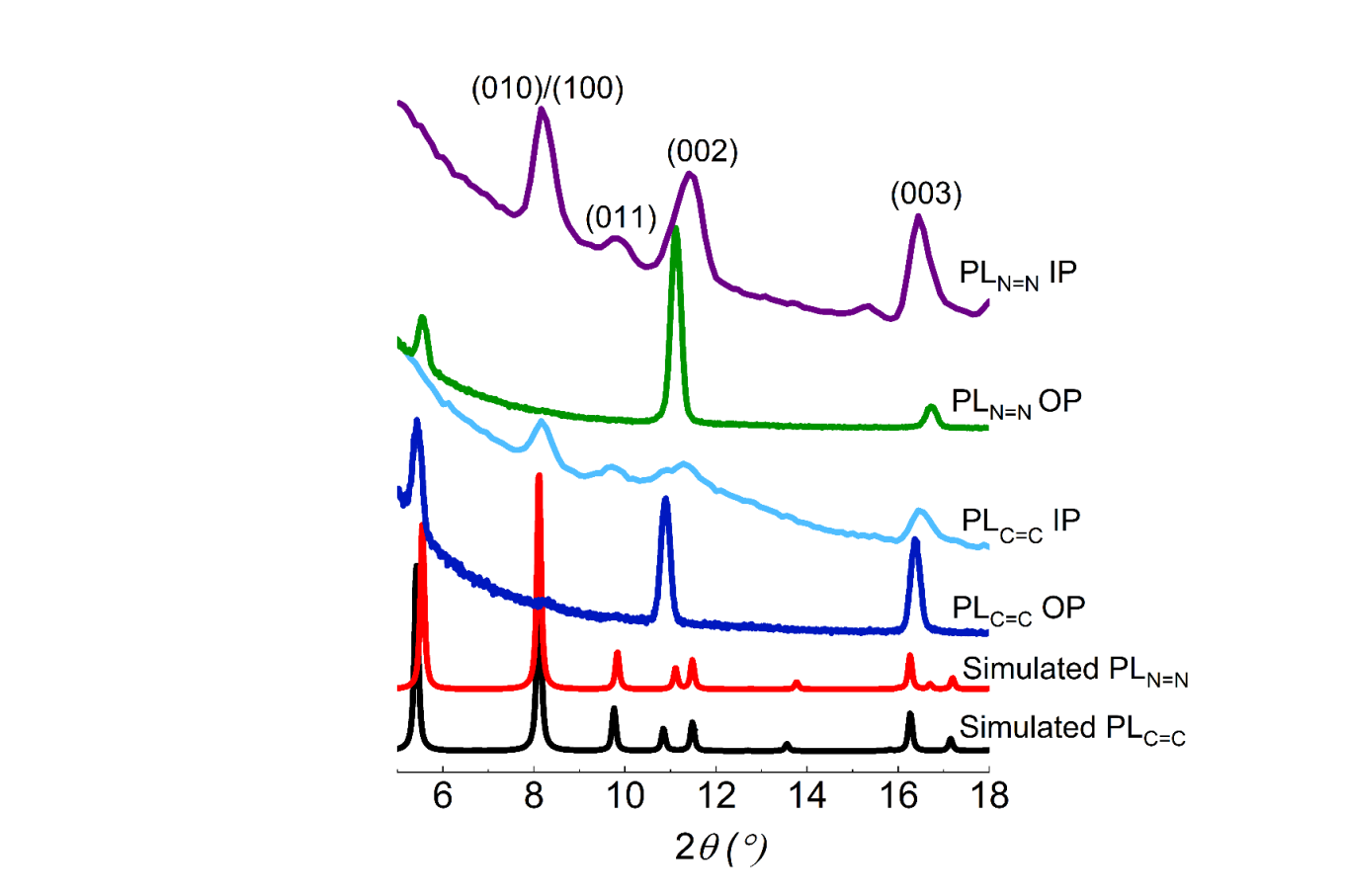


**Supplementary Figure 2**: Out and in-plane PXRD of PL_C=C_ and PL_N=N_ thin films


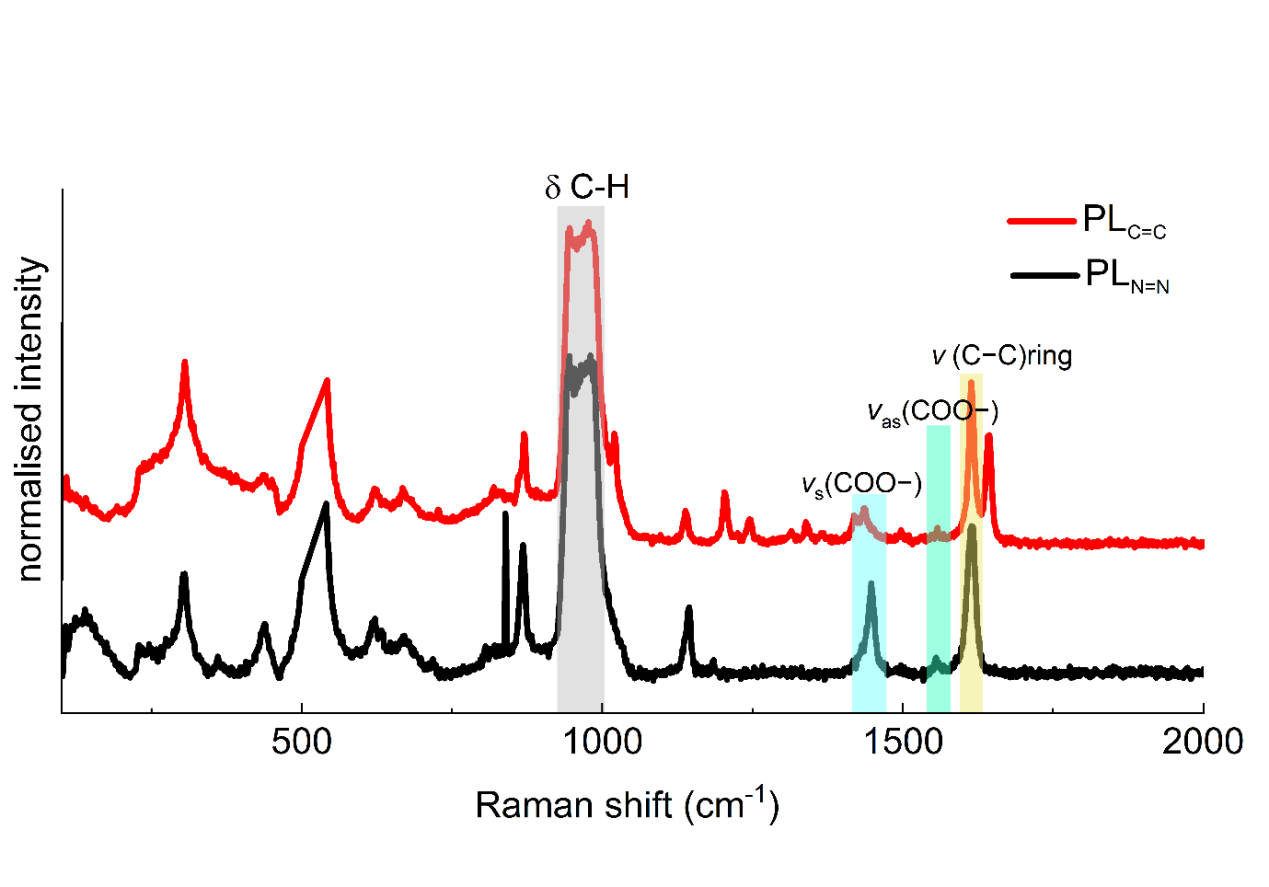


**Supplementary Figure 3**: Raman spectra of PL_C=C_ thin film





**Supplementary Figure 4**: Out-of-plane PXRD of PL_C=C_ thin films


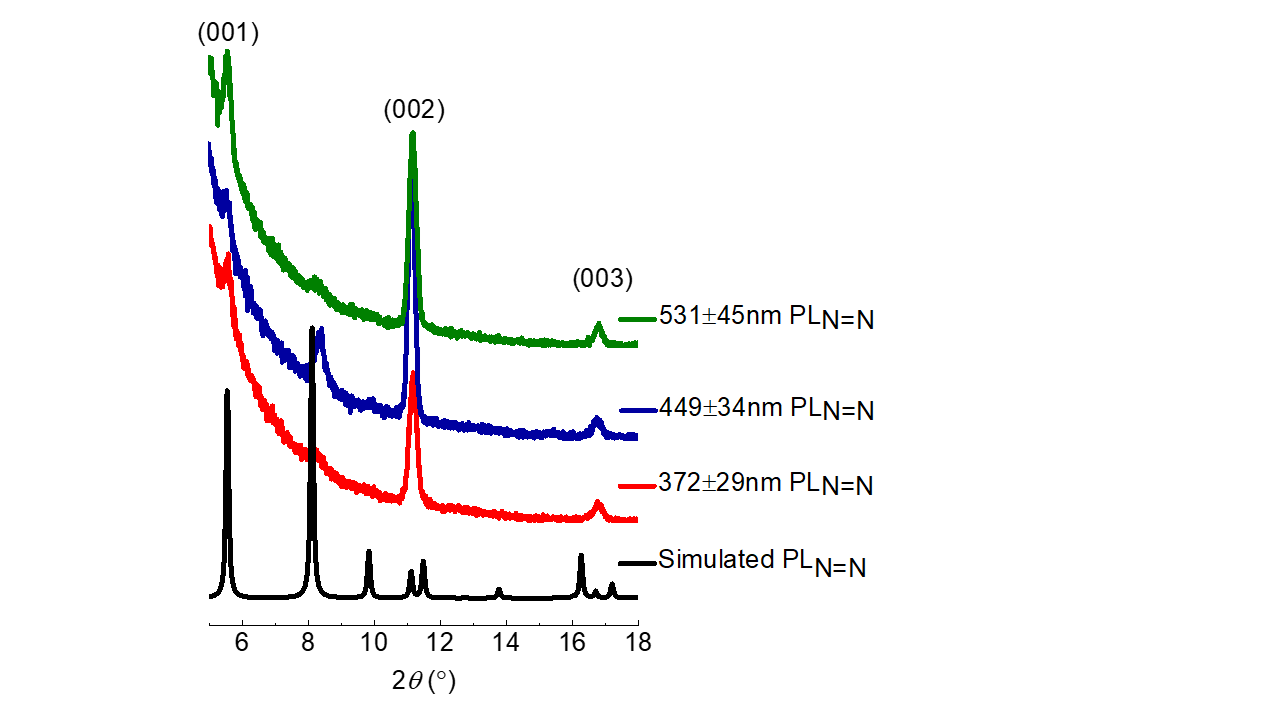


**Supplementary Figure 5**: Out-of-plane PXRD of PL_N=N_ thin films


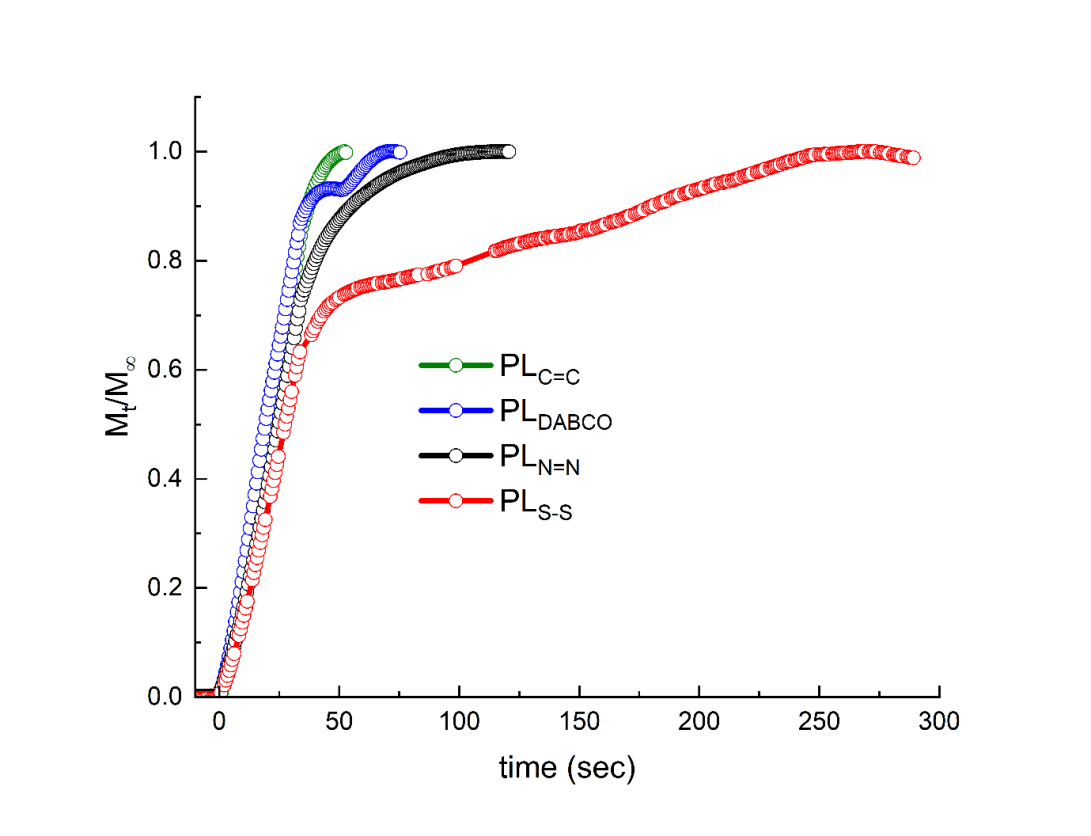


**Supplementary Figure 6**: Fractional mass uptake profile of methanol vapour for PL_C=C_, PL_N=N_, PL_DABCO_ and PL_S-S_ thin films at 298 K.


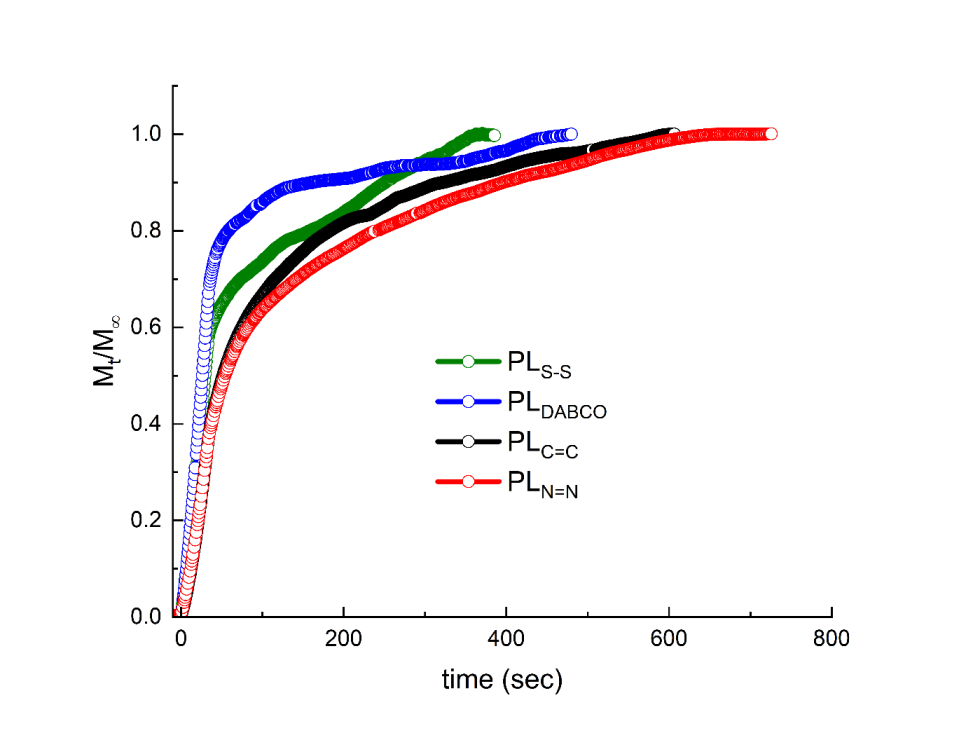


**Supplementary Figure 7**: Fractional mass uptake profile of 1-butanol for PL_C=C_, PL_N=N_, PL_DABCO_ and PL_S-S_ thin films at 298 K.


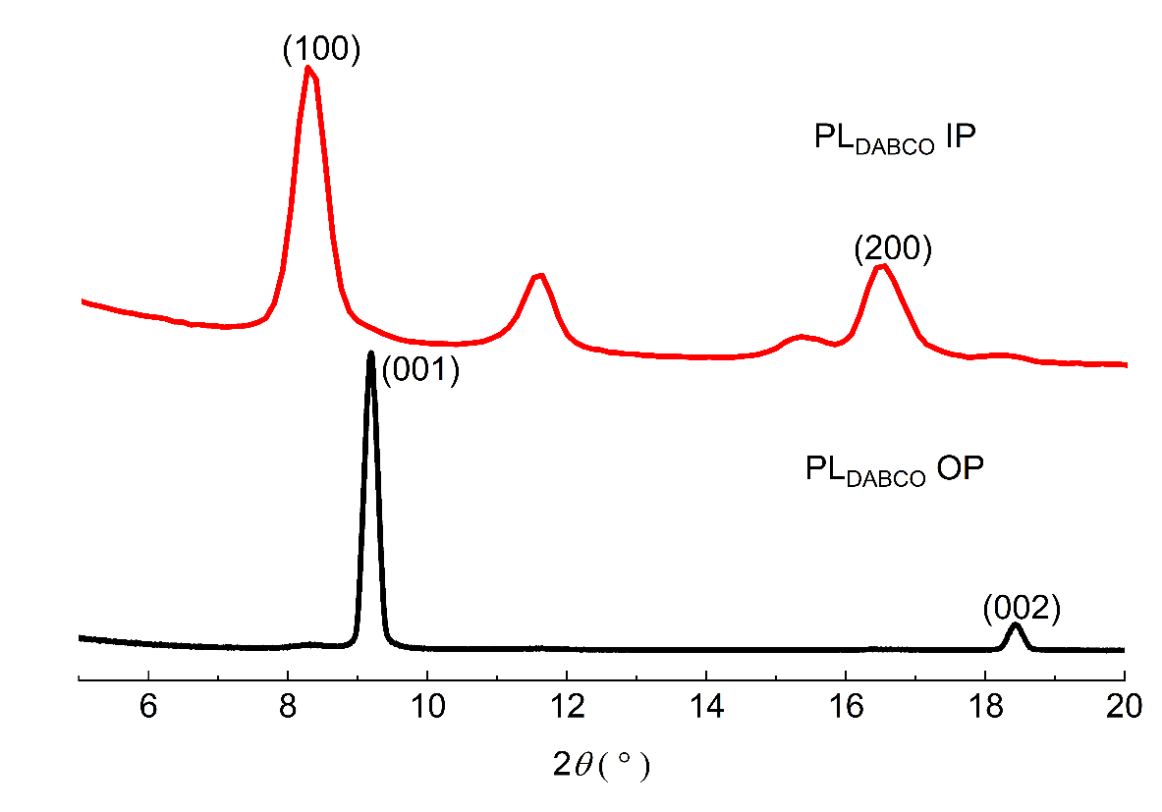


**Supplementary Figure 8**: Out and in-plane PXRD of PL_DABCO_ thin film.


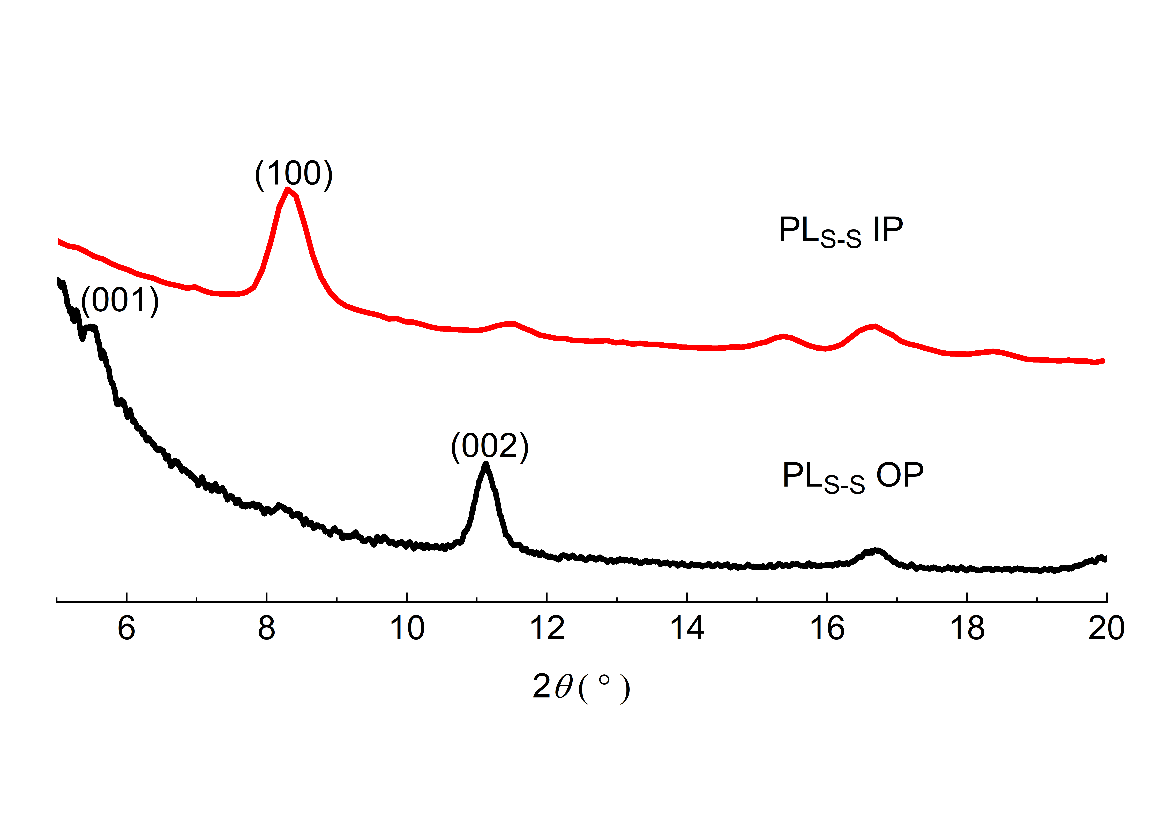


**Supplementary Figure 9**: Out and in-plane PXRD of PL_S-S_ thin film.


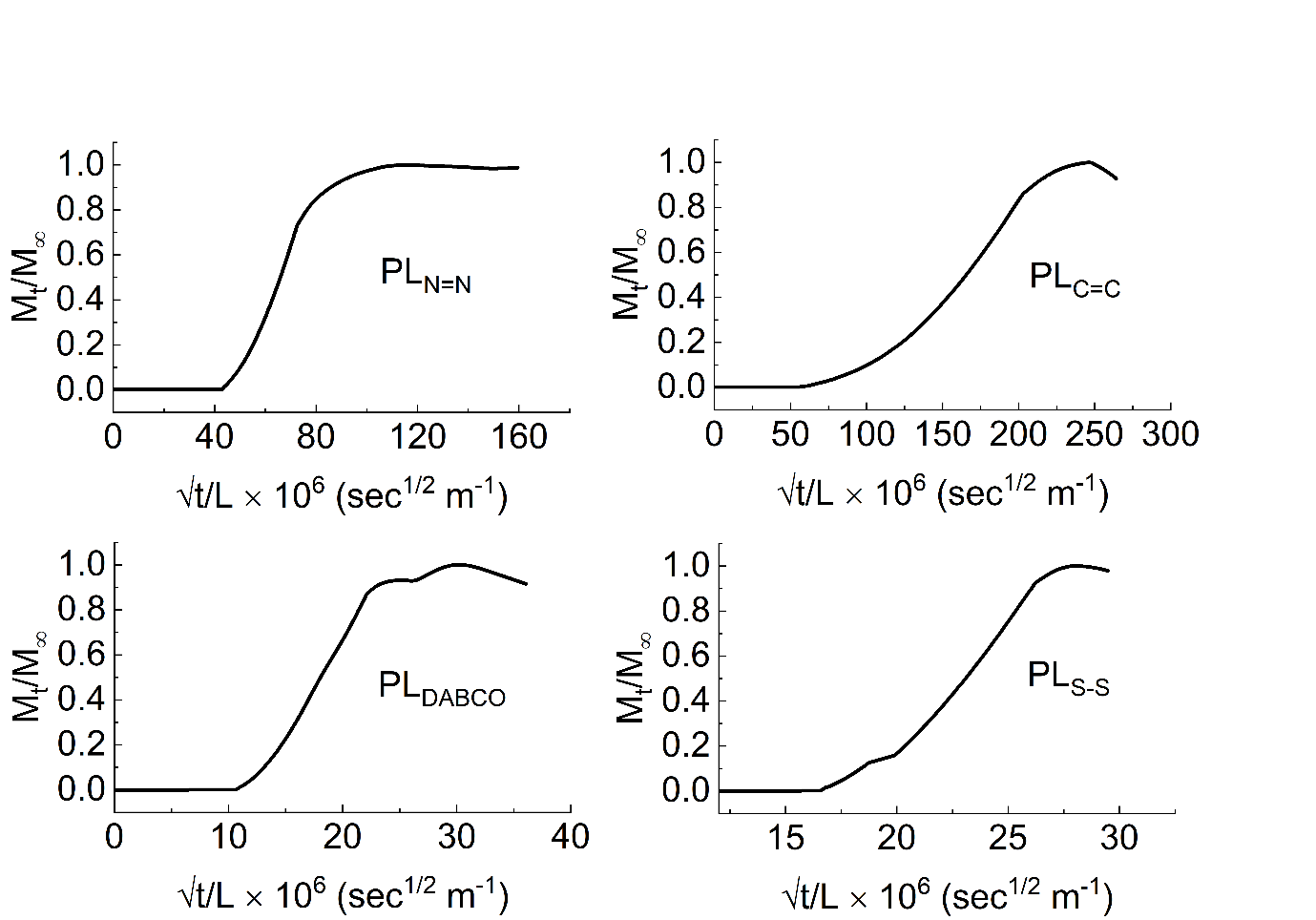


**Supplementary Figure 10**: Fractional mass uptake plots of all the PL thin films at 298 K for methanol.


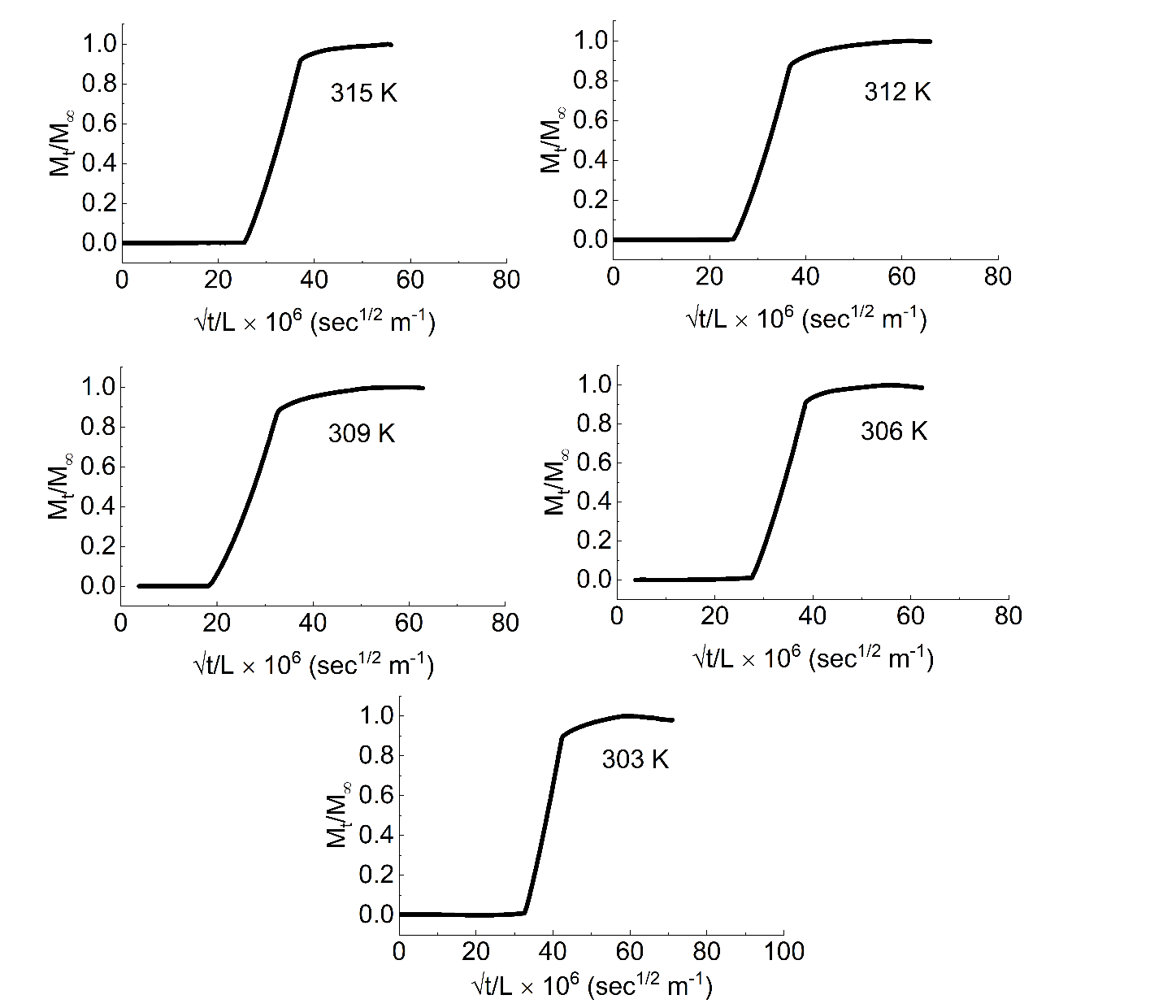


**Supplementary Figure 11**: Fractional mass uptake plots of methanol for PL_C=C_ at different temperatures.


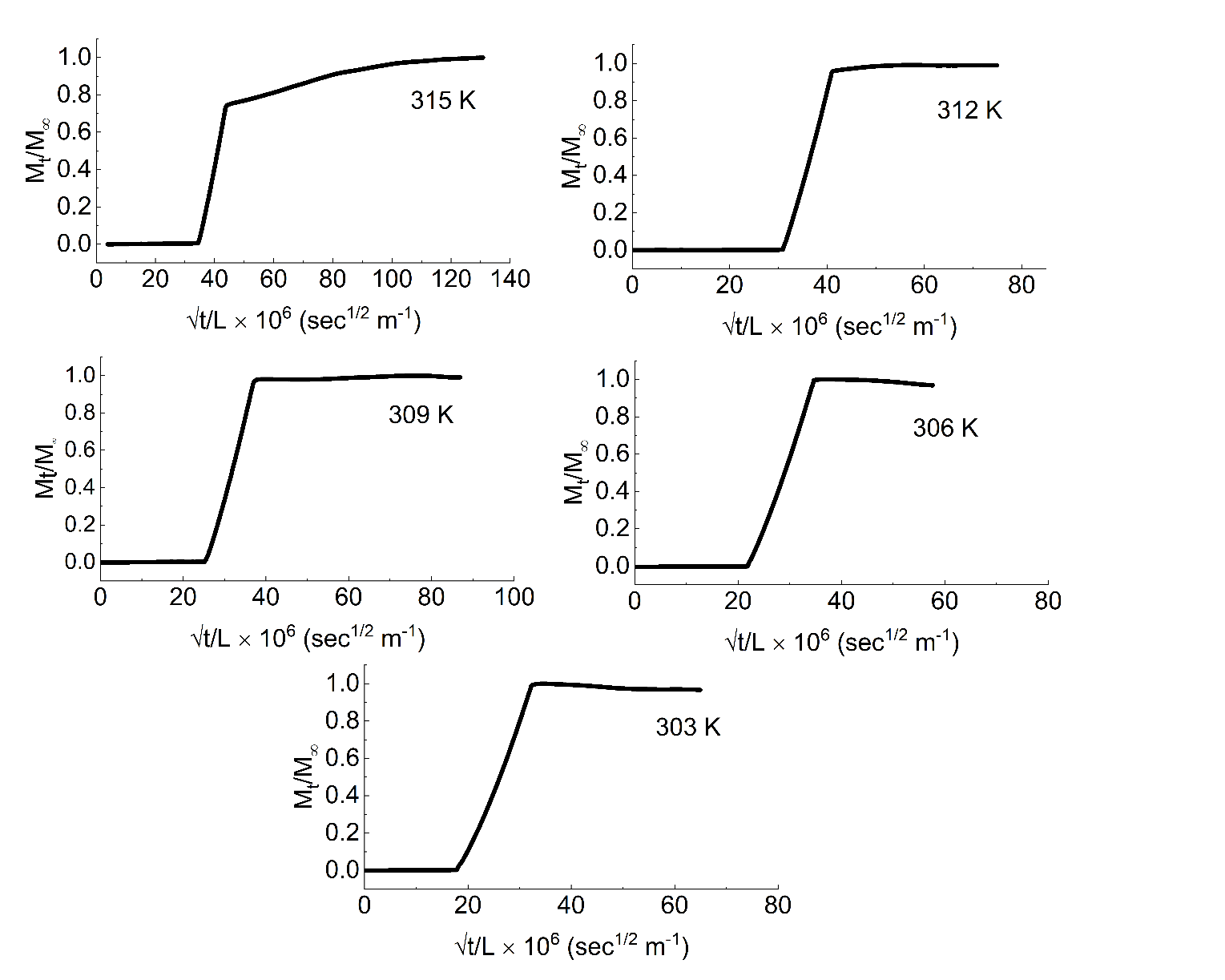


**Supplementary Figure 12**: Fractional mass uptake plots of methanol for PL_N=N_ at different temperatures.

**Supplementary Table 1**: Mass uptake time ratio of 1-butanol/ methanol for different PL thin films at 298 K.

| **PLs** | **Selectivity at lower uptake (20%)** | **Selectivity at saturation uptake** |
| --- | --- | --- |
| PL_C=C_ | 1.9 | 11.5 |
| PL_N=N_ | 1.7 | 5.9 |
| PL_S-S_ | 1.1 | 8.2 |
| PL_DABCO_ | 1.4 | 6.6 |


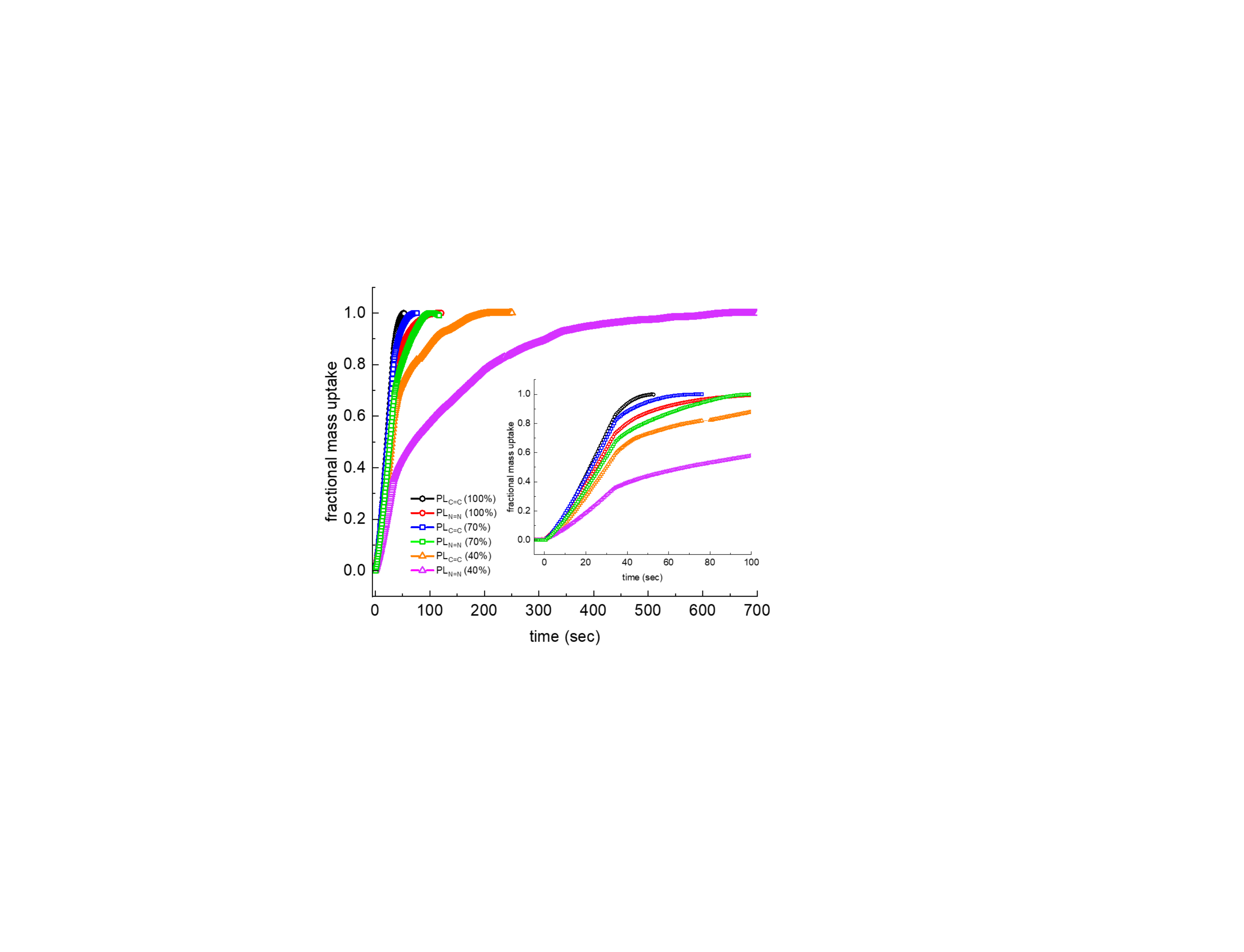


**Supplementary Figure 13**: Fractional mass uptake profiles of methanol vapour at different concentration gradients controlled by varying the nitrogen flow (100% = 50 sccm, 70% = 35 sccm, 40% = 20 sccm) at 298 K.

**Supplementary Table 2**: MD Force field parameters.

| **_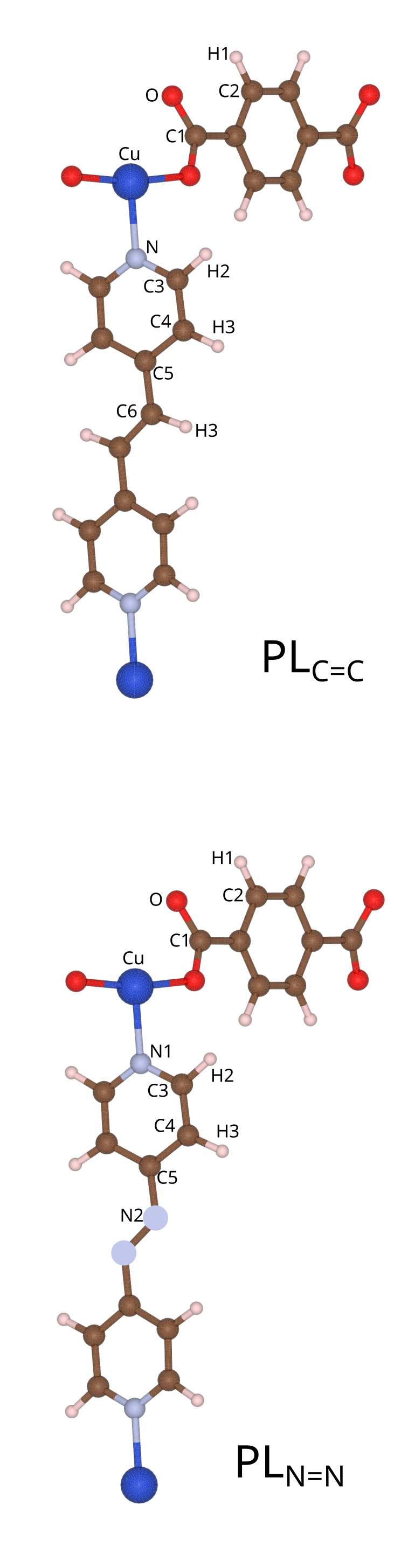_PL_C=C_**  Atom Charge LJ σ (nm) LJ ε (kJ/mol) |
| --- |
| C1 0.550 3.431e-01 0.4393 |
| C2 -0.020 3.431e-01 0.4393 |
| C3 0.330 3.431e-01 0.4393 |
| C4 -0.320 3.431e-01 0.4393 |
| C5 0.290 3.431e-01 0.4393 |
| C6 -0.210 3.431e-01 0.4393 |
| O -0.500 3.118e-01 0.2510 |
| Cu 0.710 3.495e-01 0.0209 |
| N -0.300 3.261e-01 0.2800 |
| H1 0.040 2.571e-01 0.1841 |
| H2 -0.040 2.571e-01 0.1841 |
| H3 0.150 2.571e-01 0.1841 |
| PL_N=N_  Atom Charge LJ σ (nm) LJ ε (kJ/mol) |
| C1 0.550 3.431e-01 0.4393 |
| C2 -0.020 3.431e-01 0.4393 |
| C3 0.370 3.431e-01 0.4393 |
| C4 -0.360 3.431e-01 0.4393 |
| C5 0.600 3.431e-01 0.4393 |
| O -0.500 3.118e-01 0.2510 |
| Cu 0.710 3.495e-01 0.02092 |
| N1 -0.310 3.261e-01 0.28 |
| N2 -0.250 3.261e-01 0.28 |
| H1 0.040 2.571e-01 0.1841 |
| H2 -0.065 2.571e-01 0.1841 |
| H3 0.085 2.571e-01 0.1841 |


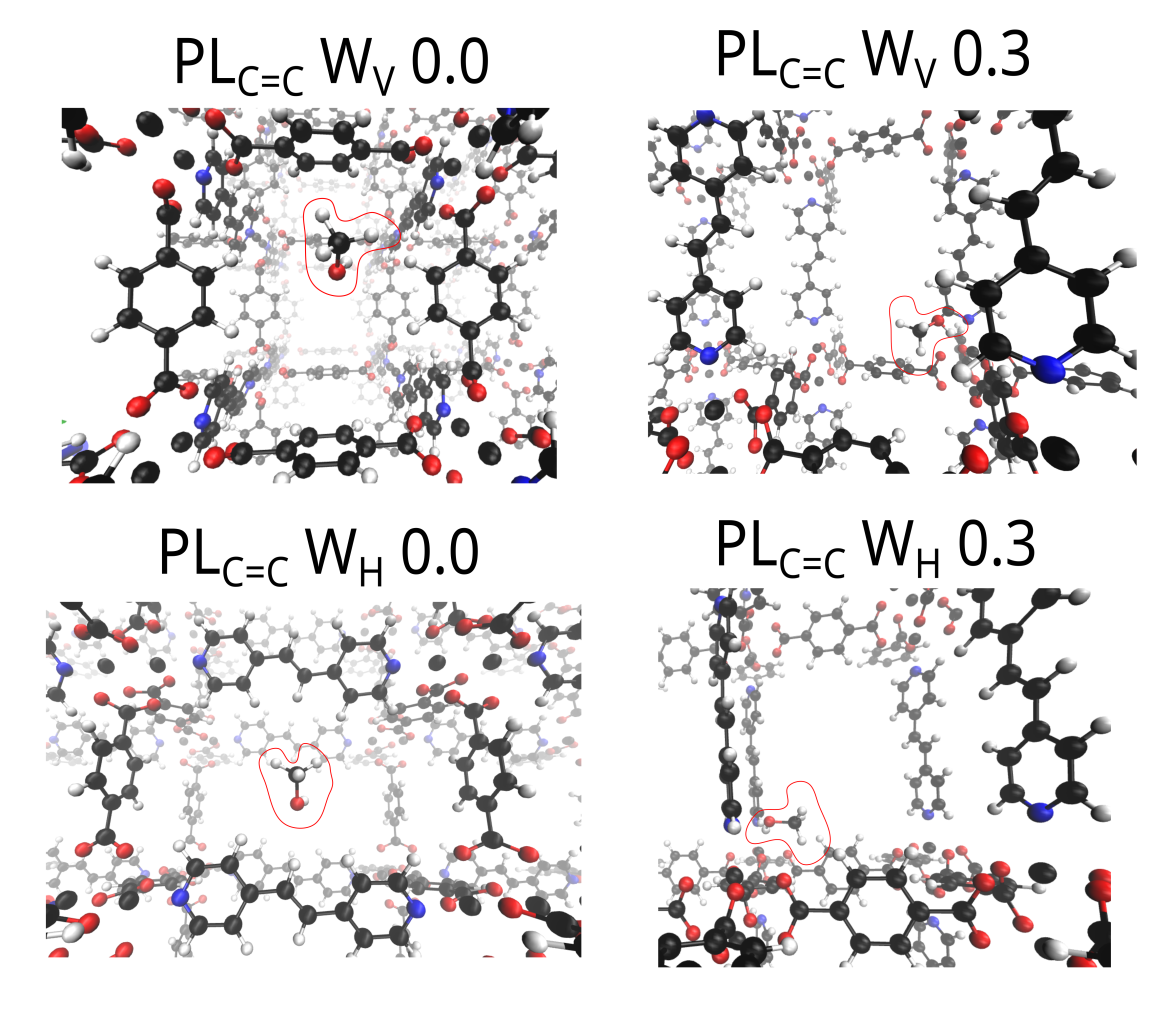


**Supplementary Figure 14**: Snapshots of simulated structures at positions 0.0 (pore) and 0.3 (local minima) for the PL_C=C_ system.


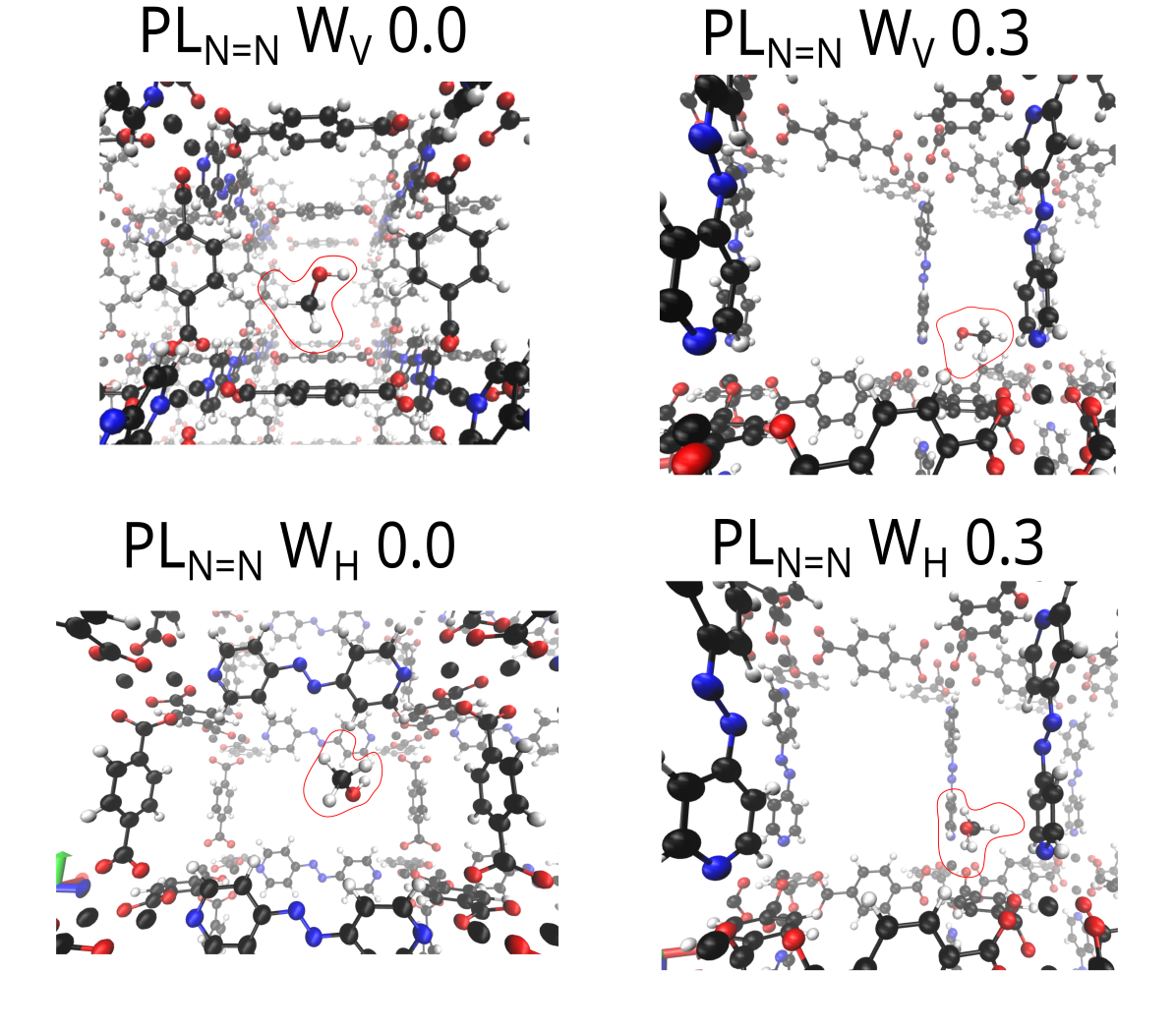


**Supplementary Figure 15**: Snapshots of simulated structures at positions 0.0 (pore) and 0.3 (local minima) for the PL_N=N_ system.


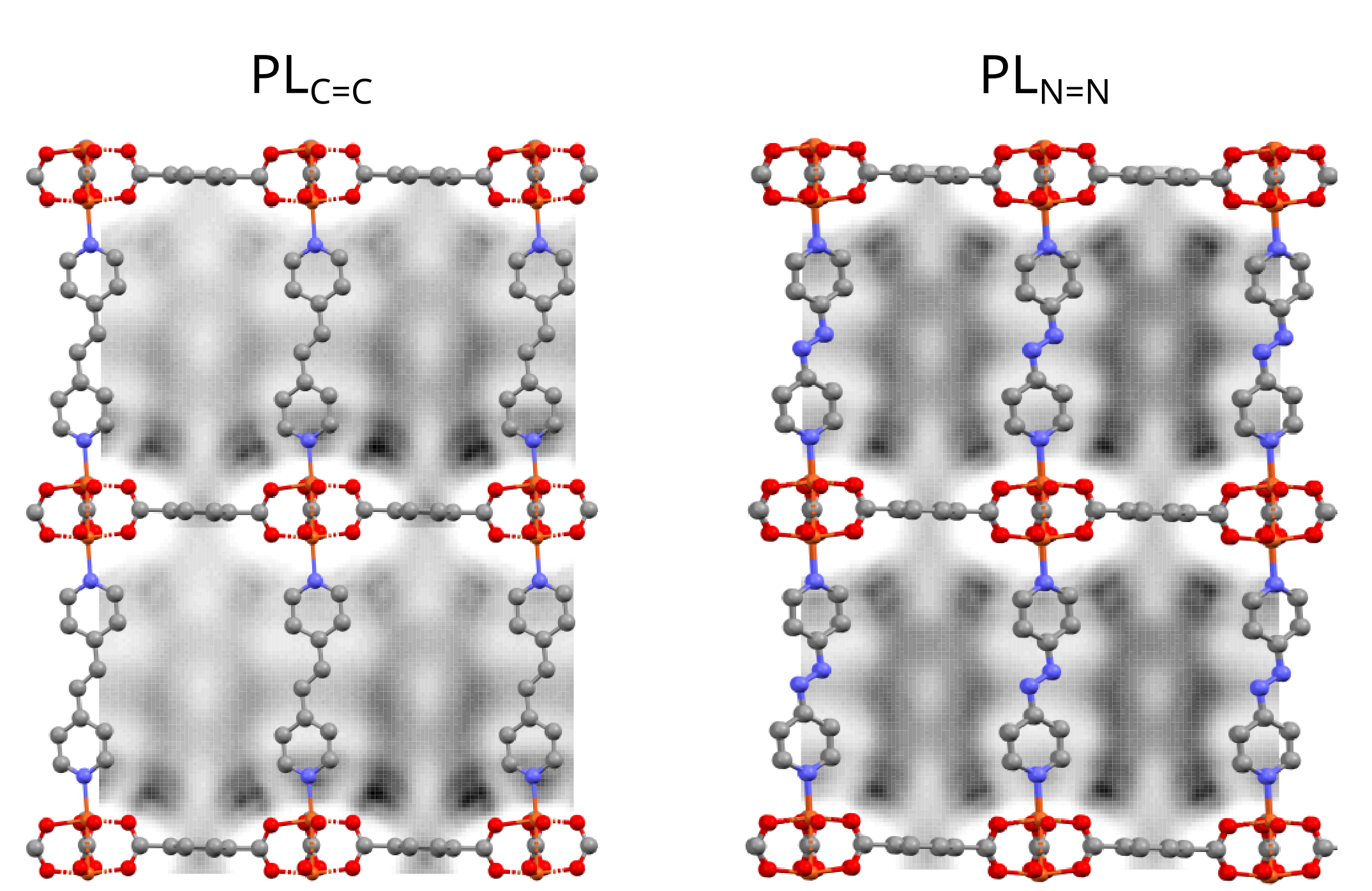
 **Supplementary Figure 16**: Time averaged density profiles for 100 methanol molecules in both the PL systems, obtained from a 1 microsecond unbiased simulation.

**Coordinates are in Ångstroms**

Single unit of the PL_C=C_

Optimized cell parameters: A: 10.891 B: 10.891 C: 16.303

O 4.0355767965650751E+00 9.0229313076087898E+00 1.5769587843479558E+00

O 4.0156555578360162E+00 8.3493147386054218E+00 -6.1205794803947178E-01

O 1.0994502611416529E+01 9.0059136022863644E+00 -6.1287017293301327E-01

O 1.0977265000324373E+01 8.3415315674767534E+00 1.5803337523360765E+00

Cu 2.0561216674454017E+00 8.7046214065121976E+00 1.8438977730329535E+00

Cu 2.0548842892947565E+00 8.6810380844048165E+00 1.5528102707170511E+01

C 1.0417021643117707E+01 8.6689084700941059E+00 4.8103155301195266E-01

C 8.9112788094754922E+00 8.6654994542099786E+00 4.6755860260700932E-01

C 4.5952570834302353E+00 8.6835277718630177E+00 4.8091390014558194E-01

C 6.1016857483353428E+00 8.6727598436086168E+00 4.6878067775640364E-01

C 8.2097511977355246E+00 8.6530153905374618E+00 -7.6006409393467278E-01

C 6.8026523974851827E+00 8.6385052730208010E+00 -7.5905276757274931E-01

C 6.8042923239010857E+00 8.6999512465469824E+00 1.6952945972902993E+00

C 8.2098291046826493E+00 8.6784142009942826E+00 1.6950009606741854E+00

N 2.0503534250217776E+00 8.8433997823156520E+00 3.9463047214346623E+00

C 2.2945275047895377E+00 7.7514903024097244E+00 4.7136768897154173E+00

C 2.3159950178555255E+00 7.7940311290985491E+00 6.1143996402494860E+00

C 2.0684575387711237E+00 9.0288344222241221E+00 6.7755314575015202E+00

C 1.8173090715681472E+00 1.0162856392814284E+01 5.9587425155382396E+00

C 1.8199178086379715E+00 1.0027378435083499E+01 4.5609730550288008E+00

C 2.0380693211839893E+00 9.1832080958953011E+00 8.2322811174943933E+00

C 2.3164287559365748E+00 8.2041635786552014E+00 9.1440759417007538E+00

C 2.2372237192882141E+00 8.3550773255596642E+00 1.0600089863602495E+01

C 1.7336413963970001E+00 9.5197616853380804E+00 1.1243230246396944E+01

C 1.6866041296067555E+00 9.5604206809733387E+00 1.2643024275719938E+01

N 2.0942606901025456E+00 8.5311190631388030E+00 1.3424296529905654E+01

C 2.5733525690356398E+00 7.4155321745814557E+00 1.2828347884014342E+01

C 2.6635941978956681E+00 7.2857177115968277E+00 1.1432217835922536E+01

H 2.4701935975321492E+00 6.8193621885527440E+00 4.1504304674867232E+00

H 2.5103165418474345E+00 6.8692599606287947E+00 6.6782571151272476E+00

H 1.6193793095803550E+00 1.1145401141829804E+01 6.4166909178995128E+00

H 1.6338077592664479E+00 1.0875794216698162E+01 3.8820117811196768E+00

H 1.3677722382675443E+00 1.0382453096366129E+01 1.0666423366550733E+01

H 1.3128580001317631E+00 1.0441404273143979E+01 1.3190885290062889E+01

H 2.8773942908827168E+00 6.6116803495332617E+00 1.3518865238072557E+01

H 3.0610343515649792E+00 6.3592640191569911E+00 1.0987028667674323E+01

H 6.2302312155837996E+00 8.7324301244193787E+00 2.6326221377680223E+00

H 6.2284476124887913E+00 8.6121459596425733E+00 -1.6967786410147740E+00

H 8.7831117357714792E+00 8.6468586243448762E+00 -1.6987067000284859E+00

H 8.7851388260180094E+00 8.6782526907996616E+00 2.6321311579617372E+00

O 1.7500617802810878E+00 6.7007912731456365E+00 1.5819609391383992E+01

O 2.4144395723959460E+00 6.7273938539931404E+00 1.8015974303802061E+01

O 1.7221227030703932E+00 -2.2575896299353360E-01 1.7890454595900039E+01

O 2.3648557882899186E+00 -2.1577467105211537E-01 1.5688252798357542E+01

C 2.0502065660524780E+00 3.3850548335360081E-01 1.6791197443999295E+01

C 2.0874924769790657E+00 4.6477977096319893E+00 1.6916852354186609E+01

C 8.5633649974383419E-01 3.9603432290632123E+00 1.6847554414007973E+01

C 8.4385139972109469E-01 2.5523723146866910E+00 1.6824443153584511E+01

C 2.0645130032280452E+00 1.8438479398201850E+00 1.6823603927161560E+01

C 3.2965446106422340E+00 2.5302302765545899E+00 1.6876485161069187E+01

C 3.3074166589339900E+00 3.9383859247052984E+00 1.6944705873355282E+01

H -8.5718022535864830E-02 4.5289307123941187E+00 1.6819391061995770E+01

H -1.0923692964738968E-01 2.0020534155384766E+00 1.6796051498378898E+01

H 4.2403390134761132E+00 1.9631960694161044E+00 1.6874963020355178E+01

H 4.2604222184298912E+00 4.4863102058858200E+00 1.7005641861966918E+01

C 2.0870079415584342E+00 6.1541868895886731E+00 1.6922371574622492E+01

H 2.6294901004134985E+00 7.2071745872924833E+00 8.7877051542790579E+00

H 1.7612884845172245E+00 1.0189953198551555E+01 8.5912693592116707E+00

**Single unit of the PL_N=N_**

Optimized cell parameters: A: 10.892 B:10.892 C:15.914

O 4.1540896959974791E+00 8.9568332099352297E+00 1.5975108863258189E+00

O 4.1529486971548026E+00 8.3277435310949759E+00 -6.0572144684019369E-01

O 1.1107613152654128E+01 8.9452791798700435E+00 -6.0446193611153876E-01

O 1.1113970904337245E+01 8.3186413170205906E+00 1.6001759222606342E+00

Cu 2.1880518015389789E+00 8.6458978342741464E+00 1.8175603995298601E+00

Cu 2.1831031203292519E+00 8.6335412774019389E+00 1.5096303426834286E+01

C 1.0542897477688198E+01 8.6275855300464901E+00 4.9888983224910777E-01

C 9.0366731916961971E+00 8.6198215296288421E+00 5.0054465167500051E-01

C 4.7222696156146027E+00 8.6369709254928129E+00 4.9764140931489159E-01

C 6.2278980489525333E+00 8.6250173958122129E+00 4.9990699398784033E-01

C 8.3354718425132255E+00 8.6052418795149315E+00 -7.2710085156721582E-01

C 6.9294972834699777E+00 8.5905980247064750E+00 -7.2718591551482803E-01

C 6.9289265503451327E+00 8.6528951354201382E+00 1.7275900710787189E+00

C 8.3352684595394049E+00 8.6323313273936115E+00 1.7284673335174063E+00

N 2.1627522322854560E+00 8.7748207514225030E+00 3.9175447568816475E+00

C 2.4134021625447906E+00 7.6799084147594483E+00 4.6815775328468012E+00

C 2.4420408466985544E+00 7.7225685940475000E+00 6.0826325054251171E+00

C 2.1944002803594707E+00 8.9688720751585613E+00 6.7158649506149608E+00

C 1.9415859789859060E+00 1.0110130460068525E+01 5.9198628485308911E+00

C 1.9388877306157577E+00 9.9650126777931813E+00 4.5211158890861496E+00

N 2.1514947503318531E+00 9.1759338364731899E+00 8.1218703789180022E+00

N 2.4486596711933335E+00 8.1351358410181192E+00 8.8009436435983908E+00

C 2.3534000376202946E+00 8.3336094968562264E+00 1.0206096118469377E+01

C 1.8385203550501941E+00 9.5029195563614302E+00 1.0825439692606343E+01

C 1.7895019070414628E+00 9.5358774735238949E+00 1.2225916207696280E+01

N 2.2014490585784756E+00 8.5000285963421085E+00 1.3000541567899617E+01

C 2.6913603453344486E+00 7.3862609399840835E+00 1.2410256098511621E+01

C 2.7871763854011880E+00 7.2557478021672113E+00 1.1013014537580947E+01

H 2.5857535493090817E+00 6.7503945788123136E+00 4.1122451425151425E+00

H 2.6395058548584691E+00 6.8255054151151660E+00 6.6861813606964091E+00

H 1.7484773373359934E+00 1.1080938316891137E+01 6.4001865474301294E+00

H 1.7502538378231784E+00 1.0807812426221762E+01 3.8351797211393328E+00

H 1.4875235389525931E+00 1.0344571837878650E+01 1.0211572174622757E+01

H 1.4132345814561287E+00 1.0410457637231280E+01 1.2782878674744381E+01

H 2.9940391872659569E+00 6.5845136071001615E+00 1.3104274217626564E+01

H 3.1813378849171703E+00 6.3427306636448213E+00 1.0541976279054397E+01

H 6.3545573825649866E+00 8.6851911958392272E+00 2.6648628899409075E+00

H 6.3563141124745899E+00 8.5631357127856411E+00 -1.6652244520138453E+00

H 8.9095152656074514E+00 8.5973656891128272E+00 -1.6650891959092595E+00

H 8.9076722999034104E+00 8.6317871967435931E+00 2.6675376894031975E+00

O 1.8838795310441390E+00 6.6574322872212788E+00 1.5371930129271512E+01

O 2.5437066909957426E+00 6.6636745558743353E+00 1.7570362928197287E+01

O 1.8458324626795652E+00 -2.7470234831995838E-01 1.7452751693897987E+01

O 2.4844582812441733E+00 -2.7446431530958604E-01 1.5249983303439192E+01

C 2.1703721860051264E+00 2.8524265815894712E-01 1.6352070013971321E+01

C 2.2122787669317741E+00 4.5952317390393009E+00 1.6457578586002011E+01

C 9.7971466556470965E-01 3.9102547250503550E+00 1.6392362163453384E+01

C 9.6491820712399656E-01 2.5020446045420544E+00 1.6373788872246298E+01

C 2.1842989887003683E+00 1.7915937182735373E+00 1.6374250374755523E+01

C 3.4174045931720514E+00 2.4763094289047083E+00 1.6418317636577147E+01

C 3.4312966605070363E+00 3.8844416778743738E+00 1.6481781427606066E+01

H 3.8417181640769210E-02 4.4800029020005558E+00 1.6364497218754636E+01

H 1.0378624781884883E-02 1.9545278274504592E+00 1.6348042796242883E+01

H 4.3596080064559635E+00 1.9071287991488417E+00 1.6413465894311035E+01

H 4.3864639799249758E+00 4.4291535520899092E+00 1.6536589311360423E+01

C 2.2162013010990274E+00 6.1013975299909715E+00 1.6472275459554968E+01
