# Supplementary material for: Chemically routed interpore molecular diffusion in metal-organic framework thin films
Source: Nat Commun. 2023 Apr 18;14:2212. doi: 10.1038/s41467-023-37739-8 (PMC10113335; doi:10.1038/s41467-023-37739-8)
Supplement: Supplementary file 3 — Description of Additional Supplementary Files [file 41467_2023_37739_MOESM3_ESM.docx]

**Description of Additional Supplementary Files**

Supplementary Data 1:

Description: coordinates for simulated structure of both PLN=N and PLC=C for the calculation of binding energy for methanol. Snapshots of the simulated structure using these coordinates are given in supplementary Figure 14 and 15.
