# Supplementary material for: Chemically routed interpore molecular diffusion in metal-organic framework thin films
Source: Nat Commun. 2023 Apr 18;14:2212. doi: 10.1038/s41467-023-37739-8 (PMC10113335; doi:10.1038/s41467-023-37739-8)
Supplement: Supplementary file 4 — Supplementary dataset 1 [file 41467_2023_37739_MOESM4_ESM.pdf]

Supplementary Dataset 1

Chemically routed interpore molecular diffusion in metal-organic framework thin films

Tanmoy Maity[1,±] Pratibha Malik[1,±] Sumit Bawari[1], Soumya Ghosh[1], Jagannath Mondal[1], Ritesh Haldar[1✉]

[±] Contributed equally

✉Email: riteshhaldar@tifrh.res.in

[1]Tata Institute of Fundamental Research Hyderabad, Gopanpally, Hyderabad 500046, Telangana, India

Atomic Coordinates are in Ångstroms

Coordinates for $PL_{N=N}$ for the calculation of binding energy for methanol

CRYST1 32.676 32.676 100.000 90.00 90.00 90.00 P 1 1

ATOM 1 O CuN X 1 5.670 7.498 27.220 0.00 0.00

ATOM 2 O CuN X 1 5.670 7.498 43.140 0.00 0.00

ATOM 3 O CuN X 1 5.670 7.498 59.050 0.00 0.00

ATOM 4 O CuN X 1 5.670 7.498 74.970 0.00 0.00

ATOM 5 O CuN X 1 5.670 18.388 27.220 0.00 0.00

ATOM 6 O CuN X 1 5.670 18.388 43.140 0.00 0.00

ATOM 7 O CuN X 1 5.670 18.388 59.050 0.00 0.00

ATOM 8 O CuN X 1 5.670 18.388 74.970 0.00 0.00

ATOM 9 O CuN X 1 5.670 29.278 27.220 0.00 0.00

ATOM 10 O CuN X 1 5.670 29.278 43.140 0.00 0.00

ATOM 11 O CuN X 1 5.670 29.278 59.050 0.00 0.00

ATOM 12 O CuN X 1 5.670 29.278 74.970 0.00 0.00

ATOM 13 O CuN X 1 16.570 7.498 27.220 0.00 0.00

ATOM 14 O CuN X 1 16.570 7.498 43.140 0.00 0.00

ATOM 15 O CuN X 1 16.570 7.498 59.050 0.00 0.00

ATOM 16 O CuN X 1 16.570 7.498 74.970 0.00 0.00

ATOM 17 O CuN X 1 16.570 18.388 27.220 0.00 0.00

ATOM	18	O	CuN	X	1	16.570	18.388	43.140	0.00	0.00
ATOM	19	O	CuN	X	1	16.570	18.388	59.050	0.00	0.00
ATOM	20	O	CuN	X	1	16.570	18.388	74.970	0.00	0.00
ATOM	21	O	CuN	X	1	16.570	29.278	27.220	0.00	0.00
ATOM	22	O	CuN	X	1	16.570	29.278	43.140	0.00	0.00
ATOM	23	O	CuN	X	1	16.570	29.278	59.050	0.00	0.00
ATOM	24	O	CuN	X	1	16.570	29.278	74.970	0.00	0.00
ATOM	25	O	CuN	X	1	27.460	7.498	27.220	0.00	0.00
ATOM	26	O	CuN	X	1	27.460	7.498	43.140	0.00	0.00
ATOM	27	O	CuN	X	1	27.460	7.498	59.050	0.00	0.00
ATOM	28	O	CuN	X	1	27.460	7.498	74.970	0.00	0.00
ATOM	29	O	CuN	X	1	27.460	18.388	27.220	0.00	0.00
ATOM	30	O	CuN	X	1	27.460	18.388	43.140	0.00	0.00
ATOM	31	O	CuN	X	1	27.460	18.388	59.050	0.00	0.00
ATOM	32	O	CuN	X	1	27.460	18.388	74.970	0.00	0.00
ATOM	33	O	CuN	X	1	27.460	29.278	27.220	0.00	0.00
ATOM	34	O	CuN	X	1	27.460	29.278	43.140	0.00	0.00
ATOM	35	O	CuN	X	1	27.460	29.278	59.050	0.00	0.00
ATOM	36	O	CuN	X	1	27.460	29.278	74.970	0.00	0.00
ATOM	37	O	CuN	X	1	5.670	6.868	25.020	0.00	0.00
ATOM	38	O	CuN	X	1	5.670	6.868	40.930	0.00	0.00
ATOM	39	O	CuN	X	1	5.670	6.868	56.850	0.00	0.00
ATOM	40	O	CuN	X	1	5.670	6.868	72.760	0.00	0.00
ATOM	41	O	CuN	X	1	5.670	17.758	25.020	0.00	0.00
ATOM	42	O	CuN	X	1	5.670	17.758	40.930	0.00	0.00
ATOM	43	O	CuN	X	1	5.670	17.758	56.850	0.00	0.00
ATOM	44	O	CuN	X	1	5.670	17.758	72.760	0.00	0.00
ATOM	45	O	CuN	X	1	5.670	28.648	25.020	0.00	0.00
ATOM	46	O	CuN	X	1	5.670	28.648	40.930	0.00	0.00
ATOM	47	O	CuN	X	1	5.670	28.648	56.850	0.00	0.00

ATOM	48	O	CuN	X	1	5.670	28.648	72.760	0.00	0.00
ATOM	49	O	CuN	X	1	16.560	6.868	25.020	0.00	0.00
ATOM	50	O	CuN	X	1	16.560	6.868	40.930	0.00	0.00
ATOM	51	O	CuN	X	1	16.560	6.868	56.850	0.00	0.00
ATOM	52	O	CuN	X	1	16.560	6.868	72.760	0.00	0.00
ATOM	53	O	CuN	X	1	16.560	17.758	25.020	0.00	0.00
ATOM	54	O	CuN	X	1	16.560	17.758	40.930	0.00	0.00
ATOM	55	O	CuN	X	1	16.560	17.758	56.850	0.00	0.00
ATOM	56	O	CuN	X	1	16.560	17.758	72.760	0.00	0.00
ATOM	57	O	CuN	X	1	16.560	28.648	25.020	0.00	0.00
ATOM	58	O	CuN	X	1	16.560	28.648	40.930	0.00	0.00
ATOM	59	O	CuN	X	1	16.560	28.648	56.850	0.00	0.00
ATOM	60	O	CuN	X	1	16.560	28.648	72.760	0.00	0.00
ATOM	61	O	CuN	X	1	27.460	6.868	25.020	0.00	0.00
ATOM	62	O	CuN	X	1	27.460	6.868	40.930	0.00	0.00
ATOM	63	O	CuN	X	1	27.460	6.868	56.850	0.00	0.00
ATOM	64	O	CuN	X	1	27.460	6.868	72.760	0.00	0.00
ATOM	65	O	CuN	X	1	27.460	17.758	25.020	0.00	0.00
ATOM	66	O	CuN	X	1	27.460	17.758	40.930	0.00	0.00
ATOM	67	O	CuN	X	1	27.460	17.758	56.850	0.00	0.00
ATOM	68	O	CuN	X	1	27.460	17.758	72.760	0.00	0.00
ATOM	69	O	CuN	X	1	27.460	28.648	25.020	0.00	0.00
ATOM	70	O	CuN	X	1	27.460	28.648	40.930	0.00	0.00
ATOM	71	O	CuN	X	1	27.460	28.648	56.850	0.00	0.00
ATOM	72	O	CuN	X	1	27.460	28.648	72.760	0.00	0.00
ATOM	73	O	CuN	X	1	12.630	7.488	25.020	0.00	0.00
ATOM	74	O	CuN	X	1	12.630	7.488	40.940	0.00	0.00
ATOM	75	O	CuN	X	1	12.630	7.488	56.850	0.00	0.00
ATOM	76	O	CuN	X	1	12.630	7.488	72.760	0.00	0.00
ATOM	77	O	CuN	X	1	12.630	18.378	25.020	0.00	0.00

ATOM	78	O	CuN	X	1	12.630	18.378	40.940	0.00	0.00
ATOM	79	O	CuN	X	1	12.630	18.378	56.850	0.00	0.00
ATOM	80	O	CuN	X	1	12.630	18.378	72.760	0.00	0.00
ATOM	81	O	CuN	X	1	12.630	29.268	25.020	0.00	0.00
ATOM	82	O	CuN	X	1	12.630	29.268	40.940	0.00	0.00
ATOM	83	O	CuN	X	1	12.630	29.268	56.850	0.00	0.00
ATOM	84	O	CuN	X	1	12.630	29.268	72.760	0.00	0.00
ATOM	85	O	CuN	X	1	23.520	7.488	25.020	0.00	0.00
ATOM	86	O	CuN	X	1	23.520	7.488	40.940	0.00	0.00
ATOM	87	O	CuN	X	1	23.520	7.488	56.850	0.00	0.00
ATOM	88	O	CuN	X	1	23.520	7.488	72.760	0.00	0.00
ATOM	89	O	CuN	X	1	23.520	18.378	25.020	0.00	0.00
ATOM	90	O	CuN	X	1	23.520	18.378	40.940	0.00	0.00
ATOM	91	O	CuN	X	1	23.520	18.378	56.850	0.00	0.00
ATOM	92	O	CuN	X	1	23.520	18.378	72.760	0.00	0.00
ATOM	93	O	CuN	X	1	23.520	29.268	25.020	0.00	0.00
ATOM	94	O	CuN	X	1	23.520	29.268	40.940	0.00	0.00
ATOM	95	O	CuN	X	1	23.520	29.268	56.850	0.00	0.00
ATOM	96	O	CuN	X	1	23.520	29.268	72.760	0.00	0.00
ATOM	97	O	CuN	X	1	12.630	6.858	27.230	0.00	0.00
ATOM	98	O	CuN	X	1	12.630	6.858	43.140	0.00	0.00
ATOM	99	O	CuN	X	1	12.630	6.858	59.050	0.00	0.00
ATOM	100	O	CuN	X	1	12.630	6.858	74.970	0.00	0.00
ATOM	101	O	CuN	X	1	12.630	17.748	27.230	0.00	0.00
ATOM	102	O	CuN	X	1	12.630	17.748	43.140	0.00	0.00
ATOM	103	O	CuN	X	1	12.630	17.748	59.050	0.00	0.00
ATOM	104	O	CuN	X	1	12.630	17.748	74.970	0.00	0.00
ATOM	105	O	CuN	X	1	12.630	28.638	27.230	0.00	0.00
ATOM	106	O	CuN	X	1	12.630	28.638	43.140	0.00	0.00
ATOM	107	O	CuN	X	1	12.630	28.638	59.050	0.00	0.00

ATOM	108	O	CuN	X	1	12.630	28.638	74.970	0.00	0.00
ATOM	109	O	CuN	X	1	23.530	6.858	27.230	0.00	0.00
ATOM	110	O	CuN	X	1	23.530	6.858	43.140	0.00	0.00
ATOM	111	O	CuN	X	1	23.530	6.858	59.050	0.00	0.00
ATOM	112	O	CuN	X	1	23.530	6.858	74.970	0.00	0.00
ATOM	113	O	CuN	X	1	23.530	17.748	27.230	0.00	0.00
ATOM	114	O	CuN	X	1	23.530	17.748	43.140	0.00	0.00
ATOM	115	O	CuN	X	1	23.530	17.748	59.050	0.00	0.00
ATOM	116	O	CuN	X	1	23.530	17.748	74.970	0.00	0.00
ATOM	117	O	CuN	X	1	23.530	28.638	27.230	0.00	0.00
ATOM	118	O	CuN	X	1	23.530	28.638	43.140	0.00	0.00
ATOM	119	O	CuN	X	1	23.530	28.638	59.050	0.00	0.00
ATOM	120	O	CuN	X	1	23.530	28.638	74.970	0.00	0.00
ATOM	121	Cu	CuN	X	1	3.710	7.188	27.440	0.00	0.00
ATOM	122	Cu	CuN	X	1	3.710	7.188	43.360	0.00	0.00
ATOM	123	Cu	CuN	X	1	3.710	7.188	59.270	0.00	0.00
ATOM	124	Cu	CuN	X	1	3.710	7.188	75.190	0.00	0.00
ATOM	125	Cu	CuN	X	1	3.710	18.078	27.440	0.00	0.00
ATOM	126	Cu	CuN	X	1	3.710	18.078	43.360	0.00	0.00
ATOM	127	Cu	CuN	X	1	3.710	18.078	59.270	0.00	0.00
ATOM	128	Cu	CuN	X	1	3.710	18.078	75.190	0.00	0.00
ATOM	129	Cu	CuN	X	1	3.710	28.968	27.440	0.00	0.00
ATOM	130	Cu	CuN	X	1	3.710	28.968	43.360	0.00	0.00
ATOM	131	Cu	CuN	X	1	3.710	28.968	59.270	0.00	0.00
ATOM	132	Cu	CuN	X	1	3.710	28.968	75.190	0.00	0.00
ATOM	133	Cu	CuN	X	1	14.600	7.188	27.440	0.00	0.00
ATOM	134	Cu	CuN	X	1	14.600	7.188	43.360	0.00	0.00
ATOM	135	Cu	CuN	X	1	14.600	7.188	59.270	0.00	0.00
ATOM	136	Cu	CuN	X	1	14.600	7.188	75.190	0.00	0.00
ATOM	137	Cu	CuN	X	1	14.600	18.078	27.440	0.00	0.00

ATOM	138	Cu	CuN	X	1	14.600	18.078	43.360	0.00	0.00
ATOM	139	Cu	CuN	X	1	14.600	18.078	59.270	0.00	0.00
ATOM	140	Cu	CuN	X	1	14.600	18.078	75.190	0.00	0.00
ATOM	141	Cu	CuN	X	1	14.600	28.968	27.440	0.00	0.00
ATOM	142	Cu	CuN	X	1	14.600	28.968	43.360	0.00	0.00
ATOM	143	Cu	CuN	X	1	14.600	28.968	59.270	0.00	0.00
ATOM	144	Cu	CuN	X	1	14.600	28.968	75.190	0.00	0.00
ATOM	145	Cu	CuN	X	1	25.490	7.188	27.440	0.00	0.00
ATOM	146	Cu	CuN	X	1	25.490	7.188	43.360	0.00	0.00
ATOM	147	Cu	CuN	X	1	25.490	7.188	59.270	0.00	0.00
ATOM	148	Cu	CuN	X	1	25.490	7.188	75.190	0.00	0.00
ATOM	149	Cu	CuN	X	1	25.490	18.078	27.440	0.00	0.00
ATOM	150	Cu	CuN	X	1	25.490	18.078	43.360	0.00	0.00
ATOM	151	Cu	CuN	X	1	25.490	18.078	59.270	0.00	0.00
ATOM	152	Cu	CuN	X	1	25.490	18.078	75.190	0.00	0.00
ATOM	153	Cu	CuN	X	1	25.490	28.968	27.440	0.00	0.00
ATOM	154	Cu	CuN	X	1	25.490	28.968	43.360	0.00	0.00
ATOM	155	Cu	CuN	X	1	25.490	28.968	59.270	0.00	0.00
ATOM	156	Cu	CuN	X	1	25.490	28.968	75.190	0.00	0.00
ATOM	157	Cu	CuN	X	1	3.700	7.168	24.810	0.00	0.00
ATOM	158	Cu	CuN	X	1	3.700	7.168	40.720	0.00	0.00
ATOM	159	Cu	CuN	X	1	3.700	7.168	56.640	0.00	0.00
ATOM	160	Cu	CuN	X	1	3.700	7.168	72.550	0.00	0.00
ATOM	161	Cu	CuN	X	1	3.700	18.068	24.810	0.00	0.00
ATOM	162	Cu	CuN	X	1	3.700	18.068	40.720	0.00	0.00
ATOM	163	Cu	CuN	X	1	3.700	18.068	56.640	0.00	0.00
ATOM	164	Cu	CuN	X	1	3.700	18.068	72.550	0.00	0.00
ATOM	165	Cu	CuN	X	1	3.700	28.958	24.810	0.00	0.00
ATOM	166	Cu	CuN	X	1	3.700	28.958	40.720	0.00	0.00
ATOM	167	Cu	CuN	X	1	3.700	28.958	56.640	0.00	0.00

ATOM	168	Cu	CuN	X	1	3.700	28.958	72.550	0.00	0.00
ATOM	169	Cu	CuN	X	1	14.600	7.168	24.810	0.00	0.00
ATOM	170	Cu	CuN	X	1	14.600	7.168	40.720	0.00	0.00
ATOM	171	Cu	CuN	X	1	14.600	7.168	56.640	0.00	0.00
ATOM	172	Cu	CuN	X	1	14.600	7.168	72.550	0.00	0.00
ATOM	173	Cu	CuN	X	1	14.600	18.068	24.810	0.00	0.00
ATOM	174	Cu	CuN	X	1	14.600	18.068	40.720	0.00	0.00
ATOM	175	Cu	CuN	X	1	14.600	18.068	56.640	0.00	0.00
ATOM	176	Cu	CuN	X	1	14.600	18.068	72.550	0.00	0.00
ATOM	177	Cu	CuN	X	1	14.600	28.958	24.810	0.00	0.00
ATOM	178	Cu	CuN	X	1	14.600	28.958	40.720	0.00	0.00
ATOM	179	Cu	CuN	X	1	14.600	28.958	56.640	0.00	0.00
ATOM	180	Cu	CuN	X	1	14.600	28.958	72.550	0.00	0.00
ATOM	181	Cu	CuN	X	1	25.490	7.168	24.810	0.00	0.00
ATOM	182	Cu	CuN	X	1	25.490	7.168	40.720	0.00	0.00
ATOM	183	Cu	CuN	X	1	25.490	7.168	56.640	0.00	0.00
ATOM	184	Cu	CuN	X	1	25.490	7.168	72.550	0.00	0.00
ATOM	185	Cu	CuN	X	1	25.490	18.068	24.810	0.00	0.00
ATOM	186	Cu	CuN	X	1	25.490	18.068	40.720	0.00	0.00
ATOM	187	Cu	CuN	X	1	25.490	18.068	56.640	0.00	0.00
ATOM	188	Cu	CuN	X	1	25.490	18.068	72.550	0.00	0.00
ATOM	189	Cu	CuN	X	1	25.490	28.958	24.810	0.00	0.00
ATOM	190	Cu	CuN	X	1	25.490	28.958	40.720	0.00	0.00
ATOM	191	Cu	CuN	X	1	25.490	28.958	56.640	0.00	0.00
ATOM	192	Cu	CuN	X	1	25.490	28.958	72.550	0.00	0.00
ATOM	193	C1	CuN	X	1	12.060	7.168	26.120	0.00	0.00
ATOM	194	C1	CuN	X	1	12.060	7.168	42.040	0.00	0.00
ATOM	195	C1	CuN	X	1	12.060	7.168	57.950	0.00	0.00
ATOM	196	C1	CuN	X	1	12.060	7.168	73.870	0.00	0.00
ATOM	197	C1	CuN	X	1	12.060	18.058	26.120	0.00	0.00

ATOM	198	C1	CuN	X	1	12.060	18.058	42.040	0.00	0.00
ATOM	199	C1	CuN	X	1	12.060	18.058	57.950	0.00	0.00
ATOM	200	C1	CuN	X	1	12.060	18.058	73.870	0.00	0.00
ATOM	201	C1	CuN	X	1	12.060	28.948	26.120	0.00	0.00
ATOM	202	C1	CuN	X	1	12.060	28.948	42.040	0.00	0.00
ATOM	203	C1	CuN	X	1	12.060	28.948	57.950	0.00	0.00
ATOM	204	C1	CuN	X	1	12.060	28.948	73.870	0.00	0.00
ATOM	205	C1	CuN	X	1	22.950	7.168	26.120	0.00	0.00
ATOM	206	C1	CuN	X	1	22.950	7.168	42.040	0.00	0.00
ATOM	207	C1	CuN	X	1	22.950	7.168	57.950	0.00	0.00
ATOM	208	C1	CuN	X	1	22.950	7.168	73.870	0.00	0.00
ATOM	209	C1	CuN	X	1	22.950	18.058	26.120	0.00	0.00
ATOM	210	C1	CuN	X	1	22.950	18.058	42.040	0.00	0.00
ATOM	211	C1	CuN	X	1	22.950	18.058	57.950	0.00	0.00
ATOM	212	C1	CuN	X	1	22.950	18.058	73.870	0.00	0.00
ATOM	213	C1	CuN	X	1	22.950	28.948	26.120	0.00	0.00
ATOM	214	C1	CuN	X	1	22.950	28.948	42.040	0.00	0.00
ATOM	215	C1	CuN	X	1	22.950	28.948	57.950	0.00	0.00
ATOM	216	C1	CuN	X	1	22.950	28.948	73.870	0.00	0.00
ATOM	217	C1	CuN	X	1	1.170	7.168	26.120	0.00	0.00
ATOM	218	C1	CuN	X	1	1.170	7.168	42.040	0.00	0.00
ATOM	219	C1	CuN	X	1	1.170	7.168	57.950	0.00	0.00
ATOM	220	C1	CuN	X	1	1.170	7.168	73.870	0.00	0.00
ATOM	221	C1	CuN	X	1	1.170	18.058	26.120	0.00	0.00
ATOM	222	C1	CuN	X	1	1.170	18.058	42.040	0.00	0.00
ATOM	223	C1	CuN	X	1	1.170	18.058	57.950	0.00	0.00
ATOM	224	C1	CuN	X	1	1.170	18.058	73.870	0.00	0.00
ATOM	225	C1	CuN	X	1	1.170	28.948	26.120	0.00	0.00
ATOM	226	C1	CuN	X	1	1.170	28.948	42.040	0.00	0.00
ATOM	227	C1	CuN	X	1	1.170	28.948	57.950	0.00	0.00

ATOM	228	C1	CuN	X	1	1.170	28.948	73.870	0.00	0.00
ATOM	229	C2	CuN	X	1	10.560	7.158	26.130	0.00	0.00
ATOM	230	C2	CuN	X	1	10.560	7.158	42.040	0.00	0.00
ATOM	231	C2	CuN	X	1	10.560	7.158	57.950	0.00	0.00
ATOM	232	C2	CuN	X	1	10.560	7.158	73.870	0.00	0.00
ATOM	233	C2	CuN	X	1	10.560	18.048	26.130	0.00	0.00
ATOM	234	C2	CuN	X	1	10.560	18.048	42.040	0.00	0.00
ATOM	235	C2	CuN	X	1	10.560	18.048	57.950	0.00	0.00
ATOM	236	C2	CuN	X	1	10.560	18.048	73.870	0.00	0.00
ATOM	237	C2	CuN	X	1	10.560	28.938	26.130	0.00	0.00
ATOM	238	C2	CuN	X	1	10.560	28.938	42.040	0.00	0.00
ATOM	239	C2	CuN	X	1	10.560	28.938	57.950	0.00	0.00
ATOM	240	C2	CuN	X	1	10.560	28.938	73.870	0.00	0.00
ATOM	241	C2	CuN	X	1	21.450	7.158	26.130	0.00	0.00
ATOM	242	C2	CuN	X	1	21.450	7.158	42.040	0.00	0.00
ATOM	243	C2	CuN	X	1	21.450	7.158	57.950	0.00	0.00
ATOM	244	C2	CuN	X	1	21.450	7.158	73.870	0.00	0.00
ATOM	245	C2	CuN	X	1	21.450	18.048	26.130	0.00	0.00
ATOM	246	C2	CuN	X	1	21.450	18.048	42.040	0.00	0.00
ATOM	247	C2	CuN	X	1	21.450	18.048	57.950	0.00	0.00
ATOM	248	C2	CuN	X	1	21.450	18.048	73.870	0.00	0.00
ATOM	249	C2	CuN	X	1	21.450	28.938	26.130	0.00	0.00
ATOM	250	C2	CuN	X	1	21.450	28.938	42.040	0.00	0.00
ATOM	251	C2	CuN	X	1	21.450	28.938	57.950	0.00	0.00
ATOM	252	C2	CuN	X	1	21.450	28.938	73.870	0.00	0.00
ATOM	253	C2	CuN	X	1	32.340	7.158	26.130	0.00	0.00
ATOM	254	C2	CuN	X	1	32.340	7.158	42.040	0.00	0.00
ATOM	255	C2	CuN	X	1	32.340	7.158	57.950	0.00	0.00
ATOM	256	C2	CuN	X	1	32.340	7.158	73.870	0.00	0.00
ATOM	257	C2	CuN	X	1	32.340	18.048	26.130	0.00	0.00

```
ATOM    258  C2  CuN X   1      32.340  18.048  42.040  0.00  0.00
ATOM    259  C2  CuN X   1      32.340  18.048  57.950  0.00  0.00
ATOM    260  C2  CuN X   1      32.340  18.048  73.870  0.00  0.00
ATOM    261  C2  CuN X   1      32.340  28.938  26.130  0.00  0.00
ATOM    262  C2  CuN X   1      32.340  28.938  42.040  0.00  0.00
ATOM    263  C2  CuN X   1      32.340  28.938  57.950  0.00  0.00
ATOM    264  C2  CuN X   1      32.340  28.938  73.870  0.00  0.00
ATOM    265  C1  CuN X   1       6.240   7.178  26.120  0.00  0.00
ATOM    266  C1  CuN X   1       6.240   7.178  42.040  0.00  0.00
ATOM    267  C1  CuN X   1       6.240   7.178  57.950  0.00  0.00
ATOM    268  C1  CuN X   1       6.240   7.178  73.870  0.00  0.00
ATOM    269  C1  CuN X   1       6.240  18.068  26.120  0.00  0.00
ATOM    270  C1  CuN X   1       6.240  18.068  42.040  0.00  0.00
ATOM    271  C1  CuN X   1       6.240  18.068  57.950  0.00  0.00
ATOM    272  C1  CuN X   1       6.240  18.068  73.870  0.00  0.00
ATOM    273  C1  CuN X   1       6.240  28.958  26.120  0.00  0.00
ATOM    274  C1  CuN X   1       6.240  28.958  42.040  0.00  0.00
ATOM    275  C1  CuN X   1       6.240  28.958  57.950  0.00  0.00
ATOM    276  C1  CuN X   1       6.240  28.958  73.870  0.00  0.00
ATOM    277  C1  CuN X   1      17.130   7.178  26.120  0.00  0.00
ATOM    278  C1  CuN X   1      17.130   7.178  42.040  0.00  0.00
ATOM    279  C1  CuN X   1      17.130   7.178  57.950  0.00  0.00
ATOM    280  C1  CuN X   1      17.130   7.178  73.870  0.00  0.00
ATOM    281  C1  CuN X   1      17.130  18.068  26.120  0.00  0.00
ATOM    282  C1  CuN X   1      17.130  18.068  42.040  0.00  0.00
ATOM    283  C1  CuN X   1      17.130  18.068  57.950  0.00  0.00
ATOM    284  C1  CuN X   1      17.130  18.068  73.870  0.00  0.00
ATOM    285  C1  CuN X   1      17.130  28.958  26.120  0.00  0.00
ATOM    286  C1  CuN X   1      17.130  28.958  42.040  0.00  0.00
ATOM    287  C1  CuN X   1      17.130  28.958  57.950  0.00  0.00
```

```
ATOM    288  C1  CuN X   1      17.130  28.958  73.870  0.00  0.00
ATOM    289  C1  CuN X   1      28.030   7.178  26.120  0.00  0.00
ATOM    290  C1  CuN X   1      28.030   7.178  42.040  0.00  0.00
ATOM    291  C1  CuN X   1      28.030   7.178  57.950  0.00  0.00
ATOM    292  C1  CuN X   1      28.030   7.178  73.870  0.00  0.00
ATOM    293  C1  CuN X   1      28.030  18.068  26.120  0.00  0.00
ATOM    294  C1  CuN X   1      28.030  18.068  42.040  0.00  0.00
ATOM    295  C1  CuN X   1      28.030  18.068  57.950  0.00  0.00
ATOM    296  C1  CuN X   1      28.030  18.068  73.870  0.00  0.00
ATOM    297  C1  CuN X   1      28.030  28.958  26.120  0.00  0.00
ATOM    298  C1  CuN X   1      28.030  28.958  42.040  0.00  0.00
ATOM    299  C1  CuN X   1      28.030  28.958  57.950  0.00  0.00
ATOM    300  C1  CuN X   1      28.030  28.958  73.870  0.00  0.00
ATOM    301  C2  CuN X   1       7.750   7.168  26.130  0.00  0.00
ATOM    302  C2  CuN X   1       7.750   7.168  42.040  0.00  0.00
ATOM    303  C2  CuN X   1       7.750   7.168  57.950  0.00  0.00
ATOM    304  C2  CuN X   1       7.750   7.168  73.870  0.00  0.00
ATOM    305  C2  CuN X   1       7.750  18.058  26.130  0.00  0.00
ATOM    306  C2  CuN X   1       7.750  18.058  42.040  0.00  0.00
ATOM    307  C2  CuN X   1       7.750  18.058  57.950  0.00  0.00
ATOM    308  C2  CuN X   1       7.750  18.058  73.870  0.00  0.00
ATOM    309  C2  CuN X   1       7.750  28.948  26.130  0.00  0.00
ATOM    310  C2  CuN X   1       7.750  28.948  42.040  0.00  0.00
ATOM    311  C2  CuN X   1       7.750  28.948  57.950  0.00  0.00
ATOM    312  C2  CuN X   1       7.750  28.948  73.870  0.00  0.00
ATOM    313  C2  CuN X   1      18.640   7.168  26.130  0.00  0.00
ATOM    314  C2  CuN X   1      18.640   7.168  42.040  0.00  0.00
ATOM    315  C2  CuN X   1      18.640   7.168  57.950  0.00  0.00
ATOM    316  C2  CuN X   1      18.640   7.168  73.870  0.00  0.00
ATOM    317  C2  CuN X   1      18.640  18.058  26.130  0.00  0.00
```

ATOM	318	C2	CuN	X	1	18.640	18.058	42.040	0.00	0.00
ATOM	319	C2	CuN	X	1	18.640	18.058	57.950	0.00	0.00
ATOM	320	C2	CuN	X	1	18.640	18.058	73.870	0.00	0.00
ATOM	321	C2	CuN	X	1	18.640	28.948	26.130	0.00	0.00
ATOM	322	C2	CuN	X	1	18.640	28.948	42.040	0.00	0.00
ATOM	323	C2	CuN	X	1	18.640	28.948	57.950	0.00	0.00
ATOM	324	C2	CuN	X	1	18.640	28.948	73.870	0.00	0.00
ATOM	325	C2	CuN	X	1	29.530	7.168	26.130	0.00	0.00
ATOM	326	C2	CuN	X	1	29.530	7.168	42.040	0.00	0.00
ATOM	327	C2	CuN	X	1	29.530	7.168	57.950	0.00	0.00
ATOM	328	C2	CuN	X	1	29.530	7.168	73.870	0.00	0.00
ATOM	329	C2	CuN	X	1	29.530	18.058	26.130	0.00	0.00
ATOM	330	C2	CuN	X	1	29.530	18.058	42.040	0.00	0.00
ATOM	331	C2	CuN	X	1	29.530	18.058	57.950	0.00	0.00
ATOM	332	C2	CuN	X	1	29.530	18.058	73.870	0.00	0.00
ATOM	333	C2	CuN	X	1	29.530	28.948	26.130	0.00	0.00
ATOM	334	C2	CuN	X	1	29.530	28.948	42.040	0.00	0.00
ATOM	335	C2	CuN	X	1	29.530	28.948	57.950	0.00	0.00
ATOM	336	C2	CuN	X	1	29.530	28.948	73.870	0.00	0.00
ATOM	337	C2	CuN	X	1	9.860	7.148	24.900	0.00	0.00
ATOM	338	C2	CuN	X	1	9.860	7.148	40.810	0.00	0.00
ATOM	339	C2	CuN	X	1	9.860	7.148	56.730	0.00	0.00
ATOM	340	C2	CuN	X	1	9.860	7.148	72.640	0.00	0.00
ATOM	341	C2	CuN	X	1	9.860	18.038	24.900	0.00	0.00
ATOM	342	C2	CuN	X	1	9.860	18.038	40.810	0.00	0.00
ATOM	343	C2	CuN	X	1	9.860	18.038	56.730	0.00	0.00
ATOM	344	C2	CuN	X	1	9.860	18.038	72.640	0.00	0.00
ATOM	345	C2	CuN	X	1	9.860	28.928	24.900	0.00	0.00
ATOM	346	C2	CuN	X	1	9.860	28.928	40.810	0.00	0.00
ATOM	347	C2	CuN	X	1	9.860	28.928	56.730	0.00	0.00

ATOM 348 C2 CuN X 1 9.860 28.928 72.640 0.00 0.00
ATOM 349 C2 CuN X 1 20.750 7.148 24.900 0.00 0.00
ATOM 350 C2 CuN X 1 20.750 7.148 40.810 0.00 0.00
ATOM 351 C2 CuN X 1 20.750 7.148 56.730 0.00 0.00
ATOM 352 C2 CuN X 1 20.750 7.148 72.640 0.00 0.00
ATOM 353 C2 CuN X 1 20.750 18.038 24.900 0.00 0.00
ATOM 354 C2 CuN X 1 20.750 18.038 40.810 0.00 0.00
ATOM 355 C2 CuN X 1 20.750 18.038 56.730 0.00 0.00
ATOM 356 C2 CuN X 1 20.750 18.038 72.640 0.00 0.00
ATOM 357 C2 CuN X 1 20.750 28.928 24.900 0.00 0.00
ATOM 358 C2 CuN X 1 20.750 28.928 40.810 0.00 0.00
ATOM 359 C2 CuN X 1 20.750 28.928 56.730 0.00 0.00
ATOM 360 C2 CuN X 1 20.750 28.928 72.640 0.00 0.00
ATOM 361 C2 CuN X 1 31.640 7.148 24.900 0.00 0.00
ATOM 362 C2 CuN X 1 31.640 7.148 40.810 0.00 0.00
ATOM 363 C2 CuN X 1 31.640 7.148 56.730 0.00 0.00
ATOM 364 C2 CuN X 1 31.640 7.148 72.640 0.00 0.00
ATOM 365 C2 CuN X 1 31.640 18.038 24.900 0.00 0.00
ATOM 366 C2 CuN X 1 31.640 18.038 40.810 0.00 0.00
ATOM 367 C2 CuN X 1 31.640 18.038 56.730 0.00 0.00
ATOM 368 C2 CuN X 1 31.640 18.038 72.640 0.00 0.00
ATOM 369 C2 CuN X 1 31.640 28.928 24.900 0.00 0.00
ATOM 370 C2 CuN X 1 31.640 28.928 40.810 0.00 0.00
ATOM 371 C2 CuN X 1 31.640 28.928 56.730 0.00 0.00
ATOM 372 C2 CuN X 1 31.640 28.928 72.640 0.00 0.00
ATOM 373 C2 CuN X 1 8.450 7.128 24.900 0.00 0.00
ATOM 374 C2 CuN X 1 8.450 7.128 40.810 0.00 0.00
ATOM 375 C2 CuN X 1 8.450 7.128 56.730 0.00 0.00
ATOM 376 C2 CuN X 1 8.450 7.128 72.640 0.00 0.00
ATOM 377 C2 CuN X 1 8.450 18.018 24.900 0.00 0.00

ATOM	378	C2	CuN	X	1	8.450	18.018	40.810	0.00	0.00
ATOM	379	C2	CuN	X	1	8.450	18.018	56.730	0.00	0.00
ATOM	380	C2	CuN	X	1	8.450	18.018	72.640	0.00	0.00
ATOM	381	C2	CuN	X	1	8.450	28.908	24.900	0.00	0.00
ATOM	382	C2	CuN	X	1	8.450	28.908	40.810	0.00	0.00
ATOM	383	C2	CuN	X	1	8.450	28.908	56.730	0.00	0.00
ATOM	384	C2	CuN	X	1	8.450	28.908	72.640	0.00	0.00
ATOM	385	C2	CuN	X	1	19.340	7.128	24.900	0.00	0.00
ATOM	386	C2	CuN	X	1	19.340	7.128	40.810	0.00	0.00
ATOM	387	C2	CuN	X	1	19.340	7.128	56.730	0.00	0.00
ATOM	388	C2	CuN	X	1	19.340	7.128	72.640	0.00	0.00
ATOM	389	C2	CuN	X	1	19.340	18.018	24.900	0.00	0.00
ATOM	390	C2	CuN	X	1	19.340	18.018	40.810	0.00	0.00
ATOM	391	C2	CuN	X	1	19.340	18.018	56.730	0.00	0.00
ATOM	392	C2	CuN	X	1	19.340	18.018	72.640	0.00	0.00
ATOM	393	C2	CuN	X	1	19.340	28.908	24.900	0.00	0.00
ATOM	394	C2	CuN	X	1	19.340	28.908	40.810	0.00	0.00
ATOM	395	C2	CuN	X	1	19.340	28.908	56.730	0.00	0.00
ATOM	396	C2	CuN	X	1	19.340	28.908	72.640	0.00	0.00
ATOM	397	C2	CuN	X	1	30.230	7.128	24.900	0.00	0.00
ATOM	398	C2	CuN	X	1	30.230	7.128	40.810	0.00	0.00
ATOM	399	C2	CuN	X	1	30.230	7.128	56.730	0.00	0.00
ATOM	400	C2	CuN	X	1	30.230	7.128	72.640	0.00	0.00
ATOM	401	C2	CuN	X	1	30.230	18.018	24.900	0.00	0.00
ATOM	402	C2	CuN	X	1	30.230	18.018	40.810	0.00	0.00
ATOM	403	C2	CuN	X	1	30.230	18.018	56.730	0.00	0.00
ATOM	404	C2	CuN	X	1	30.230	18.018	72.640	0.00	0.00
ATOM	405	C2	CuN	X	1	30.230	28.908	24.900	0.00	0.00
ATOM	406	C2	CuN	X	1	30.230	28.908	40.810	0.00	0.00
ATOM	407	C2	CuN	X	1	30.230	28.908	56.730	0.00	0.00

ATOM	408	C2	CuN	X	1	30.230	28.908	72.640	0.00	0.00
ATOM	409	C2	CuN	X	1	8.450	7.188	27.350	0.00	0.00
ATOM	410	C2	CuN	X	1	8.450	7.188	43.270	0.00	0.00
ATOM	411	C2	CuN	X	1	8.450	7.188	59.180	0.00	0.00
ATOM	412	C2	CuN	X	1	8.450	7.188	75.100	0.00	0.00
ATOM	413	C2	CuN	X	1	8.450	18.078	27.350	0.00	0.00
ATOM	414	C2	CuN	X	1	8.450	18.078	43.270	0.00	0.00
ATOM	415	C2	CuN	X	1	8.450	18.078	59.180	0.00	0.00
ATOM	416	C2	CuN	X	1	8.450	18.078	75.100	0.00	0.00
ATOM	417	C2	CuN	X	1	8.450	28.978	27.350	0.00	0.00
ATOM	418	C2	CuN	X	1	8.450	28.978	43.270	0.00	0.00
ATOM	419	C2	CuN	X	1	8.450	28.978	59.180	0.00	0.00
ATOM	420	C2	CuN	X	1	8.450	28.978	75.100	0.00	0.00
ATOM	421	C2	CuN	X	1	19.340	7.188	27.350	0.00	0.00
ATOM	422	C2	CuN	X	1	19.340	7.188	43.270	0.00	0.00
ATOM	423	C2	CuN	X	1	19.340	7.188	59.180	0.00	0.00
ATOM	424	C2	CuN	X	1	19.340	7.188	75.100	0.00	0.00
ATOM	425	C2	CuN	X	1	19.340	18.078	27.350	0.00	0.00
ATOM	426	C2	CuN	X	1	19.340	18.078	43.270	0.00	0.00
ATOM	427	C2	CuN	X	1	19.340	18.078	59.180	0.00	0.00
ATOM	428	C2	CuN	X	1	19.340	18.078	75.100	0.00	0.00
ATOM	429	C2	CuN	X	1	19.340	28.978	27.350	0.00	0.00
ATOM	430	C2	CuN	X	1	19.340	28.978	43.270	0.00	0.00
ATOM	431	C2	CuN	X	1	19.340	28.978	59.180	0.00	0.00
ATOM	432	C2	CuN	X	1	19.340	28.978	75.100	0.00	0.00
ATOM	433	C2	CuN	X	1	30.230	7.188	27.350	0.00	0.00
ATOM	434	C2	CuN	X	1	30.230	7.188	43.270	0.00	0.00
ATOM	435	C2	CuN	X	1	30.230	7.188	59.180	0.00	0.00
ATOM	436	C2	CuN	X	1	30.230	7.188	75.100	0.00	0.00
ATOM	437	C2	CuN	X	1	30.230	18.078	27.350	0.00	0.00

ATOM	438	C2	CuN	X	1	30.230	18.078	43.270	0.00	0.00
ATOM	439	C2	CuN	X	1	30.230	18.078	59.180	0.00	0.00
ATOM	440	C2	CuN	X	1	30.230	18.078	75.100	0.00	0.00
ATOM	441	C2	CuN	X	1	30.230	28.978	27.350	0.00	0.00
ATOM	442	C2	CuN	X	1	30.230	28.978	43.270	0.00	0.00
ATOM	443	C2	CuN	X	1	30.230	28.978	59.180	0.00	0.00
ATOM	444	C2	CuN	X	1	30.230	28.978	75.100	0.00	0.00
ATOM	445	C2	CuN	X	1	9.860	7.168	27.350	0.00	0.00
ATOM	446	C2	CuN	X	1	9.860	7.168	43.270	0.00	0.00
ATOM	447	C2	CuN	X	1	9.860	7.168	59.180	0.00	0.00
ATOM	448	C2	CuN	X	1	9.860	7.168	75.100	0.00	0.00
ATOM	449	C2	CuN	X	1	9.860	18.058	27.350	0.00	0.00
ATOM	450	C2	CuN	X	1	9.860	18.058	43.270	0.00	0.00
ATOM	451	C2	CuN	X	1	9.860	18.058	59.180	0.00	0.00
ATOM	452	C2	CuN	X	1	9.860	18.058	75.100	0.00	0.00
ATOM	453	C2	CuN	X	1	9.860	28.958	27.350	0.00	0.00
ATOM	454	C2	CuN	X	1	9.860	28.958	43.270	0.00	0.00
ATOM	455	C2	CuN	X	1	9.860	28.958	59.180	0.00	0.00
ATOM	456	C2	CuN	X	1	9.860	28.958	75.100	0.00	0.00
ATOM	457	C2	CuN	X	1	20.750	7.168	27.350	0.00	0.00
ATOM	458	C2	CuN	X	1	20.750	7.168	43.270	0.00	0.00
ATOM	459	C2	CuN	X	1	20.750	7.168	59.180	0.00	0.00
ATOM	460	C2	CuN	X	1	20.750	7.168	75.100	0.00	0.00
ATOM	461	C2	CuN	X	1	20.750	18.058	27.350	0.00	0.00
ATOM	462	C2	CuN	X	1	20.750	18.058	43.270	0.00	0.00
ATOM	463	C2	CuN	X	1	20.750	18.058	59.180	0.00	0.00
ATOM	464	C2	CuN	X	1	20.750	18.058	75.100	0.00	0.00
ATOM	465	C2	CuN	X	1	20.750	28.958	27.350	0.00	0.00
ATOM	466	C2	CuN	X	1	20.750	28.958	43.270	0.00	0.00
ATOM	467	C2	CuN	X	1	20.750	28.958	59.180	0.00	0.00

```
ATOM    468  C2  CuN X   1      20.750  28.958  75.100  0.00  0.00
ATOM    469  C2  CuN X   1      31.640   7.168  27.350  0.00  0.00
ATOM    470  C2  CuN X   1      31.640   7.168  43.270  0.00  0.00
ATOM    471  C2  CuN X   1      31.640   7.168  59.180  0.00  0.00
ATOM    472  C2  CuN X   1      31.640   7.168  75.100  0.00  0.00
ATOM    473  C2  CuN X   1      31.640  18.058  27.350  0.00  0.00
ATOM    474  C2  CuN X   1      31.640  18.058  43.270  0.00  0.00
ATOM    475  C2  CuN X   1      31.640  18.058  59.180  0.00  0.00
ATOM    476  C2  CuN X   1      31.640  18.058  75.100  0.00  0.00
ATOM    477  C2  CuN X   1      31.640  28.958  27.350  0.00  0.00
ATOM    478  C2  CuN X   1      31.640  28.958  43.270  0.00  0.00
ATOM    479  C2  CuN X   1      31.640  28.958  59.180  0.00  0.00
ATOM    480  C2  CuN X   1      31.640  28.958  75.100  0.00  0.00
ATOM    481  N1  CuN X   1       3.680   7.308  29.540  0.00  0.00
ATOM    482  N1  CuN X   1       3.680   7.308  45.460  0.00  0.00
ATOM    483  N1  CuN X   1       3.680   7.308  61.370  0.00  0.00
ATOM    484  N1  CuN X   1       3.680  18.208  29.540  0.00  0.00
ATOM    485  N1  CuN X   1       3.680  18.208  45.460  0.00  0.00
ATOM    486  N1  CuN X   1       3.680  18.208  61.370  0.00  0.00
ATOM    487  N1  CuN X   1       3.680  29.098  29.540  0.00  0.00
ATOM    488  N1  CuN X   1       3.680  29.098  45.460  0.00  0.00
ATOM    489  N1  CuN X   1       3.680  29.098  61.370  0.00  0.00
ATOM    490  N1  CuN X   1      14.570   7.308  29.540  0.00  0.00
ATOM    491  N1  CuN X   1      14.570   7.308  45.460  0.00  0.00
ATOM    492  N1  CuN X   1      14.570   7.308  61.370  0.00  0.00
ATOM    493  N1  CuN X   1      14.570  18.208  29.540  0.00  0.00
ATOM    494  N1  CuN X   1      14.570  18.208  45.460  0.00  0.00
ATOM    495  N1  CuN X   1      14.570  18.208  61.370  0.00  0.00
ATOM    496  N1  CuN X   1      14.570  29.098  29.540  0.00  0.00
ATOM    497  N1  CuN X   1      14.570  29.098  45.460  0.00  0.00
```

ATOM	498	N1	CuN	X	1	14.570	29.098	61.370	0.00	0.00
ATOM	499	N1	CuN	X	1	25.470	7.308	29.540	0.00	0.00
ATOM	500	N1	CuN	X	1	25.470	7.308	45.460	0.00	0.00
ATOM	501	N1	CuN	X	1	25.470	7.308	61.370	0.00	0.00
ATOM	502	N1	CuN	X	1	25.470	18.208	29.540	0.00	0.00
ATOM	503	N1	CuN	X	1	25.470	18.208	45.460	0.00	0.00
ATOM	504	N1	CuN	X	1	25.470	18.208	61.370	0.00	0.00
ATOM	505	N1	CuN	X	1	25.470	29.098	29.540	0.00	0.00
ATOM	506	N1	CuN	X	1	25.470	29.098	45.460	0.00	0.00
ATOM	507	N1	CuN	X	1	25.470	29.098	61.370	0.00	0.00
ATOM	508	C3	CuN	X	1	3.930	6.218	30.310	0.00	0.00
ATOM	509	C3	CuN	X	1	3.930	6.218	46.220	0.00	0.00
ATOM	510	C3	CuN	X	1	3.930	6.218	62.140	0.00	0.00
ATOM	511	C3	CuN	X	1	3.930	17.108	30.310	0.00	0.00
ATOM	512	C3	CuN	X	1	3.930	17.108	46.220	0.00	0.00
ATOM	513	C3	CuN	X	1	3.930	17.108	62.140	0.00	0.00
ATOM	514	C3	CuN	X	1	3.930	27.998	30.310	0.00	0.00
ATOM	515	C3	CuN	X	1	3.930	27.998	46.220	0.00	0.00
ATOM	516	C3	CuN	X	1	3.930	27.998	62.140	0.00	0.00
ATOM	517	C3	CuN	X	1	14.830	6.218	30.310	0.00	0.00
ATOM	518	C3	CuN	X	1	14.830	6.218	46.220	0.00	0.00
ATOM	519	C3	CuN	X	1	14.830	6.218	62.140	0.00	0.00
ATOM	520	C3	CuN	X	1	14.830	17.108	30.310	0.00	0.00
ATOM	521	C3	CuN	X	1	14.830	17.108	46.220	0.00	0.00
ATOM	522	C3	CuN	X	1	14.830	17.108	62.140	0.00	0.00
ATOM	523	C3	CuN	X	1	14.830	27.998	30.310	0.00	0.00
ATOM	524	C3	CuN	X	1	14.830	27.998	46.220	0.00	0.00
ATOM	525	C3	CuN	X	1	14.830	27.998	62.140	0.00	0.00
ATOM	526	C3	CuN	X	1	25.720	6.218	30.310	0.00	0.00
ATOM	527	C3	CuN	X	1	25.720	6.218	46.220	0.00	0.00

ATOM 528 C3 CuN X 1 25.720 6.218 62.140 0.00 0.00

ATOM 529 C3 CuN X 1 25.720 17.108 30.310 0.00 0.00

ATOM 530 C3 CuN X 1 25.720 17.108 46.220 0.00 0.00

ATOM 531 C3 CuN X 1 25.720 17.108 62.140 0.00 0.00

ATOM 532 C3 CuN X 1 25.720 27.998 30.310 0.00 0.00

ATOM 533 C3 CuN X 1 25.720 27.998 46.220 0.00 0.00

ATOM 534 C3 CuN X 1 25.720 27.998 62.140 0.00 0.00

ATOM 535 C4 CuN X 1 3.960 6.258 31.710 0.00 0.00

ATOM 536 C4 CuN X 1 3.960 6.258 47.620 0.00 0.00

ATOM 537 C4 CuN X 1 3.960 6.258 63.540 0.00 0.00

ATOM 538 C4 CuN X 1 3.960 17.148 31.710 0.00 0.00

ATOM 539 C4 CuN X 1 3.960 17.148 47.620 0.00 0.00

ATOM 540 C4 CuN X 1 3.960 17.148 63.540 0.00 0.00

ATOM 541 C4 CuN X 1 3.960 28.048 31.710 0.00 0.00

ATOM 542 C4 CuN X 1 3.960 28.048 47.620 0.00 0.00

ATOM 543 C4 CuN X 1 3.960 28.048 63.540 0.00 0.00

ATOM 544 C4 CuN X 1 14.850 6.258 31.710 0.00 0.00

ATOM 545 C4 CuN X 1 14.850 6.258 47.620 0.00 0.00

ATOM 546 C4 CuN X 1 14.850 6.258 63.540 0.00 0.00

ATOM 547 C4 CuN X 1 14.850 17.148 31.710 0.00 0.00

ATOM 548 C4 CuN X 1 14.850 17.148 47.620 0.00 0.00

ATOM 549 C4 CuN X 1 14.850 17.148 63.540 0.00 0.00

ATOM 550 C4 CuN X 1 14.850 28.048 31.710 0.00 0.00

ATOM 551 C4 CuN X 1 14.850 28.048 47.620 0.00 0.00

ATOM 552 C4 CuN X 1 14.850 28.048 63.540 0.00 0.00

ATOM 553 C4 CuN X 1 25.750 6.258 31.710 0.00 0.00

ATOM 554 C4 CuN X 1 25.750 6.258 47.620 0.00 0.00

ATOM 555 C4 CuN X 1 25.750 6.258 63.540 0.00 0.00

ATOM 556 C4 CuN X 1 25.750 17.148 31.710 0.00 0.00

ATOM 557 C4 CuN X 1 25.750 17.148 47.620 0.00 0.00

ATOM	558	C4	CuN	X	1	25.750	17.148	63.540	0.00	0.00
ATOM	559	C4	CuN	X	1	25.750	28.048	31.710	0.00	0.00
ATOM	560	C4	CuN	X	1	25.750	28.048	47.620	0.00	0.00
ATOM	561	C4	CuN	X	1	25.750	28.048	63.540	0.00	0.00
ATOM	562	C5	CuN	X	1	3.710	7.508	32.340	0.00	0.00
ATOM	563	C5	CuN	X	1	3.710	7.508	48.260	0.00	0.00
ATOM	564	C5	CuN	X	1	3.710	7.508	64.170	0.00	0.00
ATOM	565	C5	CuN	X	1	3.710	18.398	32.340	0.00	0.00
ATOM	566	C5	CuN	X	1	3.710	18.398	48.260	0.00	0.00
ATOM	567	C5	CuN	X	1	3.710	18.398	64.170	0.00	0.00
ATOM	568	C5	CuN	X	1	3.710	29.288	32.340	0.00	0.00
ATOM	569	C5	CuN	X	1	3.710	29.288	48.260	0.00	0.00
ATOM	570	C5	CuN	X	1	3.710	29.288	64.170	0.00	0.00
ATOM	571	C5	CuN	X	1	14.610	7.508	32.340	0.00	0.00
ATOM	572	C5	CuN	X	1	14.610	7.508	48.260	0.00	0.00
ATOM	573	C5	CuN	X	1	14.610	7.508	64.170	0.00	0.00
ATOM	574	C5	CuN	X	1	14.610	18.398	32.340	0.00	0.00
ATOM	575	C5	CuN	X	1	14.610	18.398	48.260	0.00	0.00
ATOM	576	C5	CuN	X	1	14.610	18.398	64.170	0.00	0.00
ATOM	577	C5	CuN	X	1	14.610	29.288	32.340	0.00	0.00
ATOM	578	C5	CuN	X	1	14.610	29.288	48.260	0.00	0.00
ATOM	579	C5	CuN	X	1	14.610	29.288	64.170	0.00	0.00
ATOM	580	C5	CuN	X	1	25.500	7.508	32.340	0.00	0.00
ATOM	581	C5	CuN	X	1	25.500	7.508	48.260	0.00	0.00
ATOM	582	C5	CuN	X	1	25.500	7.508	64.170	0.00	0.00
ATOM	583	C5	CuN	X	1	25.500	18.398	32.340	0.00	0.00
ATOM	584	C5	CuN	X	1	25.500	18.398	48.260	0.00	0.00
ATOM	585	C5	CuN	X	1	25.500	18.398	64.170	0.00	0.00
ATOM	586	C5	CuN	X	1	25.500	29.288	32.340	0.00	0.00
ATOM	587	C5	CuN	X	1	25.500	29.288	48.260	0.00	0.00

```
ATOM    588  C5  CuN X   1      25.500  29.288  64.170  0.00  0.00
ATOM    589  C4  CuN X   1       3.460   8.648  31.550  0.00  0.00
ATOM    590  C4  CuN X   1       3.460   8.648  47.460  0.00  0.00
ATOM    591  C4  CuN X   1       3.460   8.648  63.370  0.00  0.00
ATOM    592  C4  CuN X   1       3.460  19.538  31.550  0.00  0.00
ATOM    593  C4  CuN X   1       3.460  19.538  47.460  0.00  0.00
ATOM    594  C4  CuN X   1       3.460  19.538  63.370  0.00  0.00
ATOM    595  C4  CuN X   1       3.460  30.428  31.550  0.00  0.00
ATOM    596  C4  CuN X   1       3.460  30.428  47.460  0.00  0.00
ATOM    597  C4  CuN X   1       3.460  30.428  63.370  0.00  0.00
ATOM    598  C4  CuN X   1      14.350   8.648  31.550  0.00  0.00
ATOM    599  C4  CuN X   1      14.350   8.648  47.460  0.00  0.00
ATOM    600  C4  CuN X   1      14.350   8.648  63.370  0.00  0.00
ATOM    601  C4  CuN X   1      14.350  19.538  31.550  0.00  0.00
ATOM    602  C4  CuN X   1      14.350  19.538  47.460  0.00  0.00
ATOM    603  C4  CuN X   1      14.350  19.538  63.370  0.00  0.00
ATOM    604  C4  CuN X   1      14.350  30.428  31.550  0.00  0.00
ATOM    605  C4  CuN X   1      14.350  30.428  47.460  0.00  0.00
ATOM    606  C4  CuN X   1      14.350  30.428  63.370  0.00  0.00
ATOM    607  C4  CuN X   1      25.250   8.648  31.550  0.00  0.00
ATOM    608  C4  CuN X   1      25.250   8.648  47.460  0.00  0.00
ATOM    609  C4  CuN X   1      25.250   8.648  63.370  0.00  0.00
ATOM    610  C4  CuN X   1      25.250  19.538  31.550  0.00  0.00
ATOM    611  C4  CuN X   1      25.250  19.538  47.460  0.00  0.00
ATOM    612  C4  CuN X   1      25.250  19.538  63.370  0.00  0.00
ATOM    613  C4  CuN X   1      25.250  30.428  31.550  0.00  0.00
ATOM    614  C4  CuN X   1      25.250  30.428  47.460  0.00  0.00
ATOM    615  C4  CuN X   1      25.250  30.428  63.370  0.00  0.00
ATOM    616  C3  CuN X   1       3.460   8.498  30.150  0.00  0.00
ATOM    617  C3  CuN X   1       3.460   8.498  46.060  0.00  0.00
```

ATOM	618	C3	CuN	X	1	3.460	8.498	61.980	0.00	0.00
ATOM	619	C3	CuN	X	1	3.460	19.398	30.150	0.00	0.00
ATOM	620	C3	CuN	X	1	3.460	19.398	46.060	0.00	0.00
ATOM	621	C3	CuN	X	1	3.460	19.398	61.980	0.00	0.00
ATOM	622	C3	CuN	X	1	3.460	30.288	30.150	0.00	0.00
ATOM	623	C3	CuN	X	1	3.460	30.288	46.060	0.00	0.00
ATOM	624	C3	CuN	X	1	3.460	30.288	61.980	0.00	0.00
ATOM	625	C3	CuN	X	1	14.350	8.498	30.150	0.00	0.00
ATOM	626	C3	CuN	X	1	14.350	8.498	46.060	0.00	0.00
ATOM	627	C3	CuN	X	1	14.350	8.498	61.980	0.00	0.00
ATOM	628	C3	CuN	X	1	14.350	19.398	30.150	0.00	0.00
ATOM	629	C3	CuN	X	1	14.350	19.398	46.060	0.00	0.00
ATOM	630	C3	CuN	X	1	14.350	19.398	61.980	0.00	0.00
ATOM	631	C3	CuN	X	1	14.350	30.288	30.150	0.00	0.00
ATOM	632	C3	CuN	X	1	14.350	30.288	46.060	0.00	0.00
ATOM	633	C3	CuN	X	1	14.350	30.288	61.980	0.00	0.00
ATOM	634	C3	CuN	X	1	25.240	8.498	30.150	0.00	0.00
ATOM	635	C3	CuN	X	1	25.240	8.498	46.060	0.00	0.00
ATOM	636	C3	CuN	X	1	25.240	8.498	61.980	0.00	0.00
ATOM	637	C3	CuN	X	1	25.240	19.398	30.150	0.00	0.00
ATOM	638	C3	CuN	X	1	25.240	19.398	46.060	0.00	0.00
ATOM	639	C3	CuN	X	1	25.240	19.398	61.980	0.00	0.00
ATOM	640	C3	CuN	X	1	25.240	30.288	30.150	0.00	0.00
ATOM	641	C3	CuN	X	1	25.240	30.288	46.060	0.00	0.00
ATOM	642	C3	CuN	X	1	25.240	30.288	61.980	0.00	0.00
ATOM	643	N2	CuN	X	1	3.670	7.718	33.750	0.00	0.00
ATOM	644	N2	CuN	X	1	3.670	7.718	49.660	0.00	0.00
ATOM	645	N2	CuN	X	1	3.670	7.718	65.580	0.00	0.00
ATOM	646	N2	CuN	X	1	3.670	18.608	33.750	0.00	0.00
ATOM	647	N2	CuN	X	1	3.670	18.608	49.660	0.00	0.00

ATOM	648	N2	CuN	X	1	3.670	18.608	65.580	0.00	0.00
ATOM	649	N2	CuN	X	1	3.670	29.498	33.750	0.00	0.00
ATOM	650	N2	CuN	X	1	3.670	29.498	49.660	0.00	0.00
ATOM	651	N2	CuN	X	1	3.670	29.498	65.580	0.00	0.00
ATOM	652	N2	CuN	X	1	14.560	7.718	33.750	0.00	0.00
ATOM	653	N2	CuN	X	1	14.560	7.718	49.660	0.00	0.00
ATOM	654	N2	CuN	X	1	14.560	7.718	65.580	0.00	0.00
ATOM	655	N2	CuN	X	1	14.560	18.608	33.750	0.00	0.00
ATOM	656	N2	CuN	X	1	14.560	18.608	49.660	0.00	0.00
ATOM	657	N2	CuN	X	1	14.560	18.608	65.580	0.00	0.00
ATOM	658	N2	CuN	X	1	14.560	29.498	33.750	0.00	0.00
ATOM	659	N2	CuN	X	1	14.560	29.498	49.660	0.00	0.00
ATOM	660	N2	CuN	X	1	14.560	29.498	65.580	0.00	0.00
ATOM	661	N2	CuN	X	1	25.460	7.718	33.750	0.00	0.00
ATOM	662	N2	CuN	X	1	25.460	7.718	49.660	0.00	0.00
ATOM	663	N2	CuN	X	1	25.460	7.718	65.580	0.00	0.00
ATOM	664	N2	CuN	X	1	25.460	18.608	33.750	0.00	0.00
ATOM	665	N2	CuN	X	1	25.460	18.608	49.660	0.00	0.00
ATOM	666	N2	CuN	X	1	25.460	18.608	65.580	0.00	0.00
ATOM	667	N2	CuN	X	1	25.460	29.498	33.750	0.00	0.00
ATOM	668	N2	CuN	X	1	25.460	29.498	49.660	0.00	0.00
ATOM	669	N2	CuN	X	1	25.460	29.498	65.580	0.00	0.00
ATOM	670	N2	CuN	X	1	3.970	6.678	34.430	0.00	0.00
ATOM	671	N2	CuN	X	1	3.970	6.678	50.340	0.00	0.00
ATOM	672	N2	CuN	X	1	3.970	6.678	66.250	0.00	0.00
ATOM	673	N2	CuN	X	1	3.970	17.568	34.430	0.00	0.00
ATOM	674	N2	CuN	X	1	3.970	17.568	50.340	0.00	0.00
ATOM	675	N2	CuN	X	1	3.970	17.568	66.250	0.00	0.00
ATOM	676	N2	CuN	X	1	3.970	28.458	34.430	0.00	0.00
ATOM	677	N2	CuN	X	1	3.970	28.458	50.340	0.00	0.00

ATOM	678	N2	CuN	X	1	3.970	28.458	66.250	0.00	0.00
ATOM	679	N2	CuN	X	1	14.860	6.678	34.430	0.00	0.00
ATOM	680	N2	CuN	X	1	14.860	6.678	50.340	0.00	0.00
ATOM	681	N2	CuN	X	1	14.860	6.678	66.250	0.00	0.00
ATOM	682	N2	CuN	X	1	14.860	17.568	34.430	0.00	0.00
ATOM	683	N2	CuN	X	1	14.860	17.568	50.340	0.00	0.00
ATOM	684	N2	CuN	X	1	14.860	17.568	66.250	0.00	0.00
ATOM	685	N2	CuN	X	1	14.860	28.458	34.430	0.00	0.00
ATOM	686	N2	CuN	X	1	14.860	28.458	50.340	0.00	0.00
ATOM	687	N2	CuN	X	1	14.860	28.458	66.250	0.00	0.00
ATOM	688	N2	CuN	X	1	25.750	6.678	34.430	0.00	0.00
ATOM	689	N2	CuN	X	1	25.750	6.678	50.340	0.00	0.00
ATOM	690	N2	CuN	X	1	25.750	6.678	66.250	0.00	0.00
ATOM	691	N2	CuN	X	1	25.750	17.568	34.430	0.00	0.00
ATOM	692	N2	CuN	X	1	25.750	17.568	50.340	0.00	0.00
ATOM	693	N2	CuN	X	1	25.750	17.568	66.250	0.00	0.00
ATOM	694	N2	CuN	X	1	25.750	28.458	34.430	0.00	0.00
ATOM	695	N2	CuN	X	1	25.750	28.458	50.340	0.00	0.00
ATOM	696	N2	CuN	X	1	25.750	28.458	66.250	0.00	0.00
ATOM	697	C5	CuN	X	1	3.870	6.868	35.830	0.00	0.00
ATOM	698	C5	CuN	X	1	3.870	6.868	51.750	0.00	0.00
ATOM	699	C5	CuN	X	1	3.870	6.868	67.660	0.00	0.00
ATOM	700	C5	CuN	X	1	3.870	17.768	35.830	0.00	0.00
ATOM	701	C5	CuN	X	1	3.870	17.768	51.750	0.00	0.00
ATOM	702	C5	CuN	X	1	3.870	17.768	67.660	0.00	0.00
ATOM	703	C5	CuN	X	1	3.870	28.658	35.830	0.00	0.00
ATOM	704	C5	CuN	X	1	3.870	28.658	51.750	0.00	0.00
ATOM	705	C5	CuN	X	1	3.870	28.658	67.660	0.00	0.00
ATOM	706	C5	CuN	X	1	14.770	6.868	35.830	0.00	0.00
ATOM	707	C5	CuN	X	1	14.770	6.868	51.750	0.00	0.00

ATOM	708	C5	CuN	X	1	14.770	6.868	67.660	0.00	0.00
ATOM	709	C5	CuN	X	1	14.770	17.768	35.830	0.00	0.00
ATOM	710	C5	CuN	X	1	14.770	17.768	51.750	0.00	0.00
ATOM	711	C5	CuN	X	1	14.770	17.768	67.660	0.00	0.00
ATOM	712	C5	CuN	X	1	14.770	28.658	35.830	0.00	0.00
ATOM	713	C5	CuN	X	1	14.770	28.658	51.750	0.00	0.00
ATOM	714	C5	CuN	X	1	14.770	28.658	67.660	0.00	0.00
ATOM	715	C5	CuN	X	1	25.660	6.868	35.830	0.00	0.00
ATOM	716	C5	CuN	X	1	25.660	6.868	51.750	0.00	0.00
ATOM	717	C5	CuN	X	1	25.660	6.868	67.660	0.00	0.00
ATOM	718	C5	CuN	X	1	25.660	17.768	35.830	0.00	0.00
ATOM	719	C5	CuN	X	1	25.660	17.768	51.750	0.00	0.00
ATOM	720	C5	CuN	X	1	25.660	17.768	67.660	0.00	0.00
ATOM	721	C5	CuN	X	1	25.660	28.658	35.830	0.00	0.00
ATOM	722	C5	CuN	X	1	25.660	28.658	51.750	0.00	0.00
ATOM	723	C5	CuN	X	1	25.660	28.658	67.660	0.00	0.00
ATOM	724	C4	CuN	X	1	3.360	8.038	36.450	0.00	0.00
ATOM	725	C4	CuN	X	1	3.360	8.038	52.370	0.00	0.00
ATOM	726	C4	CuN	X	1	3.360	8.038	68.280	0.00	0.00
ATOM	727	C4	CuN	X	1	3.360	18.928	36.450	0.00	0.00
ATOM	728	C4	CuN	X	1	3.360	18.928	52.370	0.00	0.00
ATOM	729	C4	CuN	X	1	3.360	18.928	68.280	0.00	0.00
ATOM	730	C4	CuN	X	1	3.360	29.828	36.450	0.00	0.00
ATOM	731	C4	CuN	X	1	3.360	29.828	52.370	0.00	0.00
ATOM	732	C4	CuN	X	1	3.360	29.828	68.280	0.00	0.00
ATOM	733	C4	CuN	X	1	14.250	8.038	36.450	0.00	0.00
ATOM	734	C4	CuN	X	1	14.250	8.038	52.370	0.00	0.00
ATOM	735	C4	CuN	X	1	14.250	8.038	68.280	0.00	0.00
ATOM	736	C4	CuN	X	1	14.250	18.928	36.450	0.00	0.00
ATOM	737	C4	CuN	X	1	14.250	18.928	52.370	0.00	0.00

ATOM 738 C4 CuN X 1 14.250 18.928 68.280 0.00 0.00

ATOM 739 C4 CuN X 1 14.250 29.828 36.450 0.00 0.00

ATOM 740 C4 CuN X 1 14.250 29.828 52.370 0.00 0.00

ATOM 741 C4 CuN X 1 14.250 29.828 68.280 0.00 0.00

ATOM 742 C4 CuN X 1 25.140 8.038 36.450 0.00 0.00

ATOM 743 C4 CuN X 1 25.140 8.038 52.370 0.00 0.00

ATOM 744 C4 CuN X 1 25.140 8.038 68.280 0.00 0.00

ATOM 745 C4 CuN X 1 25.140 18.928 36.450 0.00 0.00

ATOM 746 C4 CuN X 1 25.140 18.928 52.370 0.00 0.00

ATOM 747 C4 CuN X 1 25.140 18.928 68.280 0.00 0.00

ATOM 748 C4 CuN X 1 25.140 29.828 36.450 0.00 0.00

ATOM 749 C4 CuN X 1 25.140 29.828 52.370 0.00 0.00

ATOM 750 C4 CuN X 1 25.140 29.828 68.280 0.00 0.00

ATOM 751 C3 CuN X 1 3.310 8.078 37.850 0.00 0.00

ATOM 752 C3 CuN X 1 3.310 8.078 53.770 0.00 0.00

ATOM 753 C3 CuN X 1 3.310 8.078 69.680 0.00 0.00

ATOM 754 C3 CuN X 1 3.310 18.968 37.850 0.00 0.00

ATOM 755 C3 CuN X 1 3.310 18.968 53.770 0.00 0.00

ATOM 756 C3 CuN X 1 3.310 18.968 69.680 0.00 0.00

ATOM 757 C3 CuN X 1 3.310 29.858 37.850 0.00 0.00

ATOM 758 C3 CuN X 1 3.310 29.858 53.770 0.00 0.00

ATOM 759 C3 CuN X 1 3.310 29.858 69.680 0.00 0.00

ATOM 760 C3 CuN X 1 14.200 8.078 37.850 0.00 0.00

ATOM 761 C3 CuN X 1 14.200 8.078 53.770 0.00 0.00

ATOM 762 C3 CuN X 1 14.200 8.078 69.680 0.00 0.00

ATOM 763 C3 CuN X 1 14.200 18.968 37.850 0.00 0.00

ATOM 764 C3 CuN X 1 14.200 18.968 53.770 0.00 0.00

ATOM 765 C3 CuN X 1 14.200 18.968 69.680 0.00 0.00

ATOM 766 C3 CuN X 1 14.200 29.858 37.850 0.00 0.00

ATOM 767 C3 CuN X 1 14.200 29.858 53.770 0.00 0.00

ATOM	768	C3	CuN	X	1	14.200	29.858	69.680	0.00	0.00
ATOM	769	C3	CuN	X	1	25.090	8.078	37.850	0.00	0.00
ATOM	770	C3	CuN	X	1	25.090	8.078	53.770	0.00	0.00
ATOM	771	C3	CuN	X	1	25.090	8.078	69.680	0.00	0.00
ATOM	772	C3	CuN	X	1	25.090	18.968	37.850	0.00	0.00
ATOM	773	C3	CuN	X	1	25.090	18.968	53.770	0.00	0.00
ATOM	774	C3	CuN	X	1	25.090	18.968	69.680	0.00	0.00
ATOM	775	C3	CuN	X	1	25.090	29.858	37.850	0.00	0.00
ATOM	776	C3	CuN	X	1	25.090	29.858	53.770	0.00	0.00
ATOM	777	C3	CuN	X	1	25.090	29.858	69.680	0.00	0.00
ATOM	778	N1	CuN	X	1	3.720	7.038	38.630	0.00	0.00
ATOM	779	N1	CuN	X	1	3.720	7.038	54.540	0.00	0.00
ATOM	780	N1	CuN	X	1	3.720	7.038	70.450	0.00	0.00
ATOM	781	N1	CuN	X	1	3.720	17.928	38.630	0.00	0.00
ATOM	782	N1	CuN	X	1	3.720	17.928	54.540	0.00	0.00
ATOM	783	N1	CuN	X	1	3.720	17.928	70.450	0.00	0.00
ATOM	784	N1	CuN	X	1	3.720	28.818	38.630	0.00	0.00
ATOM	785	N1	CuN	X	1	3.720	28.818	54.540	0.00	0.00
ATOM	786	N1	CuN	X	1	3.720	28.818	70.450	0.00	0.00
ATOM	787	N1	CuN	X	1	14.610	7.038	38.630	0.00	0.00
ATOM	788	N1	CuN	X	1	14.610	7.038	54.540	0.00	0.00
ATOM	789	N1	CuN	X	1	14.610	7.038	70.450	0.00	0.00
ATOM	790	N1	CuN	X	1	14.610	17.928	38.630	0.00	0.00
ATOM	791	N1	CuN	X	1	14.610	17.928	54.540	0.00	0.00
ATOM	792	N1	CuN	X	1	14.610	17.928	70.450	0.00	0.00
ATOM	793	N1	CuN	X	1	14.610	28.818	38.630	0.00	0.00
ATOM	794	N1	CuN	X	1	14.610	28.818	54.540	0.00	0.00
ATOM	795	N1	CuN	X	1	14.610	28.818	70.450	0.00	0.00
ATOM	796	N1	CuN	X	1	25.510	7.038	38.630	0.00	0.00
ATOM	797	N1	CuN	X	1	25.510	7.038	54.540	0.00	0.00

ATOM	798	N1	CuN	X	1	25.510	7.038	70.450	0.00	0.00
ATOM	799	N1	CuN	X	1	25.510	17.928	38.630	0.00	0.00
ATOM	800	N1	CuN	X	1	25.510	17.928	54.540	0.00	0.00
ATOM	801	N1	CuN	X	1	25.510	17.928	70.450	0.00	0.00
ATOM	802	N1	CuN	X	1	25.510	28.818	38.630	0.00	0.00
ATOM	803	N1	CuN	X	1	25.510	28.818	54.540	0.00	0.00
ATOM	804	N1	CuN	X	1	25.510	28.818	70.450	0.00	0.00
ATOM	805	C3	CuN	X	1	4.210	5.928	38.040	0.00	0.00
ATOM	806	C3	CuN	X	1	4.210	5.928	53.950	0.00	0.00
ATOM	807	C3	CuN	X	1	4.210	5.928	69.860	0.00	0.00
ATOM	808	C3	CuN	X	1	4.210	16.818	38.040	0.00	0.00
ATOM	809	C3	CuN	X	1	4.210	16.818	53.950	0.00	0.00
ATOM	810	C3	CuN	X	1	4.210	16.818	69.860	0.00	0.00
ATOM	811	C3	CuN	X	1	4.210	27.708	38.040	0.00	0.00
ATOM	812	C3	CuN	X	1	4.210	27.708	53.950	0.00	0.00
ATOM	813	C3	CuN	X	1	4.210	27.708	69.860	0.00	0.00
ATOM	814	C3	CuN	X	1	15.100	5.928	38.040	0.00	0.00
ATOM	815	C3	CuN	X	1	15.100	5.928	53.950	0.00	0.00
ATOM	816	C3	CuN	X	1	15.100	5.928	69.860	0.00	0.00
ATOM	817	C3	CuN	X	1	15.100	16.818	38.040	0.00	0.00
ATOM	818	C3	CuN	X	1	15.100	16.818	53.950	0.00	0.00
ATOM	819	C3	CuN	X	1	15.100	16.818	69.860	0.00	0.00
ATOM	820	C3	CuN	X	1	15.100	27.708	38.040	0.00	0.00
ATOM	821	C3	CuN	X	1	15.100	27.708	53.950	0.00	0.00
ATOM	822	C3	CuN	X	1	15.100	27.708	69.860	0.00	0.00
ATOM	823	C3	CuN	X	1	26.000	5.928	38.040	0.00	0.00
ATOM	824	C3	CuN	X	1	26.000	5.928	53.950	0.00	0.00
ATOM	825	C3	CuN	X	1	26.000	5.928	69.860	0.00	0.00
ATOM	826	C3	CuN	X	1	26.000	16.818	38.040	0.00	0.00
ATOM	827	C3	CuN	X	1	26.000	16.818	53.950	0.00	0.00

ATOM	828	C3	CuN	X	1	26.000	16.818	69.860	0.00	0.00
ATOM	829	C3	CuN	X	1	26.000	27.708	38.040	0.00	0.00
ATOM	830	C3	CuN	X	1	26.000	27.708	53.950	0.00	0.00
ATOM	831	C3	CuN	X	1	26.000	27.708	69.860	0.00	0.00
ATOM	832	C4	CuN	X	1	4.310	5.798	36.640	0.00	0.00
ATOM	833	C4	CuN	X	1	4.310	5.798	52.550	0.00	0.00
ATOM	834	C4	CuN	X	1	4.310	5.798	68.470	0.00	0.00
ATOM	835	C4	CuN	X	1	4.310	16.688	36.640	0.00	0.00
ATOM	836	C4	CuN	X	1	4.310	16.688	52.550	0.00	0.00
ATOM	837	C4	CuN	X	1	4.310	16.688	68.470	0.00	0.00
ATOM	838	C4	CuN	X	1	4.310	27.578	36.640	0.00	0.00
ATOM	839	C4	CuN	X	1	4.310	27.578	52.550	0.00	0.00
ATOM	840	C4	CuN	X	1	4.310	27.578	68.470	0.00	0.00
ATOM	841	C4	CuN	X	1	15.200	5.798	36.640	0.00	0.00
ATOM	842	C4	CuN	X	1	15.200	5.798	52.550	0.00	0.00
ATOM	843	C4	CuN	X	1	15.200	5.798	68.470	0.00	0.00
ATOM	844	C4	CuN	X	1	15.200	16.688	36.640	0.00	0.00
ATOM	845	C4	CuN	X	1	15.200	16.688	52.550	0.00	0.00
ATOM	846	C4	CuN	X	1	15.200	16.688	68.470	0.00	0.00
ATOM	847	C4	CuN	X	1	15.200	27.578	36.640	0.00	0.00
ATOM	848	C4	CuN	X	1	15.200	27.578	52.550	0.00	0.00
ATOM	849	C4	CuN	X	1	15.200	27.578	68.470	0.00	0.00
ATOM	850	C4	CuN	X	1	26.090	5.798	36.640	0.00	0.00
ATOM	851	C4	CuN	X	1	26.090	5.798	52.550	0.00	0.00
ATOM	852	C4	CuN	X	1	26.090	5.798	68.470	0.00	0.00
ATOM	853	C4	CuN	X	1	26.090	16.688	36.640	0.00	0.00
ATOM	854	C4	CuN	X	1	26.090	16.688	52.550	0.00	0.00
ATOM	855	C4	CuN	X	1	26.090	16.688	68.470	0.00	0.00
ATOM	856	C4	CuN	X	1	26.090	27.578	36.640	0.00	0.00
ATOM	857	C4	CuN	X	1	26.090	27.578	52.550	0.00	0.00

ATOM 858 C4 CuN X 1 26.090 27.578 68.470 0.00 0.00
ATOM 859 H2 CuN X 1 4.110 5.288 29.740 0.00 0.00
ATOM 860 H2 CuN X 1 4.110 5.288 45.650 0.00 0.00
ATOM 861 H2 CuN X 1 4.110 5.288 61.570 0.00 0.00
ATOM 862 H2 CuN X 1 4.110 16.178 29.740 0.00 0.00
ATOM 863 H2 CuN X 1 4.110 16.178 45.650 0.00 0.00
ATOM 864 H2 CuN X 1 4.110 16.178 61.570 0.00 0.00
ATOM 865 H2 CuN X 1 4.110 27.068 29.740 0.00 0.00
ATOM 866 H2 CuN X 1 4.110 27.068 45.650 0.00 0.00
ATOM 867 H2 CuN X 1 4.110 27.068 61.570 0.00 0.00
ATOM 868 H2 CuN X 1 15.000 5.288 29.740 0.00 0.00
ATOM 869 H2 CuN X 1 15.000 5.288 45.650 0.00 0.00
ATOM 870 H2 CuN X 1 15.000 5.288 61.570 0.00 0.00
ATOM 871 H2 CuN X 1 15.000 16.178 29.740 0.00 0.00
ATOM 872 H2 CuN X 1 15.000 16.178 45.650 0.00 0.00
ATOM 873 H2 CuN X 1 15.000 16.178 61.570 0.00 0.00
ATOM 874 H2 CuN X 1 15.000 27.068 29.740 0.00 0.00
ATOM 875 H2 CuN X 1 15.000 27.068 45.650 0.00 0.00
ATOM 876 H2 CuN X 1 15.000 27.068 61.570 0.00 0.00
ATOM 877 H2 CuN X 1 25.890 5.288 29.740 0.00 0.00
ATOM 878 H2 CuN X 1 25.890 5.288 45.650 0.00 0.00
ATOM 879 H2 CuN X 1 25.890 5.288 61.570 0.00 0.00
ATOM 880 H2 CuN X 1 25.890 16.178 29.740 0.00 0.00
ATOM 881 H2 CuN X 1 25.890 16.178 45.650 0.00 0.00
ATOM 882 H2 CuN X 1 25.890 16.178 61.570 0.00 0.00
ATOM 883 H2 CuN X 1 25.890 27.068 29.740 0.00 0.00
ATOM 884 H2 CuN X 1 25.890 27.068 45.650 0.00 0.00
ATOM 885 H2 CuN X 1 25.890 27.068 61.570 0.00 0.00
ATOM 886 H3 CuN X 1 4.160 5.368 32.310 0.00 0.00
ATOM 887 H3 CuN X 1 4.160 5.368 48.230 0.00 0.00

ATOM	888	H3	CuN	X	1	4.160	5.368	64.140	0.00	0.00
ATOM	889	H3	CuN	X	1	4.160	16.258	32.310	0.00	0.00
ATOM	890	H3	CuN	X	1	4.160	16.258	48.230	0.00	0.00
ATOM	891	H3	CuN	X	1	4.160	16.258	64.140	0.00	0.00
ATOM	892	H3	CuN	X	1	4.160	27.148	32.310	0.00	0.00
ATOM	893	H3	CuN	X	1	4.160	27.148	48.230	0.00	0.00
ATOM	894	H3	CuN	X	1	4.160	27.148	64.140	0.00	0.00
ATOM	895	H3	CuN	X	1	15.050	5.368	32.310	0.00	0.00
ATOM	896	H3	CuN	X	1	15.050	5.368	48.230	0.00	0.00
ATOM	897	H3	CuN	X	1	15.050	5.368	64.140	0.00	0.00
ATOM	898	H3	CuN	X	1	15.050	16.258	32.310	0.00	0.00
ATOM	899	H3	CuN	X	1	15.050	16.258	48.230	0.00	0.00
ATOM	900	H3	CuN	X	1	15.050	16.258	64.140	0.00	0.00
ATOM	901	H3	CuN	X	1	15.050	27.148	32.310	0.00	0.00
ATOM	902	H3	CuN	X	1	15.050	27.148	48.230	0.00	0.00
ATOM	903	H3	CuN	X	1	15.050	27.148	64.140	0.00	0.00
ATOM	904	H3	CuN	X	1	25.940	5.368	32.310	0.00	0.00
ATOM	905	H3	CuN	X	1	25.940	5.368	48.230	0.00	0.00
ATOM	906	H3	CuN	X	1	25.940	5.368	64.140	0.00	0.00
ATOM	907	H3	CuN	X	1	25.940	16.258	32.310	0.00	0.00
ATOM	908	H3	CuN	X	1	25.940	16.258	48.230	0.00	0.00
ATOM	909	H3	CuN	X	1	25.940	16.258	64.140	0.00	0.00
ATOM	910	H3	CuN	X	1	25.940	27.148	32.310	0.00	0.00
ATOM	911	H3	CuN	X	1	25.940	27.148	48.230	0.00	0.00
ATOM	912	H3	CuN	X	1	25.940	27.148	64.140	0.00	0.00
ATOM	913	H3	CuN	X	1	3.270	9.618	32.030	0.00	0.00
ATOM	914	H3	CuN	X	1	3.270	9.618	47.940	0.00	0.00
ATOM	915	H3	CuN	X	1	3.270	9.618	63.850	0.00	0.00
ATOM	916	H3	CuN	X	1	3.270	20.508	32.030	0.00	0.00
ATOM	917	H3	CuN	X	1	3.270	20.508	47.940	0.00	0.00

ATOM	918	H3	CuN	X	1	3.270	20.508	63.850	0.00	0.00
ATOM	919	H3	CuN	X	1	14.160	9.618	32.030	0.00	0.00
ATOM	920	H3	CuN	X	1	14.160	9.618	47.940	0.00	0.00
ATOM	921	H3	CuN	X	1	14.160	9.618	63.850	0.00	0.00
ATOM	922	H3	CuN	X	1	14.160	20.508	32.030	0.00	0.00
ATOM	923	H3	CuN	X	1	14.160	20.508	47.940	0.00	0.00
ATOM	924	H3	CuN	X	1	14.160	20.508	63.850	0.00	0.00
ATOM	925	H3	CuN	X	1	25.050	9.618	32.030	0.00	0.00
ATOM	926	H3	CuN	X	1	25.050	9.618	47.940	0.00	0.00
ATOM	927	H3	CuN	X	1	25.050	9.618	63.850	0.00	0.00
ATOM	928	H3	CuN	X	1	25.050	20.508	32.030	0.00	0.00
ATOM	929	H3	CuN	X	1	25.050	20.508	47.940	0.00	0.00
ATOM	930	H3	CuN	X	1	25.050	20.508	63.850	0.00	0.00
ATOM	931	H2	CuN	X	1	3.270	9.348	29.460	0.00	0.00
ATOM	932	H2	CuN	X	1	3.270	9.348	45.380	0.00	0.00
ATOM	933	H2	CuN	X	1	3.270	9.348	61.290	0.00	0.00
ATOM	934	H2	CuN	X	1	3.270	20.238	29.460	0.00	0.00
ATOM	935	H2	CuN	X	1	3.270	20.238	45.380	0.00	0.00
ATOM	936	H2	CuN	X	1	3.270	20.238	61.290	0.00	0.00
ATOM	937	H2	CuN	X	1	3.270	31.128	29.460	0.00	0.00
ATOM	938	H2	CuN	X	1	3.270	31.128	45.380	0.00	0.00
ATOM	939	H2	CuN	X	1	3.270	31.128	61.290	0.00	0.00
ATOM	940	H2	CuN	X	1	14.160	9.348	29.460	0.00	0.00
ATOM	941	H2	CuN	X	1	14.160	9.348	45.380	0.00	0.00
ATOM	942	H2	CuN	X	1	14.160	9.348	61.290	0.00	0.00
ATOM	943	H2	CuN	X	1	14.160	20.238	29.460	0.00	0.00
ATOM	944	H2	CuN	X	1	14.160	20.238	45.380	0.00	0.00
ATOM	945	H2	CuN	X	1	14.160	20.238	61.290	0.00	0.00
ATOM	946	H2	CuN	X	1	14.160	31.128	29.460	0.00	0.00
ATOM	947	H2	CuN	X	1	14.160	31.128	45.380	0.00	0.00

ATOM	948	H2	CuN	X	1	14.160	31.128	61.290	0.00	0.00
ATOM	949	H2	CuN	X	1	25.050	9.348	29.460	0.00	0.00
ATOM	950	H2	CuN	X	1	25.050	9.348	45.380	0.00	0.00
ATOM	951	H2	CuN	X	1	25.050	9.348	61.290	0.00	0.00
ATOM	952	H2	CuN	X	1	25.050	20.238	29.460	0.00	0.00
ATOM	953	H2	CuN	X	1	25.050	20.238	45.380	0.00	0.00
ATOM	954	H2	CuN	X	1	25.050	20.238	61.290	0.00	0.00
ATOM	955	H2	CuN	X	1	25.050	31.128	29.460	0.00	0.00
ATOM	956	H2	CuN	X	1	25.050	31.128	45.380	0.00	0.00
ATOM	957	H2	CuN	X	1	25.050	31.128	61.290	0.00	0.00
ATOM	958	H3	CuN	X	1	3.010	8.878	35.840	0.00	0.00
ATOM	959	H3	CuN	X	1	3.010	8.878	51.750	0.00	0.00
ATOM	960	H3	CuN	X	1	3.010	8.878	67.670	0.00	0.00
ATOM	961	H3	CuN	X	1	3.010	19.778	35.840	0.00	0.00
ATOM	962	H3	CuN	X	1	3.010	19.778	51.750	0.00	0.00
ATOM	963	H3	CuN	X	1	3.010	19.778	67.670	0.00	0.00
ATOM	964	H3	CuN	X	1	3.010	30.668	35.840	0.00	0.00
ATOM	965	H3	CuN	X	1	3.010	30.668	51.750	0.00	0.00
ATOM	966	H3	CuN	X	1	3.010	30.668	67.670	0.00	0.00
ATOM	967	H3	CuN	X	1	13.900	8.878	35.840	0.00	0.00
ATOM	968	H3	CuN	X	1	13.900	8.878	51.750	0.00	0.00
ATOM	969	H3	CuN	X	1	13.900	8.878	67.670	0.00	0.00
ATOM	970	H3	CuN	X	1	13.900	19.778	35.840	0.00	0.00
ATOM	971	H3	CuN	X	1	13.900	19.778	51.750	0.00	0.00
ATOM	972	H3	CuN	X	1	13.900	19.778	67.670	0.00	0.00
ATOM	973	H3	CuN	X	1	13.900	30.668	35.840	0.00	0.00
ATOM	974	H3	CuN	X	1	13.900	30.668	51.750	0.00	0.00
ATOM	975	H3	CuN	X	1	13.900	30.668	67.670	0.00	0.00
ATOM	976	H3	CuN	X	1	24.790	8.878	35.840	0.00	0.00
ATOM	977	H3	CuN	X	1	24.790	8.878	51.750	0.00	0.00

```
ATOM    978  H3  CuN X   1      24.790   8.878  67.670  0.00  0.00
ATOM    979  H3  CuN X   1      24.790  19.778  35.840  0.00  0.00
ATOM    980  H3  CuN X   1      24.790  19.778  51.750  0.00  0.00
ATOM    981  H3  CuN X   1      24.790  19.778  67.670  0.00  0.00
ATOM    982  H3  CuN X   1      24.790  30.668  35.840  0.00  0.00
ATOM    983  H3  CuN X   1      24.790  30.668  51.750  0.00  0.00
ATOM    984  H3  CuN X   1      24.790  30.668  67.670  0.00  0.00
ATOM    985  H2  CuN X   1       2.930   8.948  38.410  0.00  0.00
ATOM    986  H2  CuN X   1       2.930   8.948  54.320  0.00  0.00
ATOM    987  H2  CuN X   1       2.930   8.948  70.240  0.00  0.00
ATOM    988  H2  CuN X   1       2.930  19.838  38.410  0.00  0.00
ATOM    989  H2  CuN X   1       2.930  19.838  54.320  0.00  0.00
ATOM    990  H2  CuN X   1       2.930  19.838  70.240  0.00  0.00
ATOM    991  H2  CuN X   1       2.930  30.728  38.410  0.00  0.00
ATOM    992  H2  CuN X   1       2.930  30.728  54.320  0.00  0.00
ATOM    993  H2  CuN X   1       2.930  30.728  70.240  0.00  0.00
ATOM    994  H2  CuN X   1      13.830   8.948  38.410  0.00  0.00
ATOM    995  H2  CuN X   1      13.830   8.948  54.320  0.00  0.00
ATOM    996  H2  CuN X   1      13.830   8.948  70.240  0.00  0.00
ATOM    997  H2  CuN X   1      13.830  19.838  38.410  0.00  0.00
ATOM    998  H2  CuN X   1      13.830  19.838  54.320  0.00  0.00
ATOM    999  H2  CuN X   1      13.830  19.838  70.240  0.00  0.00
ATOM   1000  H2  CuN X   1      13.830  30.728  38.410  0.00  0.00
ATOM   1001  H2  CuN X   1      13.830  30.728  54.320  0.00  0.00
ATOM   1002  H2  CuN X   1      13.830  30.728  70.240  0.00  0.00
ATOM   1003  H2  CuN X   1      24.720   8.948  38.410  0.00  0.00
ATOM   1004  H2  CuN X   1      24.720   8.948  54.320  0.00  0.00
ATOM   1005  H2  CuN X   1      24.720   8.948  70.240  0.00  0.00
ATOM   1006  H2  CuN X   1      24.720  19.838  38.410  0.00  0.00
ATOM   1007  H2  CuN X   1      24.720  19.838  54.320  0.00  0.00
```

ATOM 1008 H2 CuN X 1 24.720 19.838 70.240 0.00 0.00

ATOM 1009 H2 CuN X 1 24.720 30.728 38.410 0.00 0.00

ATOM 1010 H2 CuN X 1 24.720 30.728 54.320 0.00 0.00

ATOM 1011 H2 CuN X 1 24.720 30.728 70.240 0.00 0.00

ATOM 1012 H2 CuN X 1 4.510 5.118 38.730 0.00 0.00

ATOM 1013 H2 CuN X 1 4.510 5.118 54.640 0.00 0.00

ATOM 1014 H2 CuN X 1 4.510 5.118 70.560 0.00 0.00

ATOM 1015 H2 CuN X 1 4.510 16.018 38.730 0.00 0.00

ATOM 1016 H2 CuN X 1 4.510 16.018 54.640 0.00 0.00

ATOM 1017 H2 CuN X 1 4.510 16.018 70.560 0.00 0.00

ATOM 1018 H2 CuN X 1 4.510 26.908 38.730 0.00 0.00

ATOM 1019 H2 CuN X 1 4.510 26.908 54.640 0.00 0.00

ATOM 1020 H2 CuN X 1 4.510 26.908 70.560 0.00 0.00

ATOM 1021 H2 CuN X 1 15.410 5.118 38.730 0.00 0.00

ATOM 1022 H2 CuN X 1 15.410 5.118 54.640 0.00 0.00

ATOM 1023 H2 CuN X 1 15.410 5.118 70.560 0.00 0.00

ATOM 1024 H2 CuN X 1 15.410 16.018 38.730 0.00 0.00

ATOM 1025 H2 CuN X 1 15.410 16.018 54.640 0.00 0.00

ATOM 1026 H2 CuN X 1 15.410 16.018 70.560 0.00 0.00

ATOM 1027 H2 CuN X 1 15.410 26.908 38.730 0.00 0.00

ATOM 1028 H2 CuN X 1 15.410 26.908 54.640 0.00 0.00

ATOM 1029 H2 CuN X 1 15.410 26.908 70.560 0.00 0.00

ATOM 1030 H2 CuN X 1 26.300 5.118 38.730 0.00 0.00

ATOM 1031 H2 CuN X 1 26.300 5.118 54.640 0.00 0.00

ATOM 1032 H2 CuN X 1 26.300 5.118 70.560 0.00 0.00

ATOM 1033 H2 CuN X 1 26.300 16.018 38.730 0.00 0.00

ATOM 1034 H2 CuN X 1 26.300 16.018 54.640 0.00 0.00

ATOM 1035 H2 CuN X 1 26.300 16.018 70.560 0.00 0.00

ATOM 1036 H2 CuN X 1 26.300 26.908 38.730 0.00 0.00

ATOM 1037 H2 CuN X 1 26.300 26.908 54.640 0.00 0.00

ATOM 1038 H2 CuN X 1 26.300 26.908 70.560 0.00 0.00

ATOM 1039 H3 CuN X 1 4.700 4.878 36.170 0.00 0.00

ATOM 1040 H3 CuN X 1 4.700 4.878 52.080 0.00 0.00

ATOM 1041 H3 CuN X 1 4.700 4.878 68.000 0.00 0.00

ATOM 1042 H3 CuN X 1 4.700 15.768 36.170 0.00 0.00

ATOM 1043 H3 CuN X 1 4.700 15.768 52.080 0.00 0.00

ATOM 1044 H3 CuN X 1 4.700 15.768 68.000 0.00 0.00

ATOM 1045 H3 CuN X 1 4.700 26.668 36.170 0.00 0.00

ATOM 1046 H3 CuN X 1 4.700 26.668 52.080 0.00 0.00

ATOM 1047 H3 CuN X 1 4.700 26.668 68.000 0.00 0.00

ATOM 1048 H3 CuN X 1 15.590 4.878 36.170 0.00 0.00

ATOM 1049 H3 CuN X 1 15.590 4.878 52.080 0.00 0.00

ATOM 1050 H3 CuN X 1 15.590 4.878 68.000 0.00 0.00

ATOM 1051 H3 CuN X 1 15.590 15.768 36.170 0.00 0.00

ATOM 1052 H3 CuN X 1 15.590 15.768 52.080 0.00 0.00

ATOM 1053 H3 CuN X 1 15.590 15.768 68.000 0.00 0.00

ATOM 1054 H3 CuN X 1 15.590 26.668 36.170 0.00 0.00

ATOM 1055 H3 CuN X 1 15.590 26.668 52.080 0.00 0.00

ATOM 1056 H3 CuN X 1 15.590 26.668 68.000 0.00 0.00

ATOM 1057 H3 CuN X 1 26.490 4.878 36.170 0.00 0.00

ATOM 1058 H3 CuN X 1 26.490 4.878 52.080 0.00 0.00

ATOM 1059 H3 CuN X 1 26.490 4.878 68.000 0.00 0.00

ATOM 1060 H3 CuN X 1 26.490 15.768 36.170 0.00 0.00

ATOM 1061 H3 CuN X 1 26.490 15.768 52.080 0.00 0.00

ATOM 1062 H3 CuN X 1 26.490 15.768 68.000 0.00 0.00

ATOM 1063 H3 CuN X 1 26.490 26.668 36.170 0.00 0.00

ATOM 1064 H3 CuN X 1 26.490 26.668 52.080 0.00 0.00

ATOM 1065 H3 CuN X 1 26.490 26.668 68.000 0.00 0.00

ATOM 1066 H1 CuN X 1 7.870 7.228 28.290 0.00 0.00

ATOM 1067 H1 CuN X 1 7.870 7.228 44.200 0.00 0.00

ATOM	1068	H1	CuN	X	1	7.870	7.228	60.120	0.00	0.00
ATOM	1069	H1	CuN	X	1	7.870	7.228	76.030	0.00	0.00
ATOM	1070	H1	CuN	X	1	7.870	18.118	28.290	0.00	0.00
ATOM	1071	H1	CuN	X	1	7.870	18.118	44.200	0.00	0.00
ATOM	1072	H1	CuN	X	1	7.870	18.118	60.120	0.00	0.00
ATOM	1073	H1	CuN	X	1	7.870	18.118	76.030	0.00	0.00
ATOM	1074	H1	CuN	X	1	7.870	29.008	28.290	0.00	0.00
ATOM	1075	H1	CuN	X	1	7.870	29.008	44.200	0.00	0.00
ATOM	1076	H1	CuN	X	1	7.870	29.008	60.120	0.00	0.00
ATOM	1077	H1	CuN	X	1	7.870	29.008	76.030	0.00	0.00
ATOM	1078	H1	CuN	X	1	18.770	7.228	28.290	0.00	0.00
ATOM	1079	H1	CuN	X	1	18.770	7.228	44.200	0.00	0.00
ATOM	1080	H1	CuN	X	1	18.770	7.228	60.120	0.00	0.00
ATOM	1081	H1	CuN	X	1	18.770	7.228	76.030	0.00	0.00
ATOM	1082	H1	CuN	X	1	18.770	18.118	28.290	0.00	0.00
ATOM	1083	H1	CuN	X	1	18.770	18.118	44.200	0.00	0.00
ATOM	1084	H1	CuN	X	1	18.770	18.118	60.120	0.00	0.00
ATOM	1085	H1	CuN	X	1	18.770	18.118	76.030	0.00	0.00
ATOM	1086	H1	CuN	X	1	18.770	29.008	28.290	0.00	0.00
ATOM	1087	H1	CuN	X	1	18.770	29.008	44.200	0.00	0.00
ATOM	1088	H1	CuN	X	1	18.770	29.008	60.120	0.00	0.00
ATOM	1089	H1	CuN	X	1	18.770	29.008	76.030	0.00	0.00
ATOM	1090	H1	CuN	X	1	29.660	7.228	28.290	0.00	0.00
ATOM	1091	H1	CuN	X	1	29.660	7.228	44.200	0.00	0.00
ATOM	1092	H1	CuN	X	1	29.660	7.228	60.120	0.00	0.00
ATOM	1093	H1	CuN	X	1	29.660	7.228	76.030	0.00	0.00
ATOM	1094	H1	CuN	X	1	29.660	18.118	28.290	0.00	0.00
ATOM	1095	H1	CuN	X	1	29.660	18.118	44.200	0.00	0.00
ATOM	1096	H1	CuN	X	1	29.660	18.118	60.120	0.00	0.00
ATOM	1097	H1	CuN	X	1	29.660	18.118	76.030	0.00	0.00

ATOM 1098 H1 CuN X 1 29.660 29.008 28.290 0.00 0.00

ATOM 1099 H1 CuN X 1 29.660 29.008 44.200 0.00 0.00

ATOM 1100 H1 CuN X 1 29.660 29.008 60.120 0.00 0.00

ATOM 1101 H1 CuN X 1 29.660 29.008 76.030 0.00 0.00

ATOM 1102 H1 CuN X 1 7.880 7.098 23.960 0.00 0.00

ATOM 1103 H1 CuN X 1 7.880 7.098 39.870 0.00 0.00

ATOM 1104 H1 CuN X 1 7.880 7.098 55.790 0.00 0.00

ATOM 1105 H1 CuN X 1 7.880 7.098 71.700 0.00 0.00

ATOM 1106 H1 CuN X 1 7.880 17.998 23.960 0.00 0.00

ATOM 1107 H1 CuN X 1 7.880 17.998 39.870 0.00 0.00

ATOM 1108 H1 CuN X 1 7.880 17.998 55.790 0.00 0.00

ATOM 1109 H1 CuN X 1 7.880 17.998 71.700 0.00 0.00

ATOM 1110 H1 CuN X 1 7.880 28.888 23.960 0.00 0.00

ATOM 1111 H1 CuN X 1 7.880 28.888 39.870 0.00 0.00

ATOM 1112 H1 CuN X 1 7.880 28.888 55.790 0.00 0.00

ATOM 1113 H1 CuN X 1 7.880 28.888 71.700 0.00 0.00

ATOM 1114 H1 CuN X 1 18.770 7.098 23.960 0.00 0.00

ATOM 1115 H1 CuN X 1 18.770 7.098 39.870 0.00 0.00

ATOM 1116 H1 CuN X 1 18.770 7.098 55.790 0.00 0.00

ATOM 1117 H1 CuN X 1 18.770 7.098 71.700 0.00 0.00

ATOM 1118 H1 CuN X 1 18.770 17.998 23.960 0.00 0.00

ATOM 1119 H1 CuN X 1 18.770 17.998 39.870 0.00 0.00

ATOM 1120 H1 CuN X 1 18.770 17.998 55.790 0.00 0.00

ATOM 1121 H1 CuN X 1 18.770 17.998 71.700 0.00 0.00

ATOM 1122 H1 CuN X 1 18.770 28.888 23.960 0.00 0.00

ATOM 1123 H1 CuN X 1 18.770 28.888 39.870 0.00 0.00

ATOM 1124 H1 CuN X 1 18.770 28.888 55.790 0.00 0.00

ATOM 1125 H1 CuN X 1 18.770 28.888 71.700 0.00 0.00

ATOM 1126 H1 CuN X 1 29.660 7.098 23.960 0.00 0.00

ATOM 1127 H1 CuN X 1 29.660 7.098 39.870 0.00 0.00

ATOM	1128	H1	CuN	X	1	29.660	7.098	55.790	0.00	0.00
ATOM	1129	H1	CuN	X	1	29.660	7.098	71.700	0.00	0.00
ATOM	1130	H1	CuN	X	1	29.660	17.998	23.960	0.00	0.00
ATOM	1131	H1	CuN	X	1	29.660	17.998	39.870	0.00	0.00
ATOM	1132	H1	CuN	X	1	29.660	17.998	55.790	0.00	0.00
ATOM	1133	H1	CuN	X	1	29.660	17.998	71.700	0.00	0.00
ATOM	1134	H1	CuN	X	1	29.660	28.888	23.960	0.00	0.00
ATOM	1135	H1	CuN	X	1	29.660	28.888	39.870	0.00	0.00
ATOM	1136	H1	CuN	X	1	29.660	28.888	55.790	0.00	0.00
ATOM	1137	H1	CuN	X	1	29.660	28.888	71.700	0.00	0.00
ATOM	1138	H1	CuN	X	1	10.430	7.138	23.960	0.00	0.00
ATOM	1139	H1	CuN	X	1	10.430	7.138	39.870	0.00	0.00
ATOM	1140	H1	CuN	X	1	10.430	7.138	55.790	0.00	0.00
ATOM	1141	H1	CuN	X	1	10.430	7.138	71.700	0.00	0.00
ATOM	1142	H1	CuN	X	1	10.430	18.028	23.960	0.00	0.00
ATOM	1143	H1	CuN	X	1	10.430	18.028	39.870	0.00	0.00
ATOM	1144	H1	CuN	X	1	10.430	18.028	55.790	0.00	0.00
ATOM	1145	H1	CuN	X	1	10.430	18.028	71.700	0.00	0.00
ATOM	1146	H1	CuN	X	1	10.430	28.918	23.960	0.00	0.00
ATOM	1147	H1	CuN	X	1	10.430	28.918	39.870	0.00	0.00
ATOM	1148	H1	CuN	X	1	10.430	28.918	55.790	0.00	0.00
ATOM	1149	H1	CuN	X	1	10.430	28.918	71.700	0.00	0.00
ATOM	1150	H1	CuN	X	1	21.320	7.138	23.960	0.00	0.00
ATOM	1151	H1	CuN	X	1	21.320	7.138	39.870	0.00	0.00
ATOM	1152	H1	CuN	X	1	21.320	7.138	55.790	0.00	0.00
ATOM	1153	H1	CuN	X	1	21.320	7.138	71.700	0.00	0.00
ATOM	1154	H1	CuN	X	1	21.320	18.028	23.960	0.00	0.00
ATOM	1155	H1	CuN	X	1	21.320	18.028	39.870	0.00	0.00
ATOM	1156	H1	CuN	X	1	21.320	18.028	55.790	0.00	0.00
ATOM	1157	H1	CuN	X	1	21.320	18.028	71.700	0.00	0.00

ATOM	1158	H1	CuN	X	1	21.320	28.918	23.960	0.00	0.00
ATOM	1159	H1	CuN	X	1	21.320	28.918	39.870	0.00	0.00
ATOM	1160	H1	CuN	X	1	21.320	28.918	55.790	0.00	0.00
ATOM	1161	H1	CuN	X	1	21.320	28.918	71.700	0.00	0.00
ATOM	1162	H1	CuN	X	1	32.210	7.138	23.960	0.00	0.00
ATOM	1163	H1	CuN	X	1	32.210	7.138	39.870	0.00	0.00
ATOM	1164	H1	CuN	X	1	32.210	7.138	55.790	0.00	0.00
ATOM	1165	H1	CuN	X	1	32.210	7.138	71.700	0.00	0.00
ATOM	1166	H1	CuN	X	1	32.210	18.028	23.960	0.00	0.00
ATOM	1167	H1	CuN	X	1	32.210	18.028	39.870	0.00	0.00
ATOM	1168	H1	CuN	X	1	32.210	18.028	55.790	0.00	0.00
ATOM	1169	H1	CuN	X	1	32.210	18.028	71.700	0.00	0.00
ATOM	1170	H1	CuN	X	1	32.210	28.918	23.960	0.00	0.00
ATOM	1171	H1	CuN	X	1	32.210	28.918	39.870	0.00	0.00
ATOM	1172	H1	CuN	X	1	32.210	28.918	55.790	0.00	0.00
ATOM	1173	H1	CuN	X	1	32.210	28.918	71.700	0.00	0.00
ATOM	1174	H1	CuN	X	1	10.430	7.168	28.290	0.00	0.00
ATOM	1175	H1	CuN	X	1	10.430	7.168	44.210	0.00	0.00
ATOM	1176	H1	CuN	X	1	10.430	7.168	60.120	0.00	0.00
ATOM	1177	H1	CuN	X	1	10.430	7.168	76.040	0.00	0.00
ATOM	1178	H1	CuN	X	1	10.430	18.058	28.290	0.00	0.00
ATOM	1179	H1	CuN	X	1	10.430	18.058	44.210	0.00	0.00
ATOM	1180	H1	CuN	X	1	10.430	18.058	60.120	0.00	0.00
ATOM	1181	H1	CuN	X	1	10.430	18.058	76.040	0.00	0.00
ATOM	1182	H1	CuN	X	1	10.430	28.958	28.290	0.00	0.00
ATOM	1183	H1	CuN	X	1	10.430	28.958	44.210	0.00	0.00
ATOM	1184	H1	CuN	X	1	10.430	28.958	60.120	0.00	0.00
ATOM	1185	H1	CuN	X	1	10.430	28.958	76.040	0.00	0.00
ATOM	1186	H1	CuN	X	1	21.320	7.168	28.290	0.00	0.00
ATOM	1187	H1	CuN	X	1	21.320	7.168	44.210	0.00	0.00

ATOM	1188	H1	CuN	X	1	21.320	7.168	60.120	0.00	0.00
ATOM	1189	H1	CuN	X	1	21.320	7.168	76.040	0.00	0.00
ATOM	1190	H1	CuN	X	1	21.320	18.058	28.290	0.00	0.00
ATOM	1191	H1	CuN	X	1	21.320	18.058	44.210	0.00	0.00
ATOM	1192	H1	CuN	X	1	21.320	18.058	60.120	0.00	0.00
ATOM	1193	H1	CuN	X	1	21.320	18.058	76.040	0.00	0.00
ATOM	1194	H1	CuN	X	1	21.320	28.958	28.290	0.00	0.00
ATOM	1195	H1	CuN	X	1	21.320	28.958	44.210	0.00	0.00
ATOM	1196	H1	CuN	X	1	21.320	28.958	60.120	0.00	0.00
ATOM	1197	H1	CuN	X	1	21.320	28.958	76.040	0.00	0.00
ATOM	1198	H1	CuN	X	1	32.210	7.168	28.290	0.00	0.00
ATOM	1199	H1	CuN	X	1	32.210	7.168	44.210	0.00	0.00
ATOM	1200	H1	CuN	X	1	32.210	7.168	60.120	0.00	0.00
ATOM	1201	H1	CuN	X	1	32.210	7.168	76.040	0.00	0.00
ATOM	1202	H1	CuN	X	1	32.210	18.058	28.290	0.00	0.00
ATOM	1203	H1	CuN	X	1	32.210	18.058	44.210	0.00	0.00
ATOM	1204	H1	CuN	X	1	32.210	18.058	60.120	0.00	0.00
ATOM	1205	H1	CuN	X	1	32.210	18.058	76.040	0.00	0.00
ATOM	1206	H1	CuN	X	1	32.210	28.958	28.290	0.00	0.00
ATOM	1207	H1	CuN	X	1	32.210	28.958	44.210	0.00	0.00
ATOM	1208	H1	CuN	X	1	32.210	28.958	60.120	0.00	0.00
ATOM	1209	H1	CuN	X	1	32.210	28.958	76.040	0.00	0.00
ATOM	1210	O	CuN	X	1	3.400	5.198	25.080	0.00	0.00
ATOM	1211	O	CuN	X	1	3.400	5.198	41.000	0.00	0.00
ATOM	1212	O	CuN	X	1	3.400	5.198	56.910	0.00	0.00
ATOM	1213	O	CuN	X	1	3.400	5.198	72.830	0.00	0.00
ATOM	1214	O	CuN	X	1	3.400	16.088	25.080	0.00	0.00
ATOM	1215	O	CuN	X	1	3.400	16.088	41.000	0.00	0.00
ATOM	1216	O	CuN	X	1	3.400	16.088	56.910	0.00	0.00
ATOM	1217	O	CuN	X	1	3.400	16.088	72.830	0.00	0.00

ATOM	1218	O	CuN	X	1	3.400	26.978	25.080	0.00	0.00
ATOM	1219	O	CuN	X	1	3.400	26.978	41.000	0.00	0.00
ATOM	1220	O	CuN	X	1	3.400	26.978	56.910	0.00	0.00
ATOM	1221	O	CuN	X	1	3.400	26.978	72.830	0.00	0.00
ATOM	1222	O	CuN	X	1	14.300	5.198	25.080	0.00	0.00
ATOM	1223	O	CuN	X	1	14.300	5.198	41.000	0.00	0.00
ATOM	1224	O	CuN	X	1	14.300	5.198	56.910	0.00	0.00
ATOM	1225	O	CuN	X	1	14.300	5.198	72.830	0.00	0.00
ATOM	1226	O	CuN	X	1	14.300	16.088	25.080	0.00	0.00
ATOM	1227	O	CuN	X	1	14.300	16.088	41.000	0.00	0.00
ATOM	1228	O	CuN	X	1	14.300	16.088	56.910	0.00	0.00
ATOM	1229	O	CuN	X	1	14.300	16.088	72.830	0.00	0.00
ATOM	1230	O	CuN	X	1	14.300	26.978	25.080	0.00	0.00
ATOM	1231	O	CuN	X	1	14.300	26.978	41.000	0.00	0.00
ATOM	1232	O	CuN	X	1	14.300	26.978	56.910	0.00	0.00
ATOM	1233	O	CuN	X	1	14.300	26.978	72.830	0.00	0.00
ATOM	1234	O	CuN	X	1	25.190	5.198	25.080	0.00	0.00
ATOM	1235	O	CuN	X	1	25.190	5.198	41.000	0.00	0.00
ATOM	1236	O	CuN	X	1	25.190	5.198	56.910	0.00	0.00
ATOM	1237	O	CuN	X	1	25.190	5.198	72.830	0.00	0.00
ATOM	1238	O	CuN	X	1	25.190	16.088	25.080	0.00	0.00
ATOM	1239	O	CuN	X	1	25.190	16.088	41.000	0.00	0.00
ATOM	1240	O	CuN	X	1	25.190	16.088	56.910	0.00	0.00
ATOM	1241	O	CuN	X	1	25.190	16.088	72.830	0.00	0.00
ATOM	1242	O	CuN	X	1	25.190	26.978	25.080	0.00	0.00
ATOM	1243	O	CuN	X	1	25.190	26.978	41.000	0.00	0.00
ATOM	1244	O	CuN	X	1	25.190	26.978	56.910	0.00	0.00
ATOM	1245	O	CuN	X	1	25.190	26.978	72.830	0.00	0.00
ATOM	1246	O	CuN	X	1	4.060	5.198	27.280	0.00	0.00
ATOM	1247	O	CuN	X	1	4.060	5.198	43.200	0.00	0.00

ATOM 1248 O CuN X 1 4.060 5.198 59.110 0.00 0.00

ATOM 1249 O CuN X 1 4.060 5.198 75.020 0.00 0.00

ATOM 1250 O CuN X 1 4.060 16.098 27.280 0.00 0.00

ATOM 1251 O CuN X 1 4.060 16.098 43.200 0.00 0.00

ATOM 1252 O CuN X 1 4.060 16.098 59.110 0.00 0.00

ATOM 1253 O CuN X 1 4.060 16.098 75.020 0.00 0.00

ATOM 1254 O CuN X 1 4.060 26.988 27.280 0.00 0.00

ATOM 1255 O CuN X 1 4.060 26.988 43.200 0.00 0.00

ATOM 1256 O CuN X 1 4.060 26.988 59.110 0.00 0.00

ATOM 1257 O CuN X 1 4.060 26.988 75.020 0.00 0.00

ATOM 1258 O CuN X 1 14.960 5.198 27.280 0.00 0.00

ATOM 1259 O CuN X 1 14.960 5.198 43.200 0.00 0.00

ATOM 1260 O CuN X 1 14.960 5.198 59.110 0.00 0.00

ATOM 1261 O CuN X 1 14.960 5.198 75.020 0.00 0.00

ATOM 1262 O CuN X 1 14.960 16.098 27.280 0.00 0.00

ATOM 1263 O CuN X 1 14.960 16.098 43.200 0.00 0.00

ATOM 1264 O CuN X 1 14.960 16.098 59.110 0.00 0.00

ATOM 1265 O CuN X 1 14.960 16.098 75.020 0.00 0.00

ATOM 1266 O CuN X 1 14.960 26.988 27.280 0.00 0.00

ATOM 1267 O CuN X 1 14.960 26.988 43.200 0.00 0.00

ATOM 1268 O CuN X 1 14.960 26.988 59.110 0.00 0.00

ATOM 1269 O CuN X 1 14.960 26.988 75.020 0.00 0.00

ATOM 1270 O CuN X 1 25.850 5.198 27.280 0.00 0.00

ATOM 1271 O CuN X 1 25.850 5.198 43.200 0.00 0.00

ATOM 1272 O CuN X 1 25.850 5.198 59.110 0.00 0.00

ATOM 1273 O CuN X 1 25.850 5.198 75.020 0.00 0.00

ATOM 1274 O CuN X 1 25.850 16.098 27.280 0.00 0.00

ATOM 1275 O CuN X 1 25.850 16.098 43.200 0.00 0.00

ATOM 1276 O CuN X 1 25.850 16.098 59.110 0.00 0.00

ATOM 1277 O CuN X 1 25.850 16.098 75.020 0.00 0.00

ATOM	1278	O	CuN	X	1	25.850	26.988	27.280	0.00	0.00
ATOM	1279	O	CuN	X	1	25.850	26.988	43.200	0.00	0.00
ATOM	1280	O	CuN	X	1	25.850	26.988	59.110	0.00	0.00
ATOM	1281	O	CuN	X	1	25.850	26.988	75.020	0.00	0.00
ATOM	1282	O	CuN	X	1	3.370	9.158	27.160	0.00	0.00
ATOM	1283	O	CuN	X	1	3.370	9.158	43.080	0.00	0.00
ATOM	1284	O	CuN	X	1	3.370	9.158	58.990	0.00	0.00
ATOM	1285	O	CuN	X	1	3.370	9.158	74.910	0.00	0.00
ATOM	1286	O	CuN	X	1	3.370	20.048	27.160	0.00	0.00
ATOM	1287	O	CuN	X	1	3.370	20.048	43.080	0.00	0.00
ATOM	1288	O	CuN	X	1	3.370	20.048	58.990	0.00	0.00
ATOM	1289	O	CuN	X	1	3.370	20.048	74.910	0.00	0.00
ATOM	1290	O	CuN	X	1	14.260	9.158	27.160	0.00	0.00
ATOM	1291	O	CuN	X	1	14.260	9.158	43.080	0.00	0.00
ATOM	1292	O	CuN	X	1	14.260	9.158	58.990	0.00	0.00
ATOM	1293	O	CuN	X	1	14.260	9.158	74.910	0.00	0.00
ATOM	1294	O	CuN	X	1	14.260	20.048	27.160	0.00	0.00
ATOM	1295	O	CuN	X	1	14.260	20.048	43.080	0.00	0.00
ATOM	1296	O	CuN	X	1	14.260	20.048	58.990	0.00	0.00
ATOM	1297	O	CuN	X	1	14.260	20.048	74.910	0.00	0.00
ATOM	1298	O	CuN	X	1	25.150	9.158	27.160	0.00	0.00
ATOM	1299	O	CuN	X	1	25.150	9.158	43.080	0.00	0.00
ATOM	1300	O	CuN	X	1	25.150	9.158	58.990	0.00	0.00
ATOM	1301	O	CuN	X	1	25.150	9.158	74.910	0.00	0.00
ATOM	1302	O	CuN	X	1	25.150	20.048	27.160	0.00	0.00
ATOM	1303	O	CuN	X	1	25.150	20.048	43.080	0.00	0.00
ATOM	1304	O	CuN	X	1	25.150	20.048	58.990	0.00	0.00
ATOM	1305	O	CuN	X	1	25.150	20.048	74.910	0.00	0.00
ATOM	1306	O	CuN	X	1	4.000	9.158	24.960	0.00	0.00
ATOM	1307	O	CuN	X	1	4.000	9.158	40.880	0.00	0.00

ATOM 1308 O CuN X 1 4.000 9.158 56.790 0.00 0.00
ATOM 1309 O CuN X 1 4.000 9.158 72.700 0.00 0.00
ATOM 1310 O CuN X 1 4.000 20.048 24.960 0.00 0.00
ATOM 1311 O CuN X 1 4.000 20.048 40.880 0.00 0.00
ATOM 1312 O CuN X 1 4.000 20.048 56.790 0.00 0.00
ATOM 1313 O CuN X 1 4.000 20.048 72.700 0.00 0.00
ATOM 1314 O CuN X 1 14.900 9.158 24.960 0.00 0.00
ATOM 1315 O CuN X 1 14.900 9.158 40.880 0.00 0.00
ATOM 1316 O CuN X 1 14.900 9.158 56.790 0.00 0.00
ATOM 1317 O CuN X 1 14.900 9.158 72.700 0.00 0.00
ATOM 1318 O CuN X 1 14.900 20.048 24.960 0.00 0.00
ATOM 1319 O CuN X 1 14.900 20.048 40.880 0.00 0.00
ATOM 1320 O CuN X 1 14.900 20.048 56.790 0.00 0.00
ATOM 1321 O CuN X 1 14.900 20.048 72.700 0.00 0.00
ATOM 1322 O CuN X 1 25.790 9.158 24.960 0.00 0.00
ATOM 1323 O CuN X 1 25.790 9.158 40.880 0.00 0.00
ATOM 1324 O CuN X 1 25.790 9.158 56.790 0.00 0.00
ATOM 1325 O CuN X 1 25.790 9.158 72.700 0.00 0.00
ATOM 1326 O CuN X 1 25.790 20.048 24.960 0.00 0.00
ATOM 1327 O CuN X 1 25.790 20.048 40.880 0.00 0.00
ATOM 1328 O CuN X 1 25.790 20.048 56.790 0.00 0.00
ATOM 1329 O CuN X 1 25.790 20.048 72.700 0.00 0.00
ATOM 1330 C1 CuN X 1 3.690 31.508 26.060 0.00 0.00
ATOM 1331 C1 CuN X 1 3.690 31.508 41.980 0.00 0.00
ATOM 1332 C1 CuN X 1 3.690 31.508 57.890 0.00 0.00
ATOM 1333 C1 CuN X 1 3.690 31.508 73.810 0.00 0.00
ATOM 1334 C1 CuN X 1 3.690 9.718 26.060 0.00 0.00
ATOM 1335 C1 CuN X 1 3.690 9.718 41.980 0.00 0.00
ATOM 1336 C1 CuN X 1 3.690 9.718 57.890 0.00 0.00
ATOM 1337 C1 CuN X 1 3.690 9.718 73.810 0.00 0.00

ATOM	1338	C1	CuN	X	1	3.690	20.608	26.060	0.00	0.00
ATOM	1339	C1	CuN	X	1	3.690	20.608	41.980	0.00	0.00
ATOM	1340	C1	CuN	X	1	3.690	20.608	57.890	0.00	0.00
ATOM	1341	C1	CuN	X	1	3.690	20.608	73.810	0.00	0.00
ATOM	1342	C1	CuN	X	1	14.580	31.508	26.060	0.00	0.00
ATOM	1343	C1	CuN	X	1	14.580	31.508	41.980	0.00	0.00
ATOM	1344	C1	CuN	X	1	14.580	31.508	57.890	0.00	0.00
ATOM	1345	C1	CuN	X	1	14.580	31.508	73.810	0.00	0.00
ATOM	1346	C1	CuN	X	1	14.580	9.718	26.060	0.00	0.00
ATOM	1347	C1	CuN	X	1	14.580	9.718	41.980	0.00	0.00
ATOM	1348	C1	CuN	X	1	14.580	9.718	57.890	0.00	0.00
ATOM	1349	C1	CuN	X	1	14.580	9.718	73.810	0.00	0.00
ATOM	1350	C1	CuN	X	1	14.580	20.608	26.060	0.00	0.00
ATOM	1351	C1	CuN	X	1	14.580	20.608	41.980	0.00	0.00
ATOM	1352	C1	CuN	X	1	14.580	20.608	57.890	0.00	0.00
ATOM	1353	C1	CuN	X	1	14.580	20.608	73.810	0.00	0.00
ATOM	1354	C1	CuN	X	1	25.470	31.508	26.060	0.00	0.00
ATOM	1355	C1	CuN	X	1	25.470	31.508	41.980	0.00	0.00
ATOM	1356	C1	CuN	X	1	25.470	31.508	57.890	0.00	0.00
ATOM	1357	C1	CuN	X	1	25.470	31.508	73.810	0.00	0.00
ATOM	1358	C1	CuN	X	1	25.470	9.718	26.060	0.00	0.00
ATOM	1359	C1	CuN	X	1	25.470	9.718	41.980	0.00	0.00
ATOM	1360	C1	CuN	X	1	25.470	9.718	57.890	0.00	0.00
ATOM	1361	C1	CuN	X	1	25.470	9.718	73.810	0.00	0.00
ATOM	1362	C1	CuN	X	1	25.470	20.608	26.060	0.00	0.00
ATOM	1363	C1	CuN	X	1	25.470	20.608	41.980	0.00	0.00
ATOM	1364	C1	CuN	X	1	25.470	20.608	57.890	0.00	0.00
ATOM	1365	C1	CuN	X	1	25.470	20.608	73.810	0.00	0.00
ATOM	1366	C2	CuN	X	1	3.730	3.138	26.170	0.00	0.00
ATOM	1367	C2	CuN	X	1	3.730	3.138	42.080	0.00	0.00

ATOM	1368	C2	CuN	X	1	3.730	3.138	58.000	0.00	0.00
ATOM	1369	C2	CuN	X	1	3.730	3.138	73.910	0.00	0.00
ATOM	1370	C2	CuN	X	1	3.730	14.028	26.170	0.00	0.00
ATOM	1371	C2	CuN	X	1	3.730	14.028	42.080	0.00	0.00
ATOM	1372	C2	CuN	X	1	3.730	14.028	58.000	0.00	0.00
ATOM	1373	C2	CuN	X	1	3.730	14.028	73.910	0.00	0.00
ATOM	1374	C2	CuN	X	1	3.730	24.918	26.170	0.00	0.00
ATOM	1375	C2	CuN	X	1	3.730	24.918	42.080	0.00	0.00
ATOM	1376	C2	CuN	X	1	3.730	24.918	58.000	0.00	0.00
ATOM	1377	C2	CuN	X	1	3.730	24.918	73.910	0.00	0.00
ATOM	1378	C2	CuN	X	1	14.620	3.138	26.170	0.00	0.00
ATOM	1379	C2	CuN	X	1	14.620	3.138	42.080	0.00	0.00
ATOM	1380	C2	CuN	X	1	14.620	3.138	58.000	0.00	0.00
ATOM	1381	C2	CuN	X	1	14.620	3.138	73.910	0.00	0.00
ATOM	1382	C2	CuN	X	1	14.620	14.028	26.170	0.00	0.00
ATOM	1383	C2	CuN	X	1	14.620	14.028	42.080	0.00	0.00
ATOM	1384	C2	CuN	X	1	14.620	14.028	58.000	0.00	0.00
ATOM	1385	C2	CuN	X	1	14.620	14.028	73.910	0.00	0.00
ATOM	1386	C2	CuN	X	1	14.620	24.918	26.170	0.00	0.00
ATOM	1387	C2	CuN	X	1	14.620	24.918	42.080	0.00	0.00
ATOM	1388	C2	CuN	X	1	14.620	24.918	58.000	0.00	0.00
ATOM	1389	C2	CuN	X	1	14.620	24.918	73.910	0.00	0.00
ATOM	1390	C2	CuN	X	1	25.520	3.138	26.170	0.00	0.00
ATOM	1391	C2	CuN	X	1	25.520	3.138	42.080	0.00	0.00
ATOM	1392	C2	CuN	X	1	25.520	3.138	58.000	0.00	0.00
ATOM	1393	C2	CuN	X	1	25.520	3.138	73.910	0.00	0.00
ATOM	1394	C2	CuN	X	1	25.520	14.028	26.170	0.00	0.00
ATOM	1395	C2	CuN	X	1	25.520	14.028	42.080	0.00	0.00
ATOM	1396	C2	CuN	X	1	25.520	14.028	58.000	0.00	0.00
ATOM	1397	C2	CuN	X	1	25.520	14.028	73.910	0.00	0.00

```
ATOM   1398  C2  CuN X   1      25.520  24.918  26.170  0.00  0.00
ATOM   1399  C2  CuN X   1      25.520  24.918  42.080  0.00  0.00
ATOM   1400  C2  CuN X   1      25.520  24.918  58.000  0.00  0.00
ATOM   1401  C2  CuN X   1      25.520  24.918  73.910  0.00  0.00
ATOM   1402  C2  CuN X   1       2.500   2.448  26.100  0.00  0.00
ATOM   1403  C2  CuN X   1       2.500   2.448  42.020  0.00  0.00
ATOM   1404  C2  CuN X   1       2.500   2.448  57.930  0.00  0.00
ATOM   1405  C2  CuN X   1       2.500   2.448  73.850  0.00  0.00
ATOM   1406  C2  CuN X   1       2.500  13.338  26.100  0.00  0.00
ATOM   1407  C2  CuN X   1       2.500  13.338  42.020  0.00  0.00
ATOM   1408  C2  CuN X   1       2.500  13.338  57.930  0.00  0.00
ATOM   1409  C2  CuN X   1       2.500  13.338  73.850  0.00  0.00
ATOM   1410  C2  CuN X   1       2.500  24.228  26.100  0.00  0.00
ATOM   1411  C2  CuN X   1       2.500  24.228  42.020  0.00  0.00
ATOM   1412  C2  CuN X   1       2.500  24.228  57.930  0.00  0.00
ATOM   1413  C2  CuN X   1       2.500  24.228  73.850  0.00  0.00
ATOM   1414  C2  CuN X   1      13.390   2.448  26.100  0.00  0.00
ATOM   1415  C2  CuN X   1      13.390   2.448  42.020  0.00  0.00
ATOM   1416  C2  CuN X   1      13.390   2.448  57.930  0.00  0.00
ATOM   1417  C2  CuN X   1      13.390   2.448  73.850  0.00  0.00
ATOM   1418  C2  CuN X   1      13.390  13.338  26.100  0.00  0.00
ATOM   1419  C2  CuN X   1      13.390  13.338  42.020  0.00  0.00
ATOM   1420  C2  CuN X   1      13.390  13.338  57.930  0.00  0.00
ATOM   1421  C2  CuN X   1      13.390  13.338  73.850  0.00  0.00
ATOM   1422  C2  CuN X   1      13.390  24.228  26.100  0.00  0.00
ATOM   1423  C2  CuN X   1      13.390  24.228  42.020  0.00  0.00
ATOM   1424  C2  CuN X   1      13.390  24.228  57.930  0.00  0.00
ATOM   1425  C2  CuN X   1      13.390  24.228  73.850  0.00  0.00
ATOM   1426  C2  CuN X   1      24.280   2.448  26.100  0.00  0.00
ATOM   1427  C2  CuN X   1      24.280   2.448  42.020  0.00  0.00
```

ATOM	1428	C2	CuN	X	1	24.280	2.448	57.930	0.00	0.00
ATOM	1429	C2	CuN	X	1	24.280	2.448	73.850	0.00	0.00
ATOM	1430	C2	CuN	X	1	24.280	13.338	26.100	0.00	0.00
ATOM	1431	C2	CuN	X	1	24.280	13.338	42.020	0.00	0.00
ATOM	1432	C2	CuN	X	1	24.280	13.338	57.930	0.00	0.00
ATOM	1433	C2	CuN	X	1	24.280	13.338	73.850	0.00	0.00
ATOM	1434	C2	CuN	X	1	24.280	24.228	26.100	0.00	0.00
ATOM	1435	C2	CuN	X	1	24.280	24.228	42.020	0.00	0.00
ATOM	1436	C2	CuN	X	1	24.280	24.228	57.930	0.00	0.00
ATOM	1437	C2	CuN	X	1	24.280	24.228	73.850	0.00	0.00
ATOM	1438	C2	CuN	X	1	2.480	1.038	26.090	0.00	0.00
ATOM	1439	C2	CuN	X	1	2.480	1.038	42.000	0.00	0.00
ATOM	1440	C2	CuN	X	1	2.480	1.038	57.910	0.00	0.00
ATOM	1441	C2	CuN	X	1	2.480	1.038	73.830	0.00	0.00
ATOM	1442	C2	CuN	X	1	2.480	11.928	26.090	0.00	0.00
ATOM	1443	C2	CuN	X	1	2.480	11.928	42.000	0.00	0.00
ATOM	1444	C2	CuN	X	1	2.480	11.928	57.910	0.00	0.00
ATOM	1445	C2	CuN	X	1	2.480	11.928	73.830	0.00	0.00
ATOM	1446	C2	CuN	X	1	2.480	22.828	26.090	0.00	0.00
ATOM	1447	C2	CuN	X	1	2.480	22.828	42.000	0.00	0.00
ATOM	1448	C2	CuN	X	1	2.480	22.828	57.910	0.00	0.00
ATOM	1449	C2	CuN	X	1	2.480	22.828	73.830	0.00	0.00
ATOM	1450	C2	CuN	X	1	13.380	1.038	26.090	0.00	0.00
ATOM	1451	C2	CuN	X	1	13.380	1.038	42.000	0.00	0.00
ATOM	1452	C2	CuN	X	1	13.380	1.038	57.910	0.00	0.00
ATOM	1453	C2	CuN	X	1	13.380	1.038	73.830	0.00	0.00
ATOM	1454	C2	CuN	X	1	13.380	11.928	26.090	0.00	0.00
ATOM	1455	C2	CuN	X	1	13.380	11.928	42.000	0.00	0.00
ATOM	1456	C2	CuN	X	1	13.380	11.928	57.910	0.00	0.00
ATOM	1457	C2	CuN	X	1	13.380	11.928	73.830	0.00	0.00

ATOM 1458 C2 CuN X 1 13.380 22.828 26.090 0.00 0.00

ATOM 1459 C2 CuN X 1 13.380 22.828 42.000 0.00 0.00

ATOM 1460 C2 CuN X 1 13.380 22.828 57.910 0.00 0.00

ATOM 1461 C2 CuN X 1 13.380 22.828 73.830 0.00 0.00

ATOM 1462 C2 CuN X 1 24.270 1.038 26.090 0.00 0.00

ATOM 1463 C2 CuN X 1 24.270 1.038 42.000 0.00 0.00

ATOM 1464 C2 CuN X 1 24.270 1.038 57.910 0.00 0.00

ATOM 1465 C2 CuN X 1 24.270 1.038 73.830 0.00 0.00

ATOM 1466 C2 CuN X 1 24.270 11.928 26.090 0.00 0.00

ATOM 1467 C2 CuN X 1 24.270 11.928 42.000 0.00 0.00

ATOM 1468 C2 CuN X 1 24.270 11.928 57.910 0.00 0.00

ATOM 1469 C2 CuN X 1 24.270 11.928 73.830 0.00 0.00

ATOM 1470 C2 CuN X 1 24.270 22.828 26.090 0.00 0.00

ATOM 1471 C2 CuN X 1 24.270 22.828 42.000 0.00 0.00

ATOM 1472 C2 CuN X 1 24.270 22.828 57.910 0.00 0.00

ATOM 1473 C2 CuN X 1 24.270 22.828 73.830 0.00 0.00

ATOM 1474 C2 CuN X 1 3.700 0.328 26.090 0.00 0.00

ATOM 1475 C2 CuN X 1 3.700 0.328 42.000 0.00 0.00

ATOM 1476 C2 CuN X 1 3.700 0.328 57.910 0.00 0.00

ATOM 1477 C2 CuN X 1 3.700 0.328 73.830 0.00 0.00

ATOM 1478 C2 CuN X 1 3.700 11.218 26.090 0.00 0.00

ATOM 1479 C2 CuN X 1 3.700 11.218 42.000 0.00 0.00

ATOM 1480 C2 CuN X 1 3.700 11.218 57.910 0.00 0.00

ATOM 1481 C2 CuN X 1 3.700 11.218 73.830 0.00 0.00

ATOM 1482 C2 CuN X 1 3.700 22.118 26.090 0.00 0.00

ATOM 1483 C2 CuN X 1 3.700 22.118 42.000 0.00 0.00

ATOM 1484 C2 CuN X 1 3.700 22.118 57.910 0.00 0.00

ATOM 1485 C2 CuN X 1 3.700 22.118 73.830 0.00 0.00

ATOM 1486 C2 CuN X 1 14.600 0.328 26.090 0.00 0.00

ATOM 1487 C2 CuN X 1 14.600 0.328 42.000 0.00 0.00

ATOM	1488	C2	CuN	X	1	14.600	0.328	57.910	0.00	0.00
ATOM	1489	C2	CuN	X	1	14.600	0.328	73.830	0.00	0.00
ATOM	1490	C2	CuN	X	1	14.600	11.218	26.090	0.00	0.00
ATOM	1491	C2	CuN	X	1	14.600	11.218	42.000	0.00	0.00
ATOM	1492	C2	CuN	X	1	14.600	11.218	57.910	0.00	0.00
ATOM	1493	C2	CuN	X	1	14.600	11.218	73.830	0.00	0.00
ATOM	1494	C2	CuN	X	1	14.600	22.118	26.090	0.00	0.00
ATOM	1495	C2	CuN	X	1	14.600	22.118	42.000	0.00	0.00
ATOM	1496	C2	CuN	X	1	14.600	22.118	57.910	0.00	0.00
ATOM	1497	C2	CuN	X	1	14.600	22.118	73.830	0.00	0.00
ATOM	1498	C2	CuN	X	1	25.490	0.328	26.090	0.00	0.00
ATOM	1499	C2	CuN	X	1	25.490	0.328	42.000	0.00	0.00
ATOM	1500	C2	CuN	X	1	25.490	0.328	57.910	0.00	0.00
ATOM	1501	C2	CuN	X	1	25.490	0.328	73.830	0.00	0.00
ATOM	1502	C2	CuN	X	1	25.490	11.218	26.090	0.00	0.00
ATOM	1503	C2	CuN	X	1	25.490	11.218	42.000	0.00	0.00
ATOM	1504	C2	CuN	X	1	25.490	11.218	57.910	0.00	0.00
ATOM	1505	C2	CuN	X	1	25.490	11.218	73.830	0.00	0.00
ATOM	1506	C2	CuN	X	1	25.490	22.118	26.090	0.00	0.00
ATOM	1507	C2	CuN	X	1	25.490	22.118	42.000	0.00	0.00
ATOM	1508	C2	CuN	X	1	25.490	22.118	57.910	0.00	0.00
ATOM	1509	C2	CuN	X	1	25.490	22.118	73.830	0.00	0.00
ATOM	1510	C2	CuN	X	1	4.940	1.018	26.130	0.00	0.00
ATOM	1511	C2	CuN	X	1	4.940	1.018	42.040	0.00	0.00
ATOM	1512	C2	CuN	X	1	4.940	1.018	57.960	0.00	0.00
ATOM	1513	C2	CuN	X	1	4.940	1.018	73.870	0.00	0.00
ATOM	1514	C2	CuN	X	1	4.940	11.908	26.130	0.00	0.00
ATOM	1515	C2	CuN	X	1	4.940	11.908	42.040	0.00	0.00
ATOM	1516	C2	CuN	X	1	4.940	11.908	57.960	0.00	0.00
ATOM	1517	C2	CuN	X	1	4.940	11.908	73.870	0.00	0.00

ATOM 1518 C2 CuN X 1 4.940 22.798 26.130 0.00 0.00

ATOM 1519 C2 CuN X 1 4.940 22.798 42.040 0.00 0.00

ATOM 1520 C2 CuN X 1 4.940 22.798 57.960 0.00 0.00

ATOM 1521 C2 CuN X 1 4.940 22.798 73.870 0.00 0.00

ATOM 1522 C2 CuN X 1 15.830 1.018 26.130 0.00 0.00

ATOM 1523 C2 CuN X 1 15.830 1.018 42.040 0.00 0.00

ATOM 1524 C2 CuN X 1 15.830 1.018 57.960 0.00 0.00

ATOM 1525 C2 CuN X 1 15.830 1.018 73.870 0.00 0.00

ATOM 1526 C2 CuN X 1 15.830 11.908 26.130 0.00 0.00

ATOM 1527 C2 CuN X 1 15.830 11.908 42.040 0.00 0.00

ATOM 1528 C2 CuN X 1 15.830 11.908 57.960 0.00 0.00

ATOM 1529 C2 CuN X 1 15.830 11.908 73.870 0.00 0.00

ATOM 1530 C2 CuN X 1 15.830 22.798 26.130 0.00 0.00

ATOM 1531 C2 CuN X 1 15.830 22.798 42.040 0.00 0.00

ATOM 1532 C2 CuN X 1 15.830 22.798 57.960 0.00 0.00

ATOM 1533 C2 CuN X 1 15.830 22.798 73.870 0.00 0.00

ATOM 1534 C2 CuN X 1 26.720 1.018 26.130 0.00 0.00

ATOM 1535 C2 CuN X 1 26.720 1.018 42.040 0.00 0.00

ATOM 1536 C2 CuN X 1 26.720 1.018 57.960 0.00 0.00

ATOM 1537 C2 CuN X 1 26.720 1.018 73.870 0.00 0.00

ATOM 1538 C2 CuN X 1 26.720 11.908 26.130 0.00 0.00

ATOM 1539 C2 CuN X 1 26.720 11.908 42.040 0.00 0.00

ATOM 1540 C2 CuN X 1 26.720 11.908 57.960 0.00 0.00

ATOM 1541 C2 CuN X 1 26.720 11.908 73.870 0.00 0.00

ATOM 1542 C2 CuN X 1 26.720 22.798 26.130 0.00 0.00

ATOM 1543 C2 CuN X 1 26.720 22.798 42.040 0.00 0.00

ATOM 1544 C2 CuN X 1 26.720 22.798 57.960 0.00 0.00

ATOM 1545 C2 CuN X 1 26.720 22.798 73.870 0.00 0.00

ATOM 1546 C2 CuN X 1 4.950 2.418 26.190 0.00 0.00

ATOM 1547 C2 CuN X 1 4.950 2.418 42.110 0.00 0.00

ATOM	1548	C2	CuN	X	1	4.950	2.418	58.020	0.00	0.00
ATOM	1549	C2	CuN	X	1	4.950	2.418	73.940	0.00	0.00
ATOM	1550	C2	CuN	X	1	4.950	13.318	26.190	0.00	0.00
ATOM	1551	C2	CuN	X	1	4.950	13.318	42.110	0.00	0.00
ATOM	1552	C2	CuN	X	1	4.950	13.318	58.020	0.00	0.00
ATOM	1553	C2	CuN	X	1	4.950	13.318	73.940	0.00	0.00
ATOM	1554	C2	CuN	X	1	4.950	24.208	26.190	0.00	0.00
ATOM	1555	C2	CuN	X	1	4.950	24.208	42.110	0.00	0.00
ATOM	1556	C2	CuN	X	1	4.950	24.208	58.020	0.00	0.00
ATOM	1557	C2	CuN	X	1	4.950	24.208	73.940	0.00	0.00
ATOM	1558	C2	CuN	X	1	15.840	2.418	26.190	0.00	0.00
ATOM	1559	C2	CuN	X	1	15.840	2.418	42.110	0.00	0.00
ATOM	1560	C2	CuN	X	1	15.840	2.418	58.020	0.00	0.00
ATOM	1561	C2	CuN	X	1	15.840	2.418	73.940	0.00	0.00
ATOM	1562	C2	CuN	X	1	15.840	13.318	26.190	0.00	0.00
ATOM	1563	C2	CuN	X	1	15.840	13.318	42.110	0.00	0.00
ATOM	1564	C2	CuN	X	1	15.840	13.318	58.020	0.00	0.00
ATOM	1565	C2	CuN	X	1	15.840	13.318	73.940	0.00	0.00
ATOM	1566	C2	CuN	X	1	15.840	24.208	26.190	0.00	0.00
ATOM	1567	C2	CuN	X	1	15.840	24.208	42.110	0.00	0.00
ATOM	1568	C2	CuN	X	1	15.840	24.208	58.020	0.00	0.00
ATOM	1569	C2	CuN	X	1	15.840	24.208	73.940	0.00	0.00
ATOM	1570	C2	CuN	X	1	26.740	2.418	26.190	0.00	0.00
ATOM	1571	C2	CuN	X	1	26.740	2.418	42.110	0.00	0.00
ATOM	1572	C2	CuN	X	1	26.740	2.418	58.020	0.00	0.00
ATOM	1573	C2	CuN	X	1	26.740	2.418	73.940	0.00	0.00
ATOM	1574	C2	CuN	X	1	26.740	13.318	26.190	0.00	0.00
ATOM	1575	C2	CuN	X	1	26.740	13.318	42.110	0.00	0.00
ATOM	1576	C2	CuN	X	1	26.740	13.318	58.020	0.00	0.00
ATOM	1577	C2	CuN	X	1	26.740	13.318	73.940	0.00	0.00

ATOM 1578 C2 CuN X 1 26.740 24.208 26.190 0.00 0.00

ATOM 1579 C2 CuN X 1 26.740 24.208 42.110 0.00 0.00

ATOM 1580 C2 CuN X 1 26.740 24.208 58.020 0.00 0.00

ATOM 1581 C2 CuN X 1 26.740 24.208 73.940 0.00 0.00

ATOM 1582 H1 CuN X 1 1.560 3.018 26.080 0.00 0.00

ATOM 1583 H1 CuN X 1 1.560 3.018 41.990 0.00 0.00

ATOM 1584 H1 CuN X 1 1.560 3.018 57.900 0.00 0.00

ATOM 1585 H1 CuN X 1 1.560 3.018 73.820 0.00 0.00

ATOM 1586 H1 CuN X 1 1.560 13.908 26.080 0.00 0.00

ATOM 1587 H1 CuN X 1 1.560 13.908 41.990 0.00 0.00

ATOM 1588 H1 CuN X 1 1.560 13.908 57.900 0.00 0.00

ATOM 1589 H1 CuN X 1 1.560 13.908 73.820 0.00 0.00

ATOM 1590 H1 CuN X 1 1.560 24.798 26.080 0.00 0.00

ATOM 1591 H1 CuN X 1 1.560 24.798 41.990 0.00 0.00

ATOM 1592 H1 CuN X 1 1.560 24.798 57.900 0.00 0.00

ATOM 1593 H1 CuN X 1 1.560 24.798 73.820 0.00 0.00

ATOM 1594 H1 CuN X 1 12.450 3.018 26.080 0.00 0.00

ATOM 1595 H1 CuN X 1 12.450 3.018 41.990 0.00 0.00

ATOM 1596 H1 CuN X 1 12.450 3.018 57.900 0.00 0.00

ATOM 1597 H1 CuN X 1 12.450 3.018 73.820 0.00 0.00

ATOM 1598 H1 CuN X 1 12.450 13.908 26.080 0.00 0.00

ATOM 1599 H1 CuN X 1 12.450 13.908 41.990 0.00 0.00

ATOM 1600 H1 CuN X 1 12.450 13.908 57.900 0.00 0.00

ATOM 1601 H1 CuN X 1 12.450 13.908 73.820 0.00 0.00

ATOM 1602 H1 CuN X 1 12.450 24.798 26.080 0.00 0.00

ATOM 1603 H1 CuN X 1 12.450 24.798 41.990 0.00 0.00

ATOM 1604 H1 CuN X 1 12.450 24.798 57.900 0.00 0.00

ATOM 1605 H1 CuN X 1 12.450 24.798 73.820 0.00 0.00

ATOM 1606 H1 CuN X 1 23.340 3.018 26.080 0.00 0.00

ATOM 1607 H1 CuN X 1 23.340 3.018 41.990 0.00 0.00

ATOM	1608	H1	CuN	X	1	23.340	3.018	57.900	0.00	0.00
ATOM	1609	H1	CuN	X	1	23.340	3.018	73.820	0.00	0.00
ATOM	1610	H1	CuN	X	1	23.340	13.908	26.080	0.00	0.00
ATOM	1611	H1	CuN	X	1	23.340	13.908	41.990	0.00	0.00
ATOM	1612	H1	CuN	X	1	23.340	13.908	57.900	0.00	0.00
ATOM	1613	H1	CuN	X	1	23.340	13.908	73.820	0.00	0.00
ATOM	1614	H1	CuN	X	1	23.340	24.798	26.080	0.00	0.00
ATOM	1615	H1	CuN	X	1	23.340	24.798	41.990	0.00	0.00
ATOM	1616	H1	CuN	X	1	23.340	24.798	57.900	0.00	0.00
ATOM	1617	H1	CuN	X	1	23.340	24.798	73.820	0.00	0.00
ATOM	1618	H1	CuN	X	1	1.530	0.488	26.060	0.00	0.00
ATOM	1619	H1	CuN	X	1	1.530	0.488	41.970	0.00	0.00
ATOM	1620	H1	CuN	X	1	1.530	0.488	57.890	0.00	0.00
ATOM	1621	H1	CuN	X	1	1.530	0.488	73.800	0.00	0.00
ATOM	1622	H1	CuN	X	1	1.530	11.388	26.060	0.00	0.00
ATOM	1623	H1	CuN	X	1	1.530	11.388	41.970	0.00	0.00
ATOM	1624	H1	CuN	X	1	1.530	11.388	57.890	0.00	0.00
ATOM	1625	H1	CuN	X	1	1.530	11.388	73.800	0.00	0.00
ATOM	1626	H1	CuN	X	1	1.530	22.278	26.060	0.00	0.00
ATOM	1627	H1	CuN	X	1	1.530	22.278	41.970	0.00	0.00
ATOM	1628	H1	CuN	X	1	1.530	22.278	57.890	0.00	0.00
ATOM	1629	H1	CuN	X	1	1.530	22.278	73.800	0.00	0.00
ATOM	1630	H1	CuN	X	1	12.420	0.488	26.060	0.00	0.00
ATOM	1631	H1	CuN	X	1	12.420	0.488	41.970	0.00	0.00
ATOM	1632	H1	CuN	X	1	12.420	0.488	57.890	0.00	0.00
ATOM	1633	H1	CuN	X	1	12.420	0.488	73.800	0.00	0.00
ATOM	1634	H1	CuN	X	1	12.420	11.388	26.060	0.00	0.00
ATOM	1635	H1	CuN	X	1	12.420	11.388	41.970	0.00	0.00
ATOM	1636	H1	CuN	X	1	12.420	11.388	57.890	0.00	0.00
ATOM	1637	H1	CuN	X	1	12.420	11.388	73.800	0.00	0.00

```
ATOM   1638  H1  CuN X   1      12.420  22.278  26.060  0.00  0.00
ATOM   1639  H1  CuN X   1      12.420  22.278  41.970  0.00  0.00
ATOM   1640  H1  CuN X   1      12.420  22.278  57.890  0.00  0.00
ATOM   1641  H1  CuN X   1      12.420  22.278  73.800  0.00  0.00
ATOM   1642  H1  CuN X   1      23.310   0.488  26.060  0.00  0.00
ATOM   1643  H1  CuN X   1      23.310   0.488  41.970  0.00  0.00
ATOM   1644  H1  CuN X   1      23.310   0.488  57.890  0.00  0.00
ATOM   1645  H1  CuN X   1      23.310   0.488  73.800  0.00  0.00
ATOM   1646  H1  CuN X   1      23.310  11.388  26.060  0.00  0.00
ATOM   1647  H1  CuN X   1      23.310  11.388  41.970  0.00  0.00
ATOM   1648  H1  CuN X   1      23.310  11.388  57.890  0.00  0.00
ATOM   1649  H1  CuN X   1      23.310  11.388  73.800  0.00  0.00
ATOM   1650  H1  CuN X   1      23.310  22.278  26.060  0.00  0.00
ATOM   1651  H1  CuN X   1      23.310  22.278  41.970  0.00  0.00
ATOM   1652  H1  CuN X   1      23.310  22.278  57.890  0.00  0.00
ATOM   1653  H1  CuN X   1      23.310  22.278  73.800  0.00  0.00
ATOM   1654  H1  CuN X   1       5.880   0.448  26.130  0.00  0.00
ATOM   1655  H1  CuN X   1       5.880   0.448  42.040  0.00  0.00
ATOM   1656  H1  CuN X   1       5.880   0.448  57.950  0.00  0.00
ATOM   1657  H1  CuN X   1       5.880   0.448  73.870  0.00  0.00
ATOM   1658  H1  CuN X   1       5.880  11.338  26.130  0.00  0.00
ATOM   1659  H1  CuN X   1       5.880  11.338  42.040  0.00  0.00
ATOM   1660  H1  CuN X   1       5.880  11.338  57.950  0.00  0.00
ATOM   1661  H1  CuN X   1       5.880  11.338  73.870  0.00  0.00
ATOM   1662  H1  CuN X   1       5.880  22.228  26.130  0.00  0.00
ATOM   1663  H1  CuN X   1       5.880  22.228  42.040  0.00  0.00
ATOM   1664  H1  CuN X   1       5.880  22.228  57.950  0.00  0.00
ATOM   1665  H1  CuN X   1       5.880  22.228  73.870  0.00  0.00
ATOM   1666  H1  CuN X   1      16.770   0.448  26.130  0.00  0.00
ATOM   1667  H1  CuN X   1      16.770   0.448  42.040  0.00  0.00
```

ATOM	1668	H1	CuN	X	1	16.770	0.448	57.950	0.00	0.00
ATOM	1669	H1	CuN	X	1	16.770	0.448	73.870	0.00	0.00
ATOM	1670	H1	CuN	X	1	16.770	11.338	26.130	0.00	0.00
ATOM	1671	H1	CuN	X	1	16.770	11.338	42.040	0.00	0.00
ATOM	1672	H1	CuN	X	1	16.770	11.338	57.950	0.00	0.00
ATOM	1673	H1	CuN	X	1	16.770	11.338	73.870	0.00	0.00
ATOM	1674	H1	CuN	X	1	16.770	22.228	26.130	0.00	0.00
ATOM	1675	H1	CuN	X	1	16.770	22.228	42.040	0.00	0.00
ATOM	1676	H1	CuN	X	1	16.770	22.228	57.950	0.00	0.00
ATOM	1677	H1	CuN	X	1	16.770	22.228	73.870	0.00	0.00
ATOM	1678	H1	CuN	X	1	27.660	0.448	26.130	0.00	0.00
ATOM	1679	H1	CuN	X	1	27.660	0.448	42.040	0.00	0.00
ATOM	1680	H1	CuN	X	1	27.660	0.448	57.950	0.00	0.00
ATOM	1681	H1	CuN	X	1	27.660	0.448	73.870	0.00	0.00
ATOM	1682	H1	CuN	X	1	27.660	11.338	26.130	0.00	0.00
ATOM	1683	H1	CuN	X	1	27.660	11.338	42.040	0.00	0.00
ATOM	1684	H1	CuN	X	1	27.660	11.338	57.950	0.00	0.00
ATOM	1685	H1	CuN	X	1	27.660	11.338	73.870	0.00	0.00
ATOM	1686	H1	CuN	X	1	27.660	22.228	26.130	0.00	0.00
ATOM	1687	H1	CuN	X	1	27.660	22.228	42.040	0.00	0.00
ATOM	1688	H1	CuN	X	1	27.660	22.228	57.950	0.00	0.00
ATOM	1689	H1	CuN	X	1	27.660	22.228	73.870	0.00	0.00
ATOM	1690	H1	CuN	X	1	5.910	2.968	26.250	0.00	0.00
ATOM	1691	H1	CuN	X	1	5.910	2.968	42.160	0.00	0.00
ATOM	1692	H1	CuN	X	1	5.910	2.968	58.080	0.00	0.00
ATOM	1693	H1	CuN	X	1	5.910	2.968	73.990	0.00	0.00
ATOM	1694	H1	CuN	X	1	5.910	13.858	26.250	0.00	0.00
ATOM	1695	H1	CuN	X	1	5.910	13.858	42.160	0.00	0.00
ATOM	1696	H1	CuN	X	1	5.910	13.858	58.080	0.00	0.00
ATOM	1697	H1	CuN	X	1	5.910	13.858	73.990	0.00	0.00

ATOM	1698	H1	CuN	X	1	5.910	24.748	26.250	0.00	0.00
ATOM	1699	H1	CuN	X	1	5.910	24.748	42.160	0.00	0.00
ATOM	1700	H1	CuN	X	1	5.910	24.748	58.080	0.00	0.00
ATOM	1701	H1	CuN	X	1	5.910	24.748	73.990	0.00	0.00
ATOM	1702	H1	CuN	X	1	16.800	2.968	26.250	0.00	0.00
ATOM	1703	H1	CuN	X	1	16.800	2.968	42.160	0.00	0.00
ATOM	1704	H1	CuN	X	1	16.800	2.968	58.080	0.00	0.00
ATOM	1705	H1	CuN	X	1	16.800	2.968	73.990	0.00	0.00
ATOM	1706	H1	CuN	X	1	16.800	13.858	26.250	0.00	0.00
ATOM	1707	H1	CuN	X	1	16.800	13.858	42.160	0.00	0.00
ATOM	1708	H1	CuN	X	1	16.800	13.858	58.080	0.00	0.00
ATOM	1709	H1	CuN	X	1	16.800	13.858	73.990	0.00	0.00
ATOM	1710	H1	CuN	X	1	16.800	24.748	26.250	0.00	0.00
ATOM	1711	H1	CuN	X	1	16.800	24.748	42.160	0.00	0.00
ATOM	1712	H1	CuN	X	1	16.800	24.748	58.080	0.00	0.00
ATOM	1713	H1	CuN	X	1	16.800	24.748	73.990	0.00	0.00
ATOM	1714	H1	CuN	X	1	27.690	2.968	26.250	0.00	0.00
ATOM	1715	H1	CuN	X	1	27.690	2.968	42.160	0.00	0.00
ATOM	1716	H1	CuN	X	1	27.690	2.968	58.080	0.00	0.00
ATOM	1717	H1	CuN	X	1	27.690	2.968	73.990	0.00	0.00
ATOM	1718	H1	CuN	X	1	27.690	13.858	26.250	0.00	0.00
ATOM	1719	H1	CuN	X	1	27.690	13.858	42.160	0.00	0.00
ATOM	1720	H1	CuN	X	1	27.690	13.858	58.080	0.00	0.00
ATOM	1721	H1	CuN	X	1	27.690	13.858	73.990	0.00	0.00
ATOM	1722	H1	CuN	X	1	27.690	24.748	26.250	0.00	0.00
ATOM	1723	H1	CuN	X	1	27.690	24.748	42.160	0.00	0.00
ATOM	1724	H1	CuN	X	1	27.690	24.748	58.080	0.00	0.00
ATOM	1725	H1	CuN	X	1	27.690	24.748	73.990	0.00	0.00
ATOM	1726	C1	CuN	X	1	3.740	4.638	26.180	0.00	0.00
ATOM	1727	C1	CuN	X	1	3.740	4.638	42.100	0.00	0.00

```
ATOM   1728  C1  CuN X   1       3.740   4.638  58.010  0.00  0.00
ATOM   1729  C1  CuN X   1       3.740   4.638  73.930  0.00  0.00
ATOM   1730  C1  CuN X   1       3.740  15.528  26.180  0.00  0.00
ATOM   1731  C1  CuN X   1       3.740  15.528  42.100  0.00  0.00
ATOM   1732  C1  CuN X   1       3.740  15.528  58.010  0.00  0.00
ATOM   1733  C1  CuN X   1       3.740  15.528  73.930  0.00  0.00
ATOM   1734  C1  CuN X   1       3.740  26.428  26.180  0.00  0.00
ATOM   1735  C1  CuN X   1       3.740  26.428  42.100  0.00  0.00
ATOM   1736  C1  CuN X   1       3.740  26.428  58.010  0.00  0.00
ATOM   1737  C1  CuN X   1       3.740  26.428  73.930  0.00  0.00
ATOM   1738  C1  CuN X   1      14.630   4.638  26.180  0.00  0.00
ATOM   1739  C1  CuN X   1      14.630   4.638  42.100  0.00  0.00
ATOM   1740  C1  CuN X   1      14.630   4.638  58.010  0.00  0.00
ATOM   1741  C1  CuN X   1      14.630   4.638  73.930  0.00  0.00
ATOM   1742  C1  CuN X   1      14.630  15.528  26.180  0.00  0.00
ATOM   1743  C1  CuN X   1      14.630  15.528  42.100  0.00  0.00
ATOM   1744  C1  CuN X   1      14.630  15.528  58.010  0.00  0.00
ATOM   1745  C1  CuN X   1      14.630  15.528  73.930  0.00  0.00
ATOM   1746  C1  CuN X   1      14.630  26.428  26.180  0.00  0.00
ATOM   1747  C1  CuN X   1      14.630  26.428  42.100  0.00  0.00
ATOM   1748  C1  CuN X   1      14.630  26.428  58.010  0.00  0.00
ATOM   1749  C1  CuN X   1      14.630  26.428  73.930  0.00  0.00
ATOM   1750  C1  CuN X   1      25.520   4.638  26.180  0.00  0.00
ATOM   1751  C1  CuN X   1      25.520   4.638  42.100  0.00  0.00
ATOM   1752  C1  CuN X   1      25.520   4.638  58.010  0.00  0.00
ATOM   1753  C1  CuN X   1      25.520   4.638  73.930  0.00  0.00
ATOM   1754  C1  CuN X   1      25.520  15.528  26.180  0.00  0.00
ATOM   1755  C1  CuN X   1      25.520  15.528  42.100  0.00  0.00
ATOM   1756  C1  CuN X   1      25.520  15.528  58.010  0.00  0.00
ATOM   1757  C1  CuN X   1      25.520  15.528  73.930  0.00  0.00
```

ATOM 1758 C1 CuN X 1 25.520 26.428 26.180 0.00 0.00

ATOM 1759 C1 CuN X 1 25.520 26.428 42.100 0.00 0.00

ATOM 1760 C1 CuN X 1 25.520 26.428 58.010 0.00 0.00

ATOM 1761 C1 CuN X 1 25.520 26.428 73.930 0.00 0.00

ATOM 1762 O CuN X 1 1.730 7.488 25.020 0.00 0.00

ATOM 1763 O CuN X 1 1.740 6.858 27.230 0.00 0.00

ATOM 1764 O CuN X 1 1.730 7.488 40.940 0.00 0.00

ATOM 1765 O CuN X 1 1.740 6.858 43.140 0.00 0.00

ATOM 1766 O CuN X 1 1.730 7.488 56.850 0.00 0.00

ATOM 1767 O CuN X 1 1.740 6.858 59.050 0.00 0.00

ATOM 1768 O CuN X 1 1.730 7.488 72.760 0.00 0.00

ATOM 1769 O CuN X 1 1.740 6.858 74.970 0.00 0.00

ATOM 1770 O CuN X 1 1.730 18.378 25.020 0.00 0.00

ATOM 1771 O CuN X 1 1.740 17.748 27.230 0.00 0.00

ATOM 1772 O CuN X 1 1.730 18.378 40.940 0.00 0.00

ATOM 1773 O CuN X 1 1.740 17.748 43.140 0.00 0.00

ATOM 1774 O CuN X 1 1.730 18.378 56.850 0.00 0.00

ATOM 1775 O CuN X 1 1.740 17.748 59.050 0.00 0.00

ATOM 1776 O CuN X 1 1.730 18.378 72.760 0.00 0.00

ATOM 1777 O CuN X 1 1.740 17.748 74.970 0.00 0.00

ATOM 1778 O CuN X 1 1.730 29.268 25.020 0.00 0.00

ATOM 1779 O CuN X 1 1.740 28.638 27.230 0.00 0.00

ATOM 1780 O CuN X 1 1.730 29.268 40.940 0.00 0.00

ATOM 1781 O CuN X 1 1.740 28.638 43.140 0.00 0.00

ATOM 1782 O CuN X 1 1.730 29.268 56.850 0.00 0.00

ATOM 1783 O CuN X 1 1.740 28.638 59.050 0.00 0.00

ATOM 1784 O CuN X 1 1.730 29.268 72.760 0.00 0.00

ATOM 1785 O CuN X 1 1.740 28.638 74.970 0.00 0.00

ATOM 1786 H3 CuN X 1 3.270 31.398 32.030 0.00 0.00

ATOM 1787 H3 CuN X 1 3.270 31.398 47.940 0.00 0.00

ATOM	1788	H3	CuN	X	1	3.270	31.398	63.850	0.00	0.00
ATOM	1789	H3	CuN	X	1	14.160	31.398	32.030	0.00	0.00
ATOM	1790	H3	CuN	X	1	14.160	31.398	47.940	0.00	0.00
ATOM	1791	H3	CuN	X	1	14.160	31.398	63.850	0.00	0.00
ATOM	1792	H3	CuN	X	1	25.050	31.398	32.030	0.00	0.00
ATOM	1793	H3	CuN	X	1	25.050	31.398	47.940	0.00	0.00
ATOM	1794	H3	CuN	X	1	25.050	31.398	63.850	0.00	0.00
ATOM	1795	O	CuN	X	1	4.000	30.948	24.960	0.00	0.00
ATOM	1796	O	CuN	X	1	3.370	30.948	27.160	0.00	0.00
ATOM	1797	O	CuN	X	1	4.000	30.948	40.880	0.00	0.00
ATOM	1798	O	CuN	X	1	3.370	30.948	43.080	0.00	0.00
ATOM	1799	O	CuN	X	1	4.000	30.948	56.790	0.00	0.00
ATOM	1800	O	CuN	X	1	3.370	30.948	58.990	0.00	0.00
ATOM	1801	O	CuN	X	1	4.000	30.948	72.700	0.00	0.00
ATOM	1802	O	CuN	X	1	3.370	30.948	74.910	0.00	0.00
ATOM	1803	O	CuN	X	1	14.900	30.948	24.960	0.00	0.00
ATOM	1804	O	CuN	X	1	14.260	30.948	27.160	0.00	0.00
ATOM	1805	O	CuN	X	1	14.900	30.948	40.880	0.00	0.00
ATOM	1806	O	CuN	X	1	14.260	30.948	43.080	0.00	0.00
ATOM	1807	O	CuN	X	1	14.900	30.948	56.790	0.00	0.00
ATOM	1808	O	CuN	X	1	14.260	30.948	58.990	0.00	0.00
ATOM	1809	O	CuN	X	1	14.900	30.948	72.700	0.00	0.00
ATOM	1810	O	CuN	X	1	14.260	30.948	74.910	0.00	0.00
ATOM	1811	O	CuN	X	1	25.790	30.948	24.960	0.00	0.00
ATOM	1812	O	CuN	X	1	25.150	30.948	27.160	0.00	0.00
ATOM	1813	O	CuN	X	1	25.790	30.948	40.880	0.00	0.00
ATOM	1814	O	CuN	X	1	25.150	30.948	43.080	0.00	0.00
ATOM	1815	O	CuN	X	1	25.790	30.948	56.790	0.00	0.00
ATOM	1816	O	CuN	X	1	25.150	30.948	58.990	0.00	0.00
ATOM	1817	O	CuN	X	1	25.790	30.948	72.700	0.00	0.00

ATOM   1818 O   CuN X   1   25.150 30.948 74.910 0.00 0.00

ATOM   1819 CB MEOHX   2   19.800 14.368 42.180 0.00 0.00

ATOM   1820 OG MEOHX   2   20.280 13.218 41.510 0.00 0.00

ATOM   1821 HG1 MEOHX   2   21.230 13.248 41.540 0.00 0.00

ATOM   1822 HB1 MEOHX   2   20.230 14.408 43.210 0.00 0.00

ATOM   1823 HB2 MEOHX   2   18.690 14.308 42.160 0.00 0.00

ATOM   1824 HB3 MEOHX   2   20.060 15.368 41.760 0.00 0.00

Coordinates for PL$_{C=C}$ for the calculation of binding energy for methanol

CRYST1   32.673   32.673 100.000 90.00 90.00 90.00 P 1

ATOM   1 O   CuC X   1   5.720   7.460 26.590 0.00 0.00

ATOM   2 O   CuC X   1   5.720   7.460 42.900 0.00 0.00

ATOM   3 O   CuC X   1   5.720   7.460 59.200 0.00 0.00

ATOM   4 O   CuC X   1   5.720   7.460 75.500 0.00 0.00

ATOM   5 O   CuC X   1   5.720 18.350 26.590 0.00 0.00

ATOM   6 O   CuC X   1   5.720 18.350 42.900 0.00 0.00

ATOM   7 O   CuC X   1   5.720 18.350 59.200 0.00 0.00

ATOM   8 O   CuC X   1   5.720 18.350 75.500 0.00 0.00

ATOM   9 O   CuC X   1   5.720 29.240 26.590 0.00 0.00

ATOM   10 O   CuC X   1   5.720 29.240 42.900 0.00 0.00

ATOM   11 O   CuC X   1   5.720 29.240 59.200 0.00 0.00

ATOM   12 O   CuC X   1   5.720 29.240 75.500 0.00 0.00

ATOM   13 O   CuC X   1   16.610   7.460 26.590 0.00 0.00

ATOM   14 O   CuC X   1   16.610   7.460 42.900 0.00 0.00

ATOM   15 O   CuC X   1   16.610   7.460 59.200 0.00 0.00

ATOM   16 O   CuC X   1   16.610   7.460 75.500 0.00 0.00

ATOM	17	O	CuC	X	1	16.610	18.350	26.590	0.00	0.00
ATOM	18	O	CuC	X	1	16.610	18.350	42.900	0.00	0.00
ATOM	19	O	CuC	X	1	16.610	18.350	59.200	0.00	0.00
ATOM	20	O	CuC	X	1	16.610	18.350	75.500	0.00	0.00
ATOM	21	O	CuC	X	1	16.610	29.240	26.590	0.00	0.00
ATOM	22	O	CuC	X	1	16.610	29.240	42.900	0.00	0.00
ATOM	23	O	CuC	X	1	16.610	29.240	59.200	0.00	0.00
ATOM	24	O	CuC	X	1	16.610	29.240	75.500	0.00	0.00
ATOM	25	O	CuC	X	1	27.500	7.460	26.590	0.00	0.00
ATOM	26	O	CuC	X	1	27.500	7.460	42.900	0.00	0.00
ATOM	27	O	CuC	X	1	27.500	7.460	59.200	0.00	0.00
ATOM	28	O	CuC	X	1	27.500	7.460	75.500	0.00	0.00
ATOM	29	O	CuC	X	1	27.500	18.350	26.590	0.00	0.00
ATOM	30	O	CuC	X	1	27.500	18.350	42.900	0.00	0.00
ATOM	31	O	CuC	X	1	27.500	18.350	59.200	0.00	0.00
ATOM	32	O	CuC	X	1	27.500	18.350	75.500	0.00	0.00
ATOM	33	O	CuC	X	1	27.500	29.240	26.590	0.00	0.00
ATOM	34	O	CuC	X	1	27.500	29.240	42.900	0.00	0.00
ATOM	35	O	CuC	X	1	27.500	29.240	59.200	0.00	0.00
ATOM	36	O	CuC	X	1	27.500	29.240	75.500	0.00	0.00
ATOM	37	O	CuC	X	1	5.700	6.790	24.400	0.00	0.00
ATOM	38	O	CuC	X	1	5.700	6.790	40.710	0.00	0.00
ATOM	39	O	CuC	X	1	5.700	6.790	57.010	0.00	0.00
ATOM	40	O	CuC	X	1	5.700	6.790	73.310	0.00	0.00

ATOM	41	O	CuC	X	1	5.700	17.680	24.400	0.00	0.00
ATOM	42	O	CuC	X	1	5.700	17.680	40.710	0.00	0.00
ATOM	43	O	CuC	X	1	5.700	17.680	57.010	0.00	0.00
ATOM	44	O	CuC	X	1	5.700	17.680	73.310	0.00	0.00
ATOM	45	O	CuC	X	1	5.700	28.570	24.400	0.00	0.00
ATOM	46	O	CuC	X	1	5.700	28.570	40.710	0.00	0.00
ATOM	47	O	CuC	X	1	5.700	28.570	57.010	0.00	0.00
ATOM	48	O	CuC	X	1	5.700	28.570	73.310	0.00	0.00
ATOM	49	O	CuC	X	1	16.590	6.790	24.400	0.00	0.00
ATOM	50	O	CuC	X	1	16.590	6.790	40.710	0.00	0.00
ATOM	51	O	CuC	X	1	16.590	6.790	57.010	0.00	0.00
ATOM	52	O	CuC	X	1	16.590	6.790	73.310	0.00	0.00
ATOM	53	O	CuC	X	1	16.590	17.680	24.400	0.00	0.00
ATOM	54	O	CuC	X	1	16.590	17.680	40.710	0.00	0.00
ATOM	55	O	CuC	X	1	16.590	17.680	57.010	0.00	0.00
ATOM	56	O	CuC	X	1	16.590	17.680	73.310	0.00	0.00
ATOM	57	O	CuC	X	1	16.590	28.570	24.400	0.00	0.00
ATOM	58	O	CuC	X	1	16.590	28.570	40.710	0.00	0.00
ATOM	59	O	CuC	X	1	16.590	28.570	57.010	0.00	0.00
ATOM	60	O	CuC	X	1	16.590	28.570	73.310	0.00	0.00
ATOM	61	O	CuC	X	1	27.480	6.790	24.400	0.00	0.00
ATOM	62	O	CuC	X	1	27.480	6.790	40.710	0.00	0.00
ATOM	63	O	CuC	X	1	27.480	6.790	57.010	0.00	0.00
ATOM	64	O	CuC	X	1	27.480	6.790	73.310	0.00	0.00

ATOM	65	O	CuC	X	1	27.480	17.680	24.400	0.00	0.00
ATOM	66	O	CuC	X	1	27.480	17.680	40.710	0.00	0.00
ATOM	67	O	CuC	X	1	27.480	17.680	57.010	0.00	0.00
ATOM	68	O	CuC	X	1	27.480	17.680	73.310	0.00	0.00
ATOM	69	O	CuC	X	1	27.480	28.570	24.400	0.00	0.00
ATOM	70	O	CuC	X	1	27.480	28.570	40.710	0.00	0.00
ATOM	71	O	CuC	X	1	27.480	28.570	57.010	0.00	0.00
ATOM	72	O	CuC	X	1	27.480	28.570	73.310	0.00	0.00
ATOM	73	O	CuC	X	1	12.670	7.450	24.400	0.00	0.00
ATOM	74	O	CuC	X	1	12.670	7.450	40.710	0.00	0.00
ATOM	75	O	CuC	X	1	12.670	7.450	57.010	0.00	0.00
ATOM	76	O	CuC	X	1	12.670	7.450	73.310	0.00	0.00
ATOM	77	O	CuC	X	1	12.670	18.340	24.400	0.00	0.00
ATOM	78	O	CuC	X	1	12.670	18.340	40.710	0.00	0.00
ATOM	79	O	CuC	X	1	12.670	18.340	57.010	0.00	0.00
ATOM	80	O	CuC	X	1	12.670	18.340	73.310	0.00	0.00
ATOM	81	O	CuC	X	1	12.670	29.230	24.400	0.00	0.00
ATOM	82	O	CuC	X	1	12.670	29.230	40.710	0.00	0.00
ATOM	83	O	CuC	X	1	12.670	29.230	57.010	0.00	0.00
ATOM	84	O	CuC	X	1	12.670	29.230	73.310	0.00	0.00
ATOM	85	O	CuC	X	1	23.570	7.450	24.400	0.00	0.00
ATOM	86	O	CuC	X	1	23.570	7.450	40.710	0.00	0.00
ATOM	87	O	CuC	X	1	23.570	7.450	57.010	0.00	0.00
ATOM	88	O	CuC	X	1	23.570	7.450	73.310	0.00	0.00

ATOM	89	O	CuC	X	1	23.570	18.340	24.400	0.00	0.00
ATOM	90	O	CuC	X	1	23.570	18.340	40.710	0.00	0.00
ATOM	91	O	CuC	X	1	23.570	18.340	57.010	0.00	0.00
ATOM	92	O	CuC	X	1	23.570	18.340	73.310	0.00	0.00
ATOM	93	O	CuC	X	1	23.570	29.230	24.400	0.00	0.00
ATOM	94	O	CuC	X	1	23.570	29.230	40.710	0.00	0.00
ATOM	95	O	CuC	X	1	23.570	29.230	57.010	0.00	0.00
ATOM	96	O	CuC	X	1	23.570	29.230	73.310	0.00	0.00
ATOM	97	O	CuC	X	1	12.660	6.780	26.600	0.00	0.00
ATOM	98	O	CuC	X	1	12.660	6.780	42.900	0.00	0.00
ATOM	99	O	CuC	X	1	12.660	6.780	59.200	0.00	0.00
ATOM	100	O	CuC	X	1	12.660	6.780	75.510	0.00	0.00
ATOM	101	O	CuC	X	1	12.660	17.670	26.600	0.00	0.00
ATOM	102	O	CuC	X	1	12.660	17.670	42.900	0.00	0.00
ATOM	103	O	CuC	X	1	12.660	17.670	59.200	0.00	0.00
ATOM	104	O	CuC	X	1	12.660	17.670	75.510	0.00	0.00
ATOM	105	O	CuC	X	1	12.660	28.560	26.600	0.00	0.00
ATOM	106	O	CuC	X	1	12.660	28.560	42.900	0.00	0.00
ATOM	107	O	CuC	X	1	12.660	28.560	59.200	0.00	0.00
ATOM	108	O	CuC	X	1	12.660	28.560	75.510	0.00	0.00
ATOM	109	O	CuC	X	1	23.550	6.780	26.600	0.00	0.00
ATOM	110	O	CuC	X	1	23.550	6.780	42.900	0.00	0.00
ATOM	111	O	CuC	X	1	23.550	6.780	59.200	0.00	0.00
ATOM	112	O	CuC	X	1	23.550	6.780	75.510	0.00	0.00

ATOM 113 O CuC X 1 23.550 17.670 26.600 0.00 0.00

ATOM 114 O CuC X 1 23.550 17.670 42.900 0.00 0.00

ATOM 115 O CuC X 1 23.550 17.670 59.200 0.00 0.00

ATOM 116 O CuC X 1 23.550 17.670 75.510 0.00 0.00

ATOM 117 O CuC X 1 23.550 28.560 26.600 0.00 0.00

ATOM 118 O CuC X 1 23.550 28.560 42.900 0.00 0.00

ATOM 119 O CuC X 1 23.550 28.560 59.200 0.00 0.00

ATOM 120 O CuC X 1 23.550 28.560 75.510 0.00 0.00

ATOM 121 Cu CuC X 1 3.740 7.140 26.860 0.00 0.00

ATOM 122 Cu CuC X 1 3.740 7.140 43.160 0.00 0.00

ATOM 123 Cu CuC X 1 3.740 7.140 59.470 0.00 0.00

ATOM 124 Cu CuC X 1 3.740 7.140 75.770 0.00 0.00

ATOM 125 Cu CuC X 1 3.740 18.040 26.860 0.00 0.00

ATOM 126 Cu CuC X 1 3.740 18.040 43.160 0.00 0.00

ATOM 127 Cu CuC X 1 3.740 18.040 59.470 0.00 0.00

ATOM 128 Cu CuC X 1 3.740 18.040 75.770 0.00 0.00

ATOM 129 Cu CuC X 1 3.740 28.930 26.860 0.00 0.00

ATOM 130 Cu CuC X 1 3.740 28.930 43.160 0.00 0.00

ATOM 131 Cu CuC X 1 3.740 28.930 59.470 0.00 0.00

ATOM 132 Cu CuC X 1 3.740 28.930 75.770 0.00 0.00

ATOM 133 Cu CuC X 1 14.630 7.140 26.860 0.00 0.00

ATOM 134 Cu CuC X 1 14.630 7.140 43.160 0.00 0.00

ATOM 135 Cu CuC X 1 14.630 7.140 59.470 0.00 0.00

ATOM 136 Cu CuC X 1 14.630 7.140 75.770 0.00 0.00

ATOM 137 Cu CuC X 1 14.630 18.040 26.860 0.00 0.00

ATOM 138 Cu CuC X 1 14.630 18.040 43.160 0.00 0.00

ATOM 139 Cu CuC X 1 14.630 18.040 59.470 0.00 0.00

ATOM 140 Cu CuC X 1 14.630 18.040 75.770 0.00 0.00

ATOM 141 Cu CuC X 1 14.630 28.930 26.860 0.00 0.00

ATOM 142 Cu CuC X 1 14.630 28.930 43.160 0.00 0.00

ATOM 143 Cu CuC X 1 14.630 28.930 59.470 0.00 0.00

ATOM 144 Cu CuC X 1 14.630 28.930 75.770 0.00 0.00

ATOM 145 Cu CuC X 1 25.520 7.140 26.860 0.00 0.00

ATOM 146 Cu CuC X 1 25.520 7.140 43.160 0.00 0.00

ATOM 147 Cu CuC X 1 25.520 7.140 59.470 0.00 0.00

ATOM 148 Cu CuC X 1 25.520 7.140 75.770 0.00 0.00

ATOM 149 Cu CuC X 1 25.520 18.040 26.860 0.00 0.00

ATOM 150 Cu CuC X 1 25.520 18.040 43.160 0.00 0.00

ATOM 151 Cu CuC X 1 25.520 18.040 59.470 0.00 0.00

ATOM 152 Cu CuC X 1 25.520 18.040 75.770 0.00 0.00

ATOM 153 Cu CuC X 1 25.520 28.930 26.860 0.00 0.00

ATOM 154 Cu CuC X 1 25.520 28.930 43.160 0.00 0.00

ATOM 155 Cu CuC X 1 25.520 28.930 59.470 0.00 0.00

ATOM 156 Cu CuC X 1 25.520 28.930 75.770 0.00 0.00

ATOM 157 Cu CuC X 1 3.730 7.120 24.240 0.00 0.00

ATOM 158 Cu CuC X 1 3.730 7.120 40.550 0.00 0.00

ATOM 159 Cu CuC X 1 3.730 7.120 56.850 0.00 0.00

ATOM 160 Cu CuC X 1 3.730 7.120 73.150 0.00 0.00

ATOM 161 Cu CuC X 1 3.730 18.010 24.240 0.00 0.00

ATOM 162 Cu CuC X 1 3.730 18.010 40.550 0.00 0.00

ATOM 163 Cu CuC X 1 3.730 18.010 56.850 0.00 0.00

ATOM 164 Cu CuC X 1 3.730 18.010 73.150 0.00 0.00

ATOM 165 Cu CuC X 1 3.730 28.900 24.240 0.00 0.00

ATOM 166 Cu CuC X 1 3.730 28.900 40.550 0.00 0.00

ATOM 167 Cu CuC X 1 3.730 28.900 56.850 0.00 0.00

ATOM 168 Cu CuC X 1 3.730 28.900 73.150 0.00 0.00

ATOM 169 Cu CuC X 1 14.630 7.120 24.240 0.00 0.00

ATOM 170 Cu CuC X 1 14.630 7.120 40.550 0.00 0.00

ATOM 171 Cu CuC X 1 14.630 7.120 56.850 0.00 0.00

ATOM 172 Cu CuC X 1 14.630 7.120 73.150 0.00 0.00

ATOM 173 Cu CuC X 1 14.630 18.010 24.240 0.00 0.00

ATOM 174 Cu CuC X 1 14.630 18.010 40.550 0.00 0.00

ATOM 175 Cu CuC X 1 14.630 18.010 56.850 0.00 0.00

ATOM 176 Cu CuC X 1 14.630 18.010 73.150 0.00 0.00

ATOM 177 Cu CuC X 1 14.630 28.900 24.240 0.00 0.00

ATOM 178 Cu CuC X 1 14.630 28.900 40.550 0.00 0.00

ATOM 179 Cu CuC X 1 14.630 28.900 56.850 0.00 0.00

ATOM 180 Cu CuC X 1 14.630 28.900 73.150 0.00 0.00

ATOM 181 Cu CuC X 1 25.520 7.120 24.240 0.00 0.00

ATOM 182 Cu CuC X 1 25.520 7.120 40.550 0.00 0.00

ATOM 183 Cu CuC X 1 25.520 7.120 56.850 0.00 0.00

ATOM 184 Cu CuC X 1 25.520 7.120 73.150 0.00 0.00

ATOM	185	Cu	CuC	X	1	25.520	18.010	24.240	0.00	0.00
ATOM	186	Cu	CuC	X	1	25.520	18.010	40.550	0.00	0.00
ATOM	187	Cu	CuC	X	1	25.520	18.010	56.850	0.00	0.00
ATOM	188	Cu	CuC	X	1	25.520	18.010	73.150	0.00	0.00
ATOM	189	Cu	CuC	X	1	25.520	28.900	24.240	0.00	0.00
ATOM	190	Cu	CuC	X	1	25.520	28.900	40.550	0.00	0.00
ATOM	191	Cu	CuC	X	1	25.520	28.900	56.850	0.00	0.00
ATOM	192	Cu	CuC	X	1	25.520	28.900	73.150	0.00	0.00
ATOM	193	C1	CuC	X	1	12.100	7.110	25.500	0.00	0.00
ATOM	194	C1	CuC	X	1	12.100	7.110	41.800	0.00	0.00
ATOM	195	C1	CuC	X	1	12.100	7.110	58.100	0.00	0.00
ATOM	196	C1	CuC	X	1	12.100	7.110	74.410	0.00	0.00
ATOM	197	C1	CuC	X	1	12.100	18.000	25.500	0.00	0.00
ATOM	198	C1	CuC	X	1	12.100	18.000	41.800	0.00	0.00
ATOM	199	C1	CuC	X	1	12.100	18.000	58.100	0.00	0.00
ATOM	200	C1	CuC	X	1	12.100	18.000	74.410	0.00	0.00
ATOM	201	C1	CuC	X	1	12.100	28.890	25.500	0.00	0.00
ATOM	202	C1	CuC	X	1	12.100	28.890	41.800	0.00	0.00
ATOM	203	C1	CuC	X	1	12.100	28.890	58.100	0.00	0.00
ATOM	204	C1	CuC	X	1	12.100	28.890	74.410	0.00	0.00
ATOM	205	C1	CuC	X	1	22.990	7.110	25.500	0.00	0.00
ATOM	206	C1	CuC	X	1	22.990	7.110	41.800	0.00	0.00
ATOM	207	C1	CuC	X	1	22.990	7.110	58.100	0.00	0.00
ATOM	208	C1	CuC	X	1	22.990	7.110	74.410	0.00	0.00

ATOM	209	C1	CuC	X	1	22.990	18.000	25.500	0.00	0.00
ATOM	210	C1	CuC	X	1	22.990	18.000	41.800	0.00	0.00
ATOM	211	C1	CuC	X	1	22.990	18.000	58.100	0.00	0.00
ATOM	212	C1	CuC	X	1	22.990	18.000	74.410	0.00	0.00
ATOM	213	C1	CuC	X	1	22.990	28.890	25.500	0.00	0.00
ATOM	214	C1	CuC	X	1	22.990	28.890	41.800	0.00	0.00
ATOM	215	C1	CuC	X	1	22.990	28.890	58.100	0.00	0.00
ATOM	216	C1	CuC	X	1	22.990	28.890	74.410	0.00	0.00
ATOM	217	C1	CuC	X	1	1.210	7.110	25.500	0.00	0.00
ATOM	218	C1	CuC	X	1	1.210	7.110	41.800	0.00	0.00
ATOM	219	C1	CuC	X	1	1.210	7.110	58.100	0.00	0.00
ATOM	220	C1	CuC	X	1	1.210	7.110	74.410	0.00	0.00
ATOM	221	C1	CuC	X	1	1.210	18.000	25.500	0.00	0.00
ATOM	222	C1	CuC	X	1	1.210	18.000	41.800	0.00	0.00
ATOM	223	C1	CuC	X	1	1.210	18.000	58.100	0.00	0.00
ATOM	224	C1	CuC	X	1	1.210	18.000	74.410	0.00	0.00
ATOM	225	C1	CuC	X	1	1.210	28.890	25.500	0.00	0.00
ATOM	226	C1	CuC	X	1	1.210	28.890	41.800	0.00	0.00
ATOM	227	C1	CuC	X	1	1.210	28.890	58.100	0.00	0.00
ATOM	228	C1	CuC	X	1	1.210	28.890	74.410	0.00	0.00
ATOM	229	C2	CuC	X	1	10.590	7.110	25.480	0.00	0.00
ATOM	230	C2	CuC	X	1	10.590	7.110	41.790	0.00	0.00
ATOM	231	C2	CuC	X	1	10.590	7.110	58.090	0.00	0.00
ATOM	232	C2	CuC	X	1	10.590	7.110	74.390	0.00	0.00

ATOM	233	C2	CuC	X	1	10.590	18.000	25.480	0.00	0.00
ATOM	234	C2	CuC	X	1	10.590	18.000	41.790	0.00	0.00
ATOM	235	C2	CuC	X	1	10.590	18.000	58.090	0.00	0.00
ATOM	236	C2	CuC	X	1	10.590	18.000	74.390	0.00	0.00
ATOM	237	C2	CuC	X	1	10.590	28.890	25.480	0.00	0.00
ATOM	238	C2	CuC	X	1	10.590	28.890	41.790	0.00	0.00
ATOM	239	C2	CuC	X	1	10.590	28.890	58.090	0.00	0.00
ATOM	240	C2	CuC	X	1	10.590	28.890	74.390	0.00	0.00
ATOM	241	C2	CuC	X	1	21.480	7.110	25.480	0.00	0.00
ATOM	242	C2	CuC	X	1	21.480	7.110	41.790	0.00	0.00
ATOM	243	C2	CuC	X	1	21.480	7.110	58.090	0.00	0.00
ATOM	244	C2	CuC	X	1	21.480	7.110	74.390	0.00	0.00
ATOM	245	C2	CuC	X	1	21.480	18.000	25.480	0.00	0.00
ATOM	246	C2	CuC	X	1	21.480	18.000	41.790	0.00	0.00
ATOM	247	C2	CuC	X	1	21.480	18.000	58.090	0.00	0.00
ATOM	248	C2	CuC	X	1	21.480	18.000	74.390	0.00	0.00
ATOM	249	C2	CuC	X	1	21.480	28.890	25.480	0.00	0.00
ATOM	250	C2	CuC	X	1	21.480	28.890	41.790	0.00	0.00
ATOM	251	C2	CuC	X	1	21.480	28.890	58.090	0.00	0.00
ATOM	252	C2	CuC	X	1	21.480	28.890	74.390	0.00	0.00
ATOM	253	C2	CuC	X	1	32.370	7.110	25.480	0.00	0.00
ATOM	254	C2	CuC	X	1	32.370	7.110	41.790	0.00	0.00
ATOM	255	C2	CuC	X	1	32.370	7.110	58.090	0.00	0.00
ATOM	256	C2	CuC	X	1	32.370	7.110	74.390	0.00	0.00

ATOM 257 C2 CuC X 1 32.370 18.000 25.480 0.00 0.00

ATOM 258 C2 CuC X 1 32.370 18.000 41.790 0.00 0.00

ATOM 259 C2 CuC X 1 32.370 18.000 58.090 0.00 0.00

ATOM 260 C2 CuC X 1 32.370 18.000 74.390 0.00 0.00

ATOM 261 C2 CuC X 1 32.370 28.890 25.480 0.00 0.00

ATOM 262 C2 CuC X 1 32.370 28.890 41.790 0.00 0.00

ATOM 263 C2 CuC X 1 32.370 28.890 58.090 0.00 0.00

ATOM 264 C2 CuC X 1 32.370 28.890 74.390 0.00 0.00

ATOM 265 C1 CuC X 1 6.280 7.120 25.500 0.00 0.00

ATOM 266 C1 CuC X 1 6.280 7.120 41.800 0.00 0.00

ATOM 267 C1 CuC X 1 6.280 7.120 58.100 0.00 0.00

ATOM 268 C1 CuC X 1 6.280 7.120 74.410 0.00 0.00

ATOM 269 C1 CuC X 1 6.280 18.010 25.500 0.00 0.00

ATOM 270 C1 CuC X 1 6.280 18.010 41.800 0.00 0.00

ATOM 271 C1 CuC X 1 6.280 18.010 58.100 0.00 0.00

ATOM 272 C1 CuC X 1 6.280 18.010 74.410 0.00 0.00

ATOM 273 C1 CuC X 1 6.280 28.910 25.500 0.00 0.00

ATOM 274 C1 CuC X 1 6.280 28.910 41.800 0.00 0.00

ATOM 275 C1 CuC X 1 6.280 28.910 58.100 0.00 0.00

ATOM 276 C1 CuC X 1 6.280 28.910 74.410 0.00 0.00

ATOM 277 C1 CuC X 1 17.170 7.120 25.500 0.00 0.00

ATOM 278 C1 CuC X 1 17.170 7.120 41.800 0.00 0.00

ATOM 279 C1 CuC X 1 17.170 7.120 58.100 0.00 0.00

ATOM 280 C1 CuC X 1 17.170 7.120 74.410 0.00 0.00

ATOM	281	C1	CuC	X	1	17.170	18.010	25.500	0.00	0.00
ATOM	282	C1	CuC	X	1	17.170	18.010	41.800	0.00	0.00
ATOM	283	C1	CuC	X	1	17.170	18.010	58.100	0.00	0.00
ATOM	284	C1	CuC	X	1	17.170	18.010	74.410	0.00	0.00
ATOM	285	C1	CuC	X	1	17.170	28.910	25.500	0.00	0.00
ATOM	286	C1	CuC	X	1	17.170	28.910	41.800	0.00	0.00
ATOM	287	C1	CuC	X	1	17.170	28.910	58.100	0.00	0.00
ATOM	288	C1	CuC	X	1	17.170	28.910	74.410	0.00	0.00
ATOM	289	C1	CuC	X	1	28.060	7.120	25.500	0.00	0.00
ATOM	290	C1	CuC	X	1	28.060	7.120	41.800	0.00	0.00
ATOM	291	C1	CuC	X	1	28.060	7.120	58.100	0.00	0.00
ATOM	292	C1	CuC	X	1	28.060	7.120	74.410	0.00	0.00
ATOM	293	C1	CuC	X	1	28.060	18.010	25.500	0.00	0.00
ATOM	294	C1	CuC	X	1	28.060	18.010	41.800	0.00	0.00
ATOM	295	C1	CuC	X	1	28.060	18.010	58.100	0.00	0.00
ATOM	296	C1	CuC	X	1	28.060	18.010	74.410	0.00	0.00
ATOM	297	C1	CuC	X	1	28.060	28.910	25.500	0.00	0.00
ATOM	298	C1	CuC	X	1	28.060	28.910	41.800	0.00	0.00
ATOM	299	C1	CuC	X	1	28.060	28.910	58.100	0.00	0.00
ATOM	300	C1	CuC	X	1	28.060	28.910	74.410	0.00	0.00
ATOM	301	C2	CuC	X	1	7.780	7.110	25.490	0.00	0.00
ATOM	302	C2	CuC	X	1	7.780	7.110	41.790	0.00	0.00
ATOM	303	C2	CuC	X	1	7.780	7.110	58.090	0.00	0.00
ATOM	304	C2	CuC	X	1	7.780	7.110	74.390	0.00	0.00

ATOM	305	C2	CuC	X	1	7.780	18.000	25.490	0.00	0.00
ATOM	306	C2	CuC	X	1	7.780	18.000	41.790	0.00	0.00
ATOM	307	C2	CuC	X	1	7.780	18.000	58.090	0.00	0.00
ATOM	308	C2	CuC	X	1	7.780	18.000	74.390	0.00	0.00
ATOM	309	C2	CuC	X	1	7.780	28.890	25.490	0.00	0.00
ATOM	310	C2	CuC	X	1	7.780	28.890	41.790	0.00	0.00
ATOM	311	C2	CuC	X	1	7.780	28.890	58.090	0.00	0.00
ATOM	312	C2	CuC	X	1	7.780	28.890	74.390	0.00	0.00
ATOM	313	C2	CuC	X	1	18.670	7.110	25.490	0.00	0.00
ATOM	314	C2	CuC	X	1	18.670	7.110	41.790	0.00	0.00
ATOM	315	C2	CuC	X	1	18.670	7.110	58.090	0.00	0.00
ATOM	316	C2	CuC	X	1	18.670	7.110	74.390	0.00	0.00
ATOM	317	C2	CuC	X	1	18.670	18.000	25.490	0.00	0.00
ATOM	318	C2	CuC	X	1	18.670	18.000	41.790	0.00	0.00
ATOM	319	C2	CuC	X	1	18.670	18.000	58.090	0.00	0.00
ATOM	320	C2	CuC	X	1	18.670	18.000	74.390	0.00	0.00
ATOM	321	C2	CuC	X	1	18.670	28.890	25.490	0.00	0.00
ATOM	322	C2	CuC	X	1	18.670	28.890	41.790	0.00	0.00
ATOM	323	C2	CuC	X	1	18.670	28.890	58.090	0.00	0.00
ATOM	324	C2	CuC	X	1	18.670	28.890	74.390	0.00	0.00
ATOM	325	C2	CuC	X	1	29.560	7.110	25.490	0.00	0.00
ATOM	326	C2	CuC	X	1	29.560	7.110	41.790	0.00	0.00
ATOM	327	C2	CuC	X	1	29.560	7.110	58.090	0.00	0.00
ATOM	328	C2	CuC	X	1	29.560	7.110	74.390	0.00	0.00

ATOM	329	C2	CuC	X	1	29.560	18.000	25.490	0.00	0.00
ATOM	330	C2	CuC	X	1	29.560	18.000	41.790	0.00	0.00
ATOM	331	C2	CuC	X	1	29.560	18.000	58.090	0.00	0.00
ATOM	332	C2	CuC	X	1	29.560	18.000	74.390	0.00	0.00
ATOM	333	C2	CuC	X	1	29.560	28.890	25.490	0.00	0.00
ATOM	334	C2	CuC	X	1	29.560	28.890	41.790	0.00	0.00
ATOM	335	C2	CuC	X	1	29.560	28.890	58.090	0.00	0.00
ATOM	336	C2	CuC	X	1	29.560	28.890	74.390	0.00	0.00
ATOM	337	C2	CuC	X	1	9.890	7.090	24.260	0.00	0.00
ATOM	338	C2	CuC	X	1	9.890	7.090	40.560	0.00	0.00
ATOM	339	C2	CuC	X	1	9.890	7.090	56.860	0.00	0.00
ATOM	340	C2	CuC	X	1	9.890	7.090	73.170	0.00	0.00
ATOM	341	C2	CuC	X	1	9.890	17.980	24.260	0.00	0.00
ATOM	342	C2	CuC	X	1	9.890	17.980	40.560	0.00	0.00
ATOM	343	C2	CuC	X	1	9.890	17.980	56.860	0.00	0.00
ATOM	344	C2	CuC	X	1	9.890	17.980	73.170	0.00	0.00
ATOM	345	C2	CuC	X	1	9.890	28.880	24.260	0.00	0.00
ATOM	346	C2	CuC	X	1	9.890	28.880	40.560	0.00	0.00
ATOM	347	C2	CuC	X	1	9.890	28.880	56.860	0.00	0.00
ATOM	348	C2	CuC	X	1	9.890	28.880	73.170	0.00	0.00
ATOM	349	C2	CuC	X	1	20.780	7.090	24.260	0.00	0.00
ATOM	350	C2	CuC	X	1	20.780	7.090	40.560	0.00	0.00
ATOM	351	C2	CuC	X	1	20.780	7.090	56.860	0.00	0.00
ATOM	352	C2	CuC	X	1	20.780	7.090	73.170	0.00	0.00

ATOM	353	C2	CuC	X	1	20.780	17.980	24.260	0.00	0.00
ATOM	354	C2	CuC	X	1	20.780	17.980	40.560	0.00	0.00
ATOM	355	C2	CuC	X	1	20.780	17.980	56.860	0.00	0.00
ATOM	356	C2	CuC	X	1	20.780	17.980	73.170	0.00	0.00
ATOM	357	C2	CuC	X	1	20.780	28.880	24.260	0.00	0.00
ATOM	358	C2	CuC	X	1	20.780	28.880	40.560	0.00	0.00
ATOM	359	C2	CuC	X	1	20.780	28.880	56.860	0.00	0.00
ATOM	360	C2	CuC	X	1	20.780	28.880	73.170	0.00	0.00
ATOM	361	C2	CuC	X	1	31.670	7.090	24.260	0.00	0.00
ATOM	362	C2	CuC	X	1	31.670	7.090	40.560	0.00	0.00
ATOM	363	C2	CuC	X	1	31.670	7.090	56.860	0.00	0.00
ATOM	364	C2	CuC	X	1	31.670	7.090	73.170	0.00	0.00
ATOM	365	C2	CuC	X	1	31.670	17.980	24.260	0.00	0.00
ATOM	366	C2	CuC	X	1	31.670	17.980	40.560	0.00	0.00
ATOM	367	C2	CuC	X	1	31.670	17.980	56.860	0.00	0.00
ATOM	368	C2	CuC	X	1	31.670	17.980	73.170	0.00	0.00
ATOM	369	C2	CuC	X	1	31.670	28.880	24.260	0.00	0.00
ATOM	370	C2	CuC	X	1	31.670	28.880	40.560	0.00	0.00
ATOM	371	C2	CuC	X	1	31.670	28.880	56.860	0.00	0.00
ATOM	372	C2	CuC	X	1	31.670	28.880	73.170	0.00	0.00
ATOM	373	C2	CuC	X	1	8.480	7.080	24.260	0.00	0.00
ATOM	374	C2	CuC	X	1	8.480	7.080	40.560	0.00	0.00
ATOM	375	C2	CuC	X	1	8.480	7.080	56.860	0.00	0.00
ATOM	376	C2	CuC	X	1	8.480	7.080	73.170	0.00	0.00

ATOM 377 C2 CuC X 1 8.480 17.970 24.260 0.00 0.00

ATOM 378 C2 CuC X 1 8.480 17.970 40.560 0.00 0.00

ATOM 379 C2 CuC X 1 8.480 17.970 56.860 0.00 0.00

ATOM 380 C2 CuC X 1 8.480 17.970 73.170 0.00 0.00

ATOM 381 C2 CuC X 1 8.480 28.860 24.260 0.00 0.00

ATOM 382 C2 CuC X 1 8.480 28.860 40.560 0.00 0.00

ATOM 383 C2 CuC X 1 8.480 28.860 56.860 0.00 0.00

ATOM 384 C2 CuC X 1 8.480 28.860 73.170 0.00 0.00

ATOM 385 C2 CuC X 1 19.370 7.080 24.260 0.00 0.00

ATOM 386 C2 CuC X 1 19.370 7.080 40.560 0.00 0.00

ATOM 387 C2 CuC X 1 19.370 7.080 56.860 0.00 0.00

ATOM 388 C2 CuC X 1 19.370 7.080 73.170 0.00 0.00

ATOM 389 C2 CuC X 1 19.370 17.970 24.260 0.00 0.00

ATOM 390 C2 CuC X 1 19.370 17.970 40.560 0.00 0.00

ATOM 391 C2 CuC X 1 19.370 17.970 56.860 0.00 0.00

ATOM 392 C2 CuC X 1 19.370 17.970 73.170 0.00 0.00

ATOM 393 C2 CuC X 1 19.370 28.860 24.260 0.00 0.00

ATOM 394 C2 CuC X 1 19.370 28.860 40.560 0.00 0.00

ATOM 395 C2 CuC X 1 19.370 28.860 56.860 0.00 0.00

ATOM 396 C2 CuC X 1 19.370 28.860 73.170 0.00 0.00

ATOM 397 C2 CuC X 1 30.260 7.080 24.260 0.00 0.00

ATOM 398 C2 CuC X 1 30.260 7.080 40.560 0.00 0.00

ATOM 399 C2 CuC X 1 30.260 7.080 56.860 0.00 0.00

ATOM 400 C2 CuC X 1 30.260 7.080 73.170 0.00 0.00

```
ATOM    401  C2  CuC X   1      30.260  17.970  24.260  0.00  0.00
ATOM    402  C2  CuC X   1      30.260  17.970  40.560  0.00  0.00
ATOM    403  C2  CuC X   1      30.260  17.970  56.860  0.00  0.00
ATOM    404  C2  CuC X   1      30.260  17.970  73.170  0.00  0.00
ATOM    405  C2  CuC X   1      30.260  28.860  24.260  0.00  0.00
ATOM    406  C2  CuC X   1      30.260  28.860  40.560  0.00  0.00
ATOM    407  C2  CuC X   1      30.260  28.860  56.860  0.00  0.00
ATOM    408  C2  CuC X   1      30.260  28.860  73.170  0.00  0.00
ATOM    409  C2  CuC X   1       8.480   7.140  26.710  0.00  0.00
ATOM    410  C2  CuC X   1       8.480   7.140  43.020  0.00  0.00
ATOM    411  C2  CuC X   1       8.480   7.140  59.320  0.00  0.00
ATOM    412  C2  CuC X   1       8.480   7.140  75.620  0.00  0.00
ATOM    413  C2  CuC X   1       8.480  18.030  26.710  0.00  0.00
ATOM    414  C2  CuC X   1       8.480  18.030  43.020  0.00  0.00
ATOM    415  C2  CuC X   1       8.480  18.030  59.320  0.00  0.00
ATOM    416  C2  CuC X   1       8.480  18.030  75.620  0.00  0.00
ATOM    417  C2  CuC X   1       8.480  28.920  26.710  0.00  0.00
ATOM    418  C2  CuC X   1       8.480  28.920  43.020  0.00  0.00
ATOM    419  C2  CuC X   1       8.480  28.920  59.320  0.00  0.00
ATOM    420  C2  CuC X   1       8.480  28.920  75.620  0.00  0.00
ATOM    421  C2  CuC X   1      19.380   7.140  26.710  0.00  0.00
ATOM    422  C2  CuC X   1      19.380   7.140  43.020  0.00  0.00
ATOM    423  C2  CuC X   1      19.380   7.140  59.320  0.00  0.00
ATOM    424  C2  CuC X   1      19.380   7.140  75.620  0.00  0.00
```

ATOM	425	C2	CuC	X	1	19.380	18.030	26.710	0.00	0.00
ATOM	426	C2	CuC	X	1	19.380	18.030	43.020	0.00	0.00
ATOM	427	C2	CuC	X	1	19.380	18.030	59.320	0.00	0.00
ATOM	428	C2	CuC	X	1	19.380	18.030	75.620	0.00	0.00
ATOM	429	C2	CuC	X	1	19.380	28.920	26.710	0.00	0.00
ATOM	430	C2	CuC	X	1	19.380	28.920	43.020	0.00	0.00
ATOM	431	C2	CuC	X	1	19.380	28.920	59.320	0.00	0.00
ATOM	432	C2	CuC	X	1	19.380	28.920	75.620	0.00	0.00
ATOM	433	C2	CuC	X	1	30.270	7.140	26.710	0.00	0.00
ATOM	434	C2	CuC	X	1	30.270	7.140	43.020	0.00	0.00
ATOM	435	C2	CuC	X	1	30.270	7.140	59.320	0.00	0.00
ATOM	436	C2	CuC	X	1	30.270	7.140	75.620	0.00	0.00
ATOM	437	C2	CuC	X	1	30.270	18.030	26.710	0.00	0.00
ATOM	438	C2	CuC	X	1	30.270	18.030	43.020	0.00	0.00
ATOM	439	C2	CuC	X	1	30.270	18.030	59.320	0.00	0.00
ATOM	440	C2	CuC	X	1	30.270	18.030	75.620	0.00	0.00
ATOM	441	C2	CuC	X	1	30.270	28.920	26.710	0.00	0.00
ATOM	442	C2	CuC	X	1	30.270	28.920	43.020	0.00	0.00
ATOM	443	C2	CuC	X	1	30.270	28.920	59.320	0.00	0.00
ATOM	444	C2	CuC	X	1	30.270	28.920	75.620	0.00	0.00
ATOM	445	C2	CuC	X	1	9.890	7.120	26.710	0.00	0.00
ATOM	446	C2	CuC	X	1	9.890	7.120	43.020	0.00	0.00
ATOM	447	C2	CuC	X	1	9.890	7.120	59.320	0.00	0.00
ATOM	448	C2	CuC	X	1	9.890	7.120	75.620	0.00	0.00

ATOM 449 C2 CuC X 1 9.890 18.010 26.710 0.00 0.00

ATOM 450 C2 CuC X 1 9.890 18.010 43.020 0.00 0.00

ATOM 451 C2 CuC X 1 9.890 18.010 59.320 0.00 0.00

ATOM 452 C2 CuC X 1 9.890 18.010 75.620 0.00 0.00

ATOM 453 C2 CuC X 1 9.890 28.900 26.710 0.00 0.00

ATOM 454 C2 CuC X 1 9.890 28.900 43.020 0.00 0.00

ATOM 455 C2 CuC X 1 9.890 28.900 59.320 0.00 0.00

ATOM 456 C2 CuC X 1 9.890 28.900 75.620 0.00 0.00

ATOM 457 C2 CuC X 1 20.780 7.120 26.710 0.00 0.00

ATOM 458 C2 CuC X 1 20.780 7.120 43.020 0.00 0.00

ATOM 459 C2 CuC X 1 20.780 7.120 59.320 0.00 0.00

ATOM 460 C2 CuC X 1 20.780 7.120 75.620 0.00 0.00

ATOM 461 C2 CuC X 1 20.780 18.010 26.710 0.00 0.00

ATOM 462 C2 CuC X 1 20.780 18.010 43.020 0.00 0.00

ATOM 463 C2 CuC X 1 20.780 18.010 59.320 0.00 0.00

ATOM 464 C2 CuC X 1 20.780 18.010 75.620 0.00 0.00

ATOM 465 C2 CuC X 1 20.780 28.900 26.710 0.00 0.00

ATOM 466 C2 CuC X 1 20.780 28.900 43.020 0.00 0.00

ATOM 467 C2 CuC X 1 20.780 28.900 59.320 0.00 0.00

ATOM 468 C2 CuC X 1 20.780 28.900 75.620 0.00 0.00

ATOM 469 C2 CuC X 1 31.670 7.120 26.710 0.00 0.00

ATOM 470 C2 CuC X 1 31.670 7.120 43.020 0.00 0.00

ATOM 471 C2 CuC X 1 31.670 7.120 59.320 0.00 0.00

ATOM 472 C2 CuC X 1 31.670 7.120 75.620 0.00 0.00

ATOM	473	C2	CuC	X	1	31.670	18.010	26.710	0.00	0.00
ATOM	474	C2	CuC	X	1	31.670	18.010	43.020	0.00	0.00
ATOM	475	C2	CuC	X	1	31.670	18.010	59.320	0.00	0.00
ATOM	476	C2	CuC	X	1	31.670	18.010	75.620	0.00	0.00
ATOM	477	C2	CuC	X	1	31.670	28.900	26.710	0.00	0.00
ATOM	478	C2	CuC	X	1	31.670	28.900	43.020	0.00	0.00
ATOM	479	C2	CuC	X	1	31.670	28.900	59.320	0.00	0.00
ATOM	480	C2	CuC	X	1	31.670	28.900	75.620	0.00	0.00
ATOM	481	N	CuC	X	1	3.730	7.280	28.960	0.00	0.00
ATOM	482	N	CuC	X	1	3.730	7.280	45.270	0.00	0.00
ATOM	483	N	CuC	X	1	3.730	7.280	61.570	0.00	0.00
ATOM	484	N	CuC	X	1	3.730	18.170	28.960	0.00	0.00
ATOM	485	N	CuC	X	1	3.730	18.170	45.270	0.00	0.00
ATOM	486	N	CuC	X	1	3.730	18.170	61.570	0.00	0.00
ATOM	487	N	CuC	X	1	3.730	29.070	28.960	0.00	0.00
ATOM	488	N	CuC	X	1	3.730	29.070	45.270	0.00	0.00
ATOM	489	N	CuC	X	1	3.730	29.070	61.570	0.00	0.00
ATOM	490	N	CuC	X	1	14.620	7.280	28.960	0.00	0.00
ATOM	491	N	CuC	X	1	14.620	7.280	45.270	0.00	0.00
ATOM	492	N	CuC	X	1	14.620	7.280	61.570	0.00	0.00
ATOM	493	N	CuC	X	1	14.620	18.170	28.960	0.00	0.00
ATOM	494	N	CuC	X	1	14.620	18.170	45.270	0.00	0.00
ATOM	495	N	CuC	X	1	14.620	18.170	61.570	0.00	0.00
ATOM	496	N	CuC	X	1	14.620	29.070	28.960	0.00	0.00

ATOM	497	N	CuC	X	1	14.620	29.070	45.270	0.00	0.00
ATOM	498	N	CuC	X	1	14.620	29.070	61.570	0.00	0.00
ATOM	499	N	CuC	X	1	25.510	7.280	28.960	0.00	0.00
ATOM	500	N	CuC	X	1	25.510	7.280	45.270	0.00	0.00
ATOM	501	N	CuC	X	1	25.510	7.280	61.570	0.00	0.00
ATOM	502	N	CuC	X	1	25.510	18.170	28.960	0.00	0.00
ATOM	503	N	CuC	X	1	25.510	18.170	45.270	0.00	0.00
ATOM	504	N	CuC	X	1	25.510	18.170	61.570	0.00	0.00
ATOM	505	N	CuC	X	1	25.510	29.070	28.960	0.00	0.00
ATOM	506	N	CuC	X	1	25.510	29.070	45.270	0.00	0.00
ATOM	507	N	CuC	X	1	25.510	29.070	61.570	0.00	0.00
ATOM	508	C3	CuC	X	1	3.970	6.190	29.730	0.00	0.00
ATOM	509	C3	CuC	X	1	3.970	6.190	46.030	0.00	0.00
ATOM	510	C3	CuC	X	1	3.970	6.190	62.340	0.00	0.00
ATOM	511	C3	CuC	X	1	3.970	17.080	29.730	0.00	0.00
ATOM	512	C3	CuC	X	1	3.970	17.080	46.030	0.00	0.00
ATOM	513	C3	CuC	X	1	3.970	17.080	62.340	0.00	0.00
ATOM	514	C3	CuC	X	1	3.970	27.970	29.730	0.00	0.00
ATOM	515	C3	CuC	X	1	3.970	27.970	46.030	0.00	0.00
ATOM	516	C3	CuC	X	1	3.970	27.970	62.340	0.00	0.00
ATOM	517	C3	CuC	X	1	14.870	6.190	29.730	0.00	0.00
ATOM	518	C3	CuC	X	1	14.870	6.190	46.030	0.00	0.00
ATOM	519	C3	CuC	X	1	14.870	6.190	62.340	0.00	0.00
ATOM	520	C3	CuC	X	1	14.870	17.080	29.730	0.00	0.00

```
ATOM    521  C3  CuC X   1      14.870  17.080  46.030  0.00  0.00
ATOM    522  C3  CuC X   1      14.870  17.080  62.340  0.00  0.00
ATOM    523  C3  CuC X   1      14.870  27.970  29.730  0.00  0.00
ATOM    524  C3  CuC X   1      14.870  27.970  46.030  0.00  0.00
ATOM    525  C3  CuC X   1      14.870  27.970  62.340  0.00  0.00
ATOM    526  C3  CuC X   1      25.760   6.190  29.730  0.00  0.00
ATOM    527  C3  CuC X   1      25.760   6.190  46.030  0.00  0.00
ATOM    528  C3  CuC X   1      25.760   6.190  62.340  0.00  0.00
ATOM    529  C3  CuC X   1      25.760  17.080  29.730  0.00  0.00
ATOM    530  C3  CuC X   1      25.760  17.080  46.030  0.00  0.00
ATOM    531  C3  CuC X   1      25.760  17.080  62.340  0.00  0.00
ATOM    532  C3  CuC X   1      25.760  27.970  29.730  0.00  0.00
ATOM    533  C3  CuC X   1      25.760  27.970  46.030  0.00  0.00
ATOM    534  C3  CuC X   1      25.760  27.970  62.340  0.00  0.00
ATOM    535  C4  CuC X   1       4.000   6.230  31.130  0.00  0.00
ATOM    536  C4  CuC X   1       4.000   6.230  47.430  0.00  0.00
ATOM    537  C4  CuC X   1       4.000   6.230  63.740  0.00  0.00
ATOM    538  C4  CuC X   1       4.000  17.130  31.130  0.00  0.00
ATOM    539  C4  CuC X   1       4.000  17.130  47.430  0.00  0.00
ATOM    540  C4  CuC X   1       4.000  17.130  63.740  0.00  0.00
ATOM    541  C4  CuC X   1       4.000  28.020  31.130  0.00  0.00
ATOM    542  C4  CuC X   1       4.000  28.020  47.430  0.00  0.00
ATOM    543  C4  CuC X   1       4.000  28.020  63.740  0.00  0.00
ATOM    544  C4  CuC X   1      14.890   6.230  31.130  0.00  0.00
```

ATOM	545	C4	CuC	X	1	14.890	6.230	47.430	0.00	0.00
ATOM	546	C4	CuC	X	1	14.890	6.230	63.740	0.00	0.00
ATOM	547	C4	CuC	X	1	14.890	17.130	31.130	0.00	0.00
ATOM	548	C4	CuC	X	1	14.890	17.130	47.430	0.00	0.00
ATOM	549	C4	CuC	X	1	14.890	17.130	63.740	0.00	0.00
ATOM	550	C4	CuC	X	1	14.890	28.020	31.130	0.00	0.00
ATOM	551	C4	CuC	X	1	14.890	28.020	47.430	0.00	0.00
ATOM	552	C4	CuC	X	1	14.890	28.020	63.740	0.00	0.00
ATOM	553	C4	CuC	X	1	25.780	6.230	31.130	0.00	0.00
ATOM	554	C4	CuC	X	1	25.780	6.230	47.430	0.00	0.00
ATOM	555	C4	CuC	X	1	25.780	6.230	63.740	0.00	0.00
ATOM	556	C4	CuC	X	1	25.780	17.130	31.130	0.00	0.00
ATOM	557	C4	CuC	X	1	25.780	17.130	47.430	0.00	0.00
ATOM	558	C4	CuC	X	1	25.780	17.130	63.740	0.00	0.00
ATOM	559	C4	CuC	X	1	25.780	28.020	31.130	0.00	0.00
ATOM	560	C4	CuC	X	1	25.780	28.020	47.430	0.00	0.00
ATOM	561	C4	CuC	X	1	25.780	28.020	63.740	0.00	0.00
ATOM	562	C5	CuC	X	1	3.750	7.470	31.790	0.00	0.00
ATOM	563	C5	CuC	X	1	3.750	7.470	48.100	0.00	0.00
ATOM	564	C5	CuC	X	1	3.750	7.470	64.400	0.00	0.00
ATOM	565	C5	CuC	X	1	3.750	18.360	31.790	0.00	0.00
ATOM	566	C5	CuC	X	1	3.750	18.360	48.100	0.00	0.00
ATOM	567	C5	CuC	X	1	3.750	18.360	64.400	0.00	0.00
ATOM	568	C5	CuC	X	1	3.750	29.250	31.790	0.00	0.00

ATOM	569	C5	CuC	X	1	3.750	29.250	48.100	0.00	0.00
ATOM	570	C5	CuC	X	1	3.750	29.250	64.400	0.00	0.00
ATOM	571	C5	CuC	X	1	14.640	7.470	31.790	0.00	0.00
ATOM	572	C5	CuC	X	1	14.640	7.470	48.100	0.00	0.00
ATOM	573	C5	CuC	X	1	14.640	7.470	64.400	0.00	0.00
ATOM	574	C5	CuC	X	1	14.640	18.360	31.790	0.00	0.00
ATOM	575	C5	CuC	X	1	14.640	18.360	48.100	0.00	0.00
ATOM	576	C5	CuC	X	1	14.640	18.360	64.400	0.00	0.00
ATOM	577	C5	CuC	X	1	14.640	29.250	31.790	0.00	0.00
ATOM	578	C5	CuC	X	1	14.640	29.250	48.100	0.00	0.00
ATOM	579	C5	CuC	X	1	14.640	29.250	64.400	0.00	0.00
ATOM	580	C5	CuC	X	1	25.530	7.470	31.790	0.00	0.00
ATOM	581	C5	CuC	X	1	25.530	7.470	48.100	0.00	0.00
ATOM	582	C5	CuC	X	1	25.530	7.470	64.400	0.00	0.00
ATOM	583	C5	CuC	X	1	25.530	18.360	31.790	0.00	0.00
ATOM	584	C5	CuC	X	1	25.530	18.360	48.100	0.00	0.00
ATOM	585	C5	CuC	X	1	25.530	18.360	64.400	0.00	0.00
ATOM	586	C5	CuC	X	1	25.530	29.250	31.790	0.00	0.00
ATOM	587	C5	CuC	X	1	25.530	29.250	48.100	0.00	0.00
ATOM	588	C5	CuC	X	1	25.530	29.250	64.400	0.00	0.00
ATOM	589	C4	CuC	X	1	3.500	8.600	30.980	0.00	0.00
ATOM	590	C4	CuC	X	1	3.500	8.600	47.280	0.00	0.00
ATOM	591	C4	CuC	X	1	3.500	8.600	63.580	0.00	0.00
ATOM	592	C4	CuC	X	1	3.500	19.490	30.980	0.00	0.00

ATOM 593 C4 CuC X 1 3.500 19.490 47.280 0.00 0.00

ATOM 594 C4 CuC X 1 3.500 19.490 63.580 0.00 0.00

ATOM 595 C4 CuC X 1 3.500 30.380 30.980 0.00 0.00

ATOM 596 C4 CuC X 1 3.500 30.380 47.280 0.00 0.00

ATOM 597 C4 CuC X 1 3.500 30.380 63.580 0.00 0.00

ATOM 598 C4 CuC X 1 14.390 8.600 30.980 0.00 0.00

ATOM 599 C4 CuC X 1 14.390 8.600 47.280 0.00 0.00

ATOM 600 C4 CuC X 1 14.390 8.600 63.580 0.00 0.00

ATOM 601 C4 CuC X 1 14.390 19.490 30.980 0.00 0.00

ATOM 602 C4 CuC X 1 14.390 19.490 47.280 0.00 0.00

ATOM 603 C4 CuC X 1 14.390 19.490 63.580 0.00 0.00

ATOM 604 C4 CuC X 1 14.390 30.380 30.980 0.00 0.00

ATOM 605 C4 CuC X 1 14.390 30.380 47.280 0.00 0.00

ATOM 606 C4 CuC X 1 14.390 30.380 63.580 0.00 0.00

ATOM 607 C4 CuC X 1 25.280 8.600 30.980 0.00 0.00

ATOM 608 C4 CuC X 1 25.280 8.600 47.280 0.00 0.00

ATOM 609 C4 CuC X 1 25.280 8.600 63.580 0.00 0.00

ATOM 610 C4 CuC X 1 25.280 19.490 30.980 0.00 0.00

ATOM 611 C4 CuC X 1 25.280 19.490 47.280 0.00 0.00

ATOM 612 C4 CuC X 1 25.280 19.490 63.580 0.00 0.00

ATOM 613 C4 CuC X 1 25.280 30.380 30.980 0.00 0.00

ATOM 614 C4 CuC X 1 25.280 30.380 47.280 0.00 0.00

ATOM 615 C4 CuC X 1 25.280 30.380 63.580 0.00 0.00

ATOM 616 C3 CuC X 1 3.500 8.470 29.580 0.00 0.00

ATOM 617 C3 CuC X 1 3.500 8.470 45.880 0.00 0.00

ATOM 618 C3 CuC X 1 3.500 8.470 62.180 0.00 0.00

ATOM 619 C3 CuC X 1 3.500 19.360 29.580 0.00 0.00

ATOM 620 C3 CuC X 1 3.500 19.360 45.880 0.00 0.00

ATOM 621 C3 CuC X 1 3.500 19.360 62.180 0.00 0.00

ATOM 622 C3 CuC X 1 3.500 30.250 29.580 0.00 0.00

ATOM 623 C3 CuC X 1 3.500 30.250 45.880 0.00 0.00

ATOM 624 C3 CuC X 1 3.500 30.250 62.180 0.00 0.00

ATOM 625 C3 CuC X 1 14.390 8.470 29.580 0.00 0.00

ATOM 626 C3 CuC X 1 14.390 8.470 45.880 0.00 0.00

ATOM 627 C3 CuC X 1 14.390 8.470 62.180 0.00 0.00

ATOM 628 C3 CuC X 1 14.390 19.360 29.580 0.00 0.00

ATOM 629 C3 CuC X 1 14.390 19.360 45.880 0.00 0.00

ATOM 630 C3 CuC X 1 14.390 19.360 62.180 0.00 0.00

ATOM 631 C3 CuC X 1 14.390 30.250 29.580 0.00 0.00

ATOM 632 C3 CuC X 1 14.390 30.250 45.880 0.00 0.00

ATOM 633 C3 CuC X 1 14.390 30.250 62.180 0.00 0.00

ATOM 634 C3 CuC X 1 25.280 8.470 29.580 0.00 0.00

ATOM 635 C3 CuC X 1 25.280 8.470 45.880 0.00 0.00

ATOM 636 C3 CuC X 1 25.280 8.470 62.180 0.00 0.00

ATOM 637 C3 CuC X 1 25.280 19.360 29.580 0.00 0.00

ATOM 638 C3 CuC X 1 25.280 19.360 45.880 0.00 0.00

ATOM 639 C3 CuC X 1 25.280 19.360 62.180 0.00 0.00

ATOM 640 C3 CuC X 1 25.280 30.250 29.580 0.00 0.00

ATOM	641	C3	CuC	X	1	25.280	30.250	45.880	0.00	0.00
ATOM	642	C3	CuC	X	1	25.280	30.250	62.180	0.00	0.00
ATOM	643	C6	CuC	X	1	3.720	7.620	33.250	0.00	0.00
ATOM	644	C6	CuC	X	1	3.720	7.620	49.550	0.00	0.00
ATOM	645	C6	CuC	X	1	3.720	7.620	65.860	0.00	0.00
ATOM	646	C6	CuC	X	1	3.720	18.510	33.250	0.00	0.00
ATOM	647	C6	CuC	X	1	3.720	18.510	49.550	0.00	0.00
ATOM	648	C6	CuC	X	1	3.720	18.510	65.860	0.00	0.00
ATOM	649	C6	CuC	X	1	3.720	29.410	33.250	0.00	0.00
ATOM	650	C6	CuC	X	1	3.720	29.410	49.550	0.00	0.00
ATOM	651	C6	CuC	X	1	3.720	29.410	65.860	0.00	0.00
ATOM	652	C6	CuC	X	1	14.610	7.620	33.250	0.00	0.00
ATOM	653	C6	CuC	X	1	14.610	7.620	49.550	0.00	0.00
ATOM	654	C6	CuC	X	1	14.610	7.620	65.860	0.00	0.00
ATOM	655	C6	CuC	X	1	14.610	18.510	33.250	0.00	0.00
ATOM	656	C6	CuC	X	1	14.610	18.510	49.550	0.00	0.00
ATOM	657	C6	CuC	X	1	14.610	18.510	65.860	0.00	0.00
ATOM	658	C6	CuC	X	1	14.610	29.410	33.250	0.00	0.00
ATOM	659	C6	CuC	X	1	14.610	29.410	49.550	0.00	0.00
ATOM	660	C6	CuC	X	1	14.610	29.410	65.860	0.00	0.00
ATOM	661	C6	CuC	X	1	25.500	7.620	33.250	0.00	0.00
ATOM	662	C6	CuC	X	1	25.500	7.620	49.550	0.00	0.00
ATOM	663	C6	CuC	X	1	25.500	7.620	65.860	0.00	0.00
ATOM	664	C6	CuC	X	1	25.500	18.510	33.250	0.00	0.00

ATOM 665 C6 CuC X 1 25.500 18.510 49.550 0.00 0.00

ATOM 666 C6 CuC X 1 25.500 18.510 65.860 0.00 0.00

ATOM 667 C6 CuC X 1 25.500 29.410 33.250 0.00 0.00

ATOM 668 C6 CuC X 1 25.500 29.410 49.550 0.00 0.00

ATOM 669 C6 CuC X 1 25.500 29.410 65.860 0.00 0.00

ATOM 670 C6 CuC X 1 4.000 6.640 34.160 0.00 0.00

ATOM 671 C6 CuC X 1 4.000 6.640 50.460 0.00 0.00

ATOM 672 C6 CuC X 1 4.000 6.640 66.770 0.00 0.00

ATOM 673 C6 CuC X 1 4.000 17.540 34.160 0.00 0.00

ATOM 674 C6 CuC X 1 4.000 17.540 50.460 0.00 0.00

ATOM 675 C6 CuC X 1 4.000 17.540 66.770 0.00 0.00

ATOM 676 C6 CuC X 1 4.000 28.430 34.160 0.00 0.00

ATOM 677 C6 CuC X 1 4.000 28.430 50.460 0.00 0.00

ATOM 678 C6 CuC X 1 4.000 28.430 66.770 0.00 0.00

ATOM 679 C6 CuC X 1 14.890 6.640 34.160 0.00 0.00

ATOM 680 C6 CuC X 1 14.890 6.640 50.460 0.00 0.00

ATOM 681 C6 CuC X 1 14.890 6.640 66.770 0.00 0.00

ATOM 682 C6 CuC X 1 14.890 17.540 34.160 0.00 0.00

ATOM 683 C6 CuC X 1 14.890 17.540 50.460 0.00 0.00

ATOM 684 C6 CuC X 1 14.890 17.540 66.770 0.00 0.00

ATOM 685 C6 CuC X 1 14.890 28.430 34.160 0.00 0.00

ATOM 686 C6 CuC X 1 14.890 28.430 50.460 0.00 0.00

ATOM 687 C6 CuC X 1 14.890 28.430 66.770 0.00 0.00

ATOM 688 C6 CuC X 1 25.780 6.640 34.160 0.00 0.00

ATOM	689	C6	CuC	X	1	25.780	6.640	50.460	0.00	0.00
ATOM	690	C6	CuC	X	1	25.780	6.640	66.770	0.00	0.00
ATOM	691	C6	CuC	X	1	25.780	17.540	34.160	0.00	0.00
ATOM	692	C6	CuC	X	1	25.780	17.540	50.460	0.00	0.00
ATOM	693	C6	CuC	X	1	25.780	17.540	66.770	0.00	0.00
ATOM	694	C6	CuC	X	1	25.780	28.430	34.160	0.00	0.00
ATOM	695	C6	CuC	X	1	25.780	28.430	50.460	0.00	0.00
ATOM	696	C6	CuC	X	1	25.780	28.430	66.770	0.00	0.00
ATOM	697	C5	CuC	X	1	3.920	6.800	35.620	0.00	0.00
ATOM	698	C5	CuC	X	1	3.920	6.800	51.920	0.00	0.00
ATOM	699	C5	CuC	X	1	3.920	6.800	68.220	0.00	0.00
ATOM	700	C5	CuC	X	1	3.920	17.690	35.620	0.00	0.00
ATOM	701	C5	CuC	X	1	3.920	17.690	51.920	0.00	0.00
ATOM	702	C5	CuC	X	1	3.920	17.690	68.220	0.00	0.00
ATOM	703	C5	CuC	X	1	3.920	28.580	35.620	0.00	0.00
ATOM	704	C5	CuC	X	1	3.920	28.580	51.920	0.00	0.00
ATOM	705	C5	CuC	X	1	3.920	28.580	68.220	0.00	0.00
ATOM	706	C5	CuC	X	1	14.810	6.800	35.620	0.00	0.00
ATOM	707	C5	CuC	X	1	14.810	6.800	51.920	0.00	0.00
ATOM	708	C5	CuC	X	1	14.810	6.800	68.220	0.00	0.00
ATOM	709	C5	CuC	X	1	14.810	17.690	35.620	0.00	0.00
ATOM	710	C5	CuC	X	1	14.810	17.690	51.920	0.00	0.00
ATOM	711	C5	CuC	X	1	14.810	17.690	68.220	0.00	0.00
ATOM	712	C5	CuC	X	1	14.810	28.580	35.620	0.00	0.00

ATOM 713 C5 CuC X 1 14.810 28.580 51.920 0.00 0.00

ATOM 714 C5 CuC X 1 14.810 28.580 68.220 0.00 0.00

ATOM 715 C5 CuC X 1 25.700 6.800 35.620 0.00 0.00

ATOM 716 C5 CuC X 1 25.700 6.800 51.920 0.00 0.00

ATOM 717 C5 CuC X 1 25.700 6.800 68.220 0.00 0.00

ATOM 718 C5 CuC X 1 25.700 17.690 35.620 0.00 0.00

ATOM 719 C5 CuC X 1 25.700 17.690 51.920 0.00 0.00

ATOM 720 C5 CuC X 1 25.700 17.690 68.220 0.00 0.00

ATOM 721 C5 CuC X 1 25.700 28.580 35.620 0.00 0.00

ATOM 722 C5 CuC X 1 25.700 28.580 51.920 0.00 0.00

ATOM 723 C5 CuC X 1 25.700 28.580 68.220 0.00 0.00

ATOM 724 C4 CuC X 1 3.410 7.960 36.260 0.00 0.00

ATOM 725 C4 CuC X 1 3.410 7.960 52.560 0.00 0.00

ATOM 726 C4 CuC X 1 3.410 7.960 68.870 0.00 0.00

ATOM 727 C4 CuC X 1 3.410 18.850 36.260 0.00 0.00

ATOM 728 C4 CuC X 1 3.410 18.850 52.560 0.00 0.00

ATOM 729 C4 CuC X 1 3.410 18.850 68.870 0.00 0.00

ATOM 730 C4 CuC X 1 3.410 29.740 36.260 0.00 0.00

ATOM 731 C4 CuC X 1 3.410 29.740 52.560 0.00 0.00

ATOM 732 C4 CuC X 1 3.410 29.740 68.870 0.00 0.00

ATOM 733 C4 CuC X 1 14.300 7.960 36.260 0.00 0.00

ATOM 734 C4 CuC X 1 14.300 7.960 52.560 0.00 0.00

ATOM 735 C4 CuC X 1 14.300 7.960 68.870 0.00 0.00

ATOM 736 C4 CuC X 1 14.300 18.850 36.260 0.00 0.00

ATOM 737 C4 CuC X 1 14.300 18.850 52.560 0.00 0.00

ATOM 738 C4 CuC X 1 14.300 18.850 68.870 0.00 0.00

ATOM 739 C4 CuC X 1 14.300 29.740 36.260 0.00 0.00

ATOM 740 C4 CuC X 1 14.300 29.740 52.560 0.00 0.00

ATOM 741 C4 CuC X 1 14.300 29.740 68.870 0.00 0.00

ATOM 742 C4 CuC X 1 25.200 7.960 36.260 0.00 0.00

ATOM 743 C4 CuC X 1 25.200 7.960 52.560 0.00 0.00

ATOM 744 C4 CuC X 1 25.200 7.960 68.870 0.00 0.00

ATOM 745 C4 CuC X 1 25.200 18.850 36.260 0.00 0.00

ATOM 746 C4 CuC X 1 25.200 18.850 52.560 0.00 0.00

ATOM 747 C4 CuC X 1 25.200 18.850 68.870 0.00 0.00

ATOM 748 C4 CuC X 1 25.200 29.740 36.260 0.00 0.00

ATOM 749 C4 CuC X 1 25.200 29.740 52.560 0.00 0.00

ATOM 750 C4 CuC X 1 25.200 29.740 68.870 0.00 0.00

ATOM 751 C3 CuC X 1 3.370 8.000 37.660 0.00 0.00

ATOM 752 C3 CuC X 1 3.370 8.000 53.960 0.00 0.00

ATOM 753 C3 CuC X 1 3.370 8.000 70.270 0.00 0.00

ATOM 754 C3 CuC X 1 3.370 18.890 37.660 0.00 0.00

ATOM 755 C3 CuC X 1 3.370 18.890 53.960 0.00 0.00

ATOM 756 C3 CuC X 1 3.370 18.890 70.270 0.00 0.00

ATOM 757 C3 CuC X 1 3.370 29.780 37.660 0.00 0.00

ATOM 758 C3 CuC X 1 3.370 29.780 53.960 0.00 0.00

ATOM 759 C3 CuC X 1 3.370 29.780 70.270 0.00 0.00

ATOM 760 C3 CuC X 1 14.260 8.000 37.660 0.00 0.00

ATOM 761 C3 CuC X 1 14.260 8.000 53.960 0.00 0.00

ATOM 762 C3 CuC X 1 14.260 8.000 70.270 0.00 0.00

ATOM 763 C3 CuC X 1 14.260 18.890 37.660 0.00 0.00

ATOM 764 C3 CuC X 1 14.260 18.890 53.960 0.00 0.00

ATOM 765 C3 CuC X 1 14.260 18.890 70.270 0.00 0.00

ATOM 766 C3 CuC X 1 14.260 29.780 37.660 0.00 0.00

ATOM 767 C3 CuC X 1 14.260 29.780 53.960 0.00 0.00

ATOM 768 C3 CuC X 1 14.260 29.780 70.270 0.00 0.00

ATOM 769 C3 CuC X 1 25.150 8.000 37.660 0.00 0.00

ATOM 770 C3 CuC X 1 25.150 8.000 53.960 0.00 0.00

ATOM 771 C3 CuC X 1 25.150 8.000 70.270 0.00 0.00

ATOM 772 C3 CuC X 1 25.150 18.890 37.660 0.00 0.00

ATOM 773 C3 CuC X 1 25.150 18.890 53.960 0.00 0.00

ATOM 774 C3 CuC X 1 25.150 18.890 70.270 0.00 0.00

ATOM 775 C3 CuC X 1 25.150 29.780 37.660 0.00 0.00

ATOM 776 C3 CuC X 1 25.150 29.780 53.960 0.00 0.00

ATOM 777 C3 CuC X 1 25.150 29.780 70.270 0.00 0.00

ATOM 778 N CuC X 1 3.770 6.970 38.440 0.00 0.00

ATOM 779 N CuC X 1 3.770 6.970 54.740 0.00 0.00

ATOM 780 N CuC X 1 3.770 6.970 71.050 0.00 0.00

ATOM 781 N CuC X 1 3.770 17.860 38.440 0.00 0.00

ATOM 782 N CuC X 1 3.770 17.860 54.740 0.00 0.00

ATOM 783 N CuC X 1 3.770 17.860 71.050 0.00 0.00

ATOM 784 N CuC X 1 3.770 28.750 38.440 0.00 0.00

ATOM 785 N CuC X 1 3.770 28.750 54.740 0.00 0.00

ATOM 786 N CuC X 1 3.770 28.750 71.050 0.00 0.00

ATOM 787 N CuC X 1 14.670 6.970 38.440 0.00 0.00

ATOM 788 N CuC X 1 14.670 6.970 54.740 0.00 0.00

ATOM 789 N CuC X 1 14.670 6.970 71.050 0.00 0.00

ATOM 790 N CuC X 1 14.670 17.860 38.440 0.00 0.00

ATOM 791 N CuC X 1 14.670 17.860 54.740 0.00 0.00

ATOM 792 N CuC X 1 14.670 17.860 71.050 0.00 0.00

ATOM 793 N CuC X 1 14.670 28.750 38.440 0.00 0.00

ATOM 794 N CuC X 1 14.670 28.750 54.740 0.00 0.00

ATOM 795 N CuC X 1 14.670 28.750 71.050 0.00 0.00

ATOM 796 N CuC X 1 25.560 6.970 38.440 0.00 0.00

ATOM 797 N CuC X 1 25.560 6.970 54.740 0.00 0.00

ATOM 798 N CuC X 1 25.560 6.970 71.050 0.00 0.00

ATOM 799 N CuC X 1 25.560 17.860 38.440 0.00 0.00

ATOM 800 N CuC X 1 25.560 17.860 54.740 0.00 0.00

ATOM 801 N CuC X 1 25.560 17.860 71.050 0.00 0.00

ATOM 802 N CuC X 1 25.560 28.750 38.440 0.00 0.00

ATOM 803 N CuC X 1 25.560 28.750 54.740 0.00 0.00

ATOM 804 N CuC X 1 25.560 28.750 71.050 0.00 0.00

ATOM 805 C3 CuC X 1 4.250 5.860 37.850 0.00 0.00

ATOM 806 C3 CuC X 1 4.250 5.860 54.150 0.00 0.00

ATOM 807 C3 CuC X 1 4.250 5.860 70.450 0.00 0.00

ATOM 808 C3 CuC X 1 4.250 16.750 37.850 0.00 0.00

ATOM 809 C3 CuC X 1 4.250 16.750 54.150 0.00 0.00

ATOM 810 C3 CuC X 1 4.250 16.750 70.450 0.00 0.00

ATOM 811 C3 CuC X 1 4.250 27.640 37.850 0.00 0.00

ATOM 812 C3 CuC X 1 4.250 27.640 54.150 0.00 0.00

ATOM 813 C3 CuC X 1 4.250 27.640 70.450 0.00 0.00

ATOM 814 C3 CuC X 1 15.140 5.860 37.850 0.00 0.00

ATOM 815 C3 CuC X 1 15.140 5.860 54.150 0.00 0.00

ATOM 816 C3 CuC X 1 15.140 5.860 70.450 0.00 0.00

ATOM 817 C3 CuC X 1 15.140 16.750 37.850 0.00 0.00

ATOM 818 C3 CuC X 1 15.140 16.750 54.150 0.00 0.00

ATOM 819 C3 CuC X 1 15.140 16.750 70.450 0.00 0.00

ATOM 820 C3 CuC X 1 15.140 27.640 37.850 0.00 0.00

ATOM 821 C3 CuC X 1 15.140 27.640 54.150 0.00 0.00

ATOM 822 C3 CuC X 1 15.140 27.640 70.450 0.00 0.00

ATOM 823 C3 CuC X 1 26.040 5.860 37.850 0.00 0.00

ATOM 824 C3 CuC X 1 26.040 5.860 54.150 0.00 0.00

ATOM 825 C3 CuC X 1 26.040 5.860 70.450 0.00 0.00

ATOM 826 C3 CuC X 1 26.040 16.750 37.850 0.00 0.00

ATOM 827 C3 CuC X 1 26.040 16.750 54.150 0.00 0.00

ATOM 828 C3 CuC X 1 26.040 16.750 70.450 0.00 0.00

ATOM 829 C3 CuC X 1 26.040 27.640 37.850 0.00 0.00

ATOM 830 C3 CuC X 1 26.040 27.640 54.150 0.00 0.00

ATOM 831 C3 CuC X 1 26.040 27.640 70.450 0.00 0.00

ATOM 832 C4 CuC X 1 4.340 5.730 36.450 0.00 0.00

```
ATOM    833  C4  CuC X   1       4.340   5.730  52.750  0.00  0.00
ATOM    834  C4  CuC X   1       4.340   5.730  69.060  0.00  0.00
ATOM    835  C4  CuC X   1       4.340  16.620  36.450  0.00  0.00
ATOM    836  C4  CuC X   1       4.340  16.620  52.750  0.00  0.00
ATOM    837  C4  CuC X   1       4.340  16.620  69.060  0.00  0.00
ATOM    838  C4  CuC X   1       4.340  27.510  36.450  0.00  0.00
ATOM    839  C4  CuC X   1       4.340  27.510  52.750  0.00  0.00
ATOM    840  C4  CuC X   1       4.340  27.510  69.060  0.00  0.00
ATOM    841  C4  CuC X   1      15.230   5.730  36.450  0.00  0.00
ATOM    842  C4  CuC X   1      15.230   5.730  52.750  0.00  0.00
ATOM    843  C4  CuC X   1      15.230   5.730  69.060  0.00  0.00
ATOM    844  C4  CuC X   1      15.230  16.620  36.450  0.00  0.00
ATOM    845  C4  CuC X   1      15.230  16.620  52.750  0.00  0.00
ATOM    846  C4  CuC X   1      15.230  16.620  69.060  0.00  0.00
ATOM    847  C4  CuC X   1      15.230  27.510  36.450  0.00  0.00
ATOM    848  C4  CuC X   1      15.230  27.510  52.750  0.00  0.00
ATOM    849  C4  CuC X   1      15.230  27.510  69.060  0.00  0.00
ATOM    850  C4  CuC X   1      26.130   5.730  36.450  0.00  0.00
ATOM    851  C4  CuC X   1      26.130   5.730  52.750  0.00  0.00
ATOM    852  C4  CuC X   1      26.130   5.730  69.060  0.00  0.00
ATOM    853  C4  CuC X   1      26.130  16.620  36.450  0.00  0.00
ATOM    854  C4  CuC X   1      26.130  16.620  52.750  0.00  0.00
ATOM    855  C4  CuC X   1      26.130  16.620  69.060  0.00  0.00
ATOM    856  C4  CuC X   1      26.130  27.510  36.450  0.00  0.00
```

ATOM 857 C4 CuC X 1 26.130 27.510 52.750 0.00 0.00

ATOM 858 C4 CuC X 1 26.130 27.510 69.060 0.00 0.00

ATOM 859 H2 CuC X 1 4.150 5.260 29.170 0.00 0.00

ATOM 860 H2 CuC X 1 4.150 5.260 45.470 0.00 0.00

ATOM 861 H2 CuC X 1 4.150 5.260 61.770 0.00 0.00

ATOM 862 H2 CuC X 1 4.150 16.150 29.170 0.00 0.00

ATOM 863 H2 CuC X 1 4.150 16.150 45.470 0.00 0.00

ATOM 864 H2 CuC X 1 4.150 16.150 61.770 0.00 0.00

ATOM 865 H2 CuC X 1 4.150 27.040 29.170 0.00 0.00

ATOM 866 H2 CuC X 1 4.150 27.040 45.470 0.00 0.00

ATOM 867 H2 CuC X 1 4.150 27.040 61.770 0.00 0.00

ATOM 868 H2 CuC X 1 15.040 5.260 29.170 0.00 0.00

ATOM 869 H2 CuC X 1 15.040 5.260 45.470 0.00 0.00

ATOM 870 H2 CuC X 1 15.040 5.260 61.770 0.00 0.00

ATOM 871 H2 CuC X 1 15.040 16.150 29.170 0.00 0.00

ATOM 872 H2 CuC X 1 15.040 16.150 45.470 0.00 0.00

ATOM 873 H2 CuC X 1 15.040 16.150 61.770 0.00 0.00

ATOM 874 H2 CuC X 1 15.040 27.040 29.170 0.00 0.00

ATOM 875 H2 CuC X 1 15.040 27.040 45.470 0.00 0.00

ATOM 876 H2 CuC X 1 15.040 27.040 61.770 0.00 0.00

ATOM 877 H2 CuC X 1 25.930 5.260 29.170 0.00 0.00

ATOM 878 H2 CuC X 1 25.930 5.260 45.470 0.00 0.00

ATOM 879 H2 CuC X 1 25.930 5.260 61.770 0.00 0.00

ATOM 880 H2 CuC X 1 25.930 16.150 29.170 0.00 0.00

ATOM 881 H2 CuC X 1 25.930 16.150 45.470 0.00 0.00

ATOM 882 H2 CuC X 1 25.930 16.150 61.770 0.00 0.00

ATOM 883 H2 CuC X 1 25.930 27.040 29.170 0.00 0.00

ATOM 884 H2 CuC X 1 25.930 27.040 45.470 0.00 0.00

ATOM 885 H2 CuC X 1 25.930 27.040 61.770 0.00 0.00

ATOM 886 H3 CuC X 1 4.190 5.310 31.700 0.00 0.00

ATOM 887 H3 CuC X 1 4.190 5.310 48.000 0.00 0.00

ATOM 888 H3 CuC X 1 4.190 5.310 64.300 0.00 0.00

ATOM 889 H3 CuC X 1 4.190 16.200 31.700 0.00 0.00

ATOM 890 H3 CuC X 1 4.190 16.200 48.000 0.00 0.00

ATOM 891 H3 CuC X 1 4.190 16.200 64.300 0.00 0.00

ATOM 892 H3 CuC X 1 4.190 27.090 31.700 0.00 0.00

ATOM 893 H3 CuC X 1 4.190 27.090 48.000 0.00 0.00

ATOM 894 H3 CuC X 1 4.190 27.090 64.300 0.00 0.00

ATOM 895 H3 CuC X 1 15.080 5.310 31.700 0.00 0.00

ATOM 896 H3 CuC X 1 15.080 5.310 48.000 0.00 0.00

ATOM 897 H3 CuC X 1 15.080 5.310 64.300 0.00 0.00

ATOM 898 H3 CuC X 1 15.080 16.200 31.700 0.00 0.00

ATOM 899 H3 CuC X 1 15.080 16.200 48.000 0.00 0.00

ATOM 900 H3 CuC X 1 15.080 16.200 64.300 0.00 0.00

ATOM 901 H3 CuC X 1 15.080 27.090 31.700 0.00 0.00

ATOM 902 H3 CuC X 1 15.080 27.090 48.000 0.00 0.00

ATOM 903 H3 CuC X 1 15.080 27.090 64.300 0.00 0.00

ATOM 904 H3 CuC X 1 25.970 5.310 31.700 0.00 0.00

ATOM	905	H3	CuC	X	1	25.970	5.310	48.000	0.00	0.00
ATOM	906	H3	CuC	X	1	25.970	5.310	64.300	0.00	0.00
ATOM	907	H3	CuC	X	1	25.970	16.200	31.700	0.00	0.00
ATOM	908	H3	CuC	X	1	25.970	16.200	48.000	0.00	0.00
ATOM	909	H3	CuC	X	1	25.970	16.200	64.300	0.00	0.00
ATOM	910	H3	CuC	X	1	25.970	27.090	31.700	0.00	0.00
ATOM	911	H3	CuC	X	1	25.970	27.090	48.000	0.00	0.00
ATOM	912	H3	CuC	X	1	25.970	27.090	64.300	0.00	0.00
ATOM	913	H3	CuC	X	1	3.300	9.590	31.430	0.00	0.00
ATOM	914	H3	CuC	X	1	3.300	9.590	47.740	0.00	0.00
ATOM	915	H3	CuC	X	1	3.300	9.590	64.040	0.00	0.00
ATOM	916	H3	CuC	X	1	3.300	20.480	31.430	0.00	0.00
ATOM	917	H3	CuC	X	1	3.300	20.480	47.740	0.00	0.00
ATOM	918	H3	CuC	X	1	3.300	20.480	64.040	0.00	0.00
ATOM	919	H3	CuC	X	1	14.190	9.590	31.430	0.00	0.00
ATOM	920	H3	CuC	X	1	14.190	9.590	47.740	0.00	0.00
ATOM	921	H3	CuC	X	1	14.190	9.590	64.040	0.00	0.00
ATOM	922	H3	CuC	X	1	14.190	20.480	31.430	0.00	0.00
ATOM	923	H3	CuC	X	1	14.190	20.480	47.740	0.00	0.00
ATOM	924	H3	CuC	X	1	14.190	20.480	64.040	0.00	0.00
ATOM	925	H3	CuC	X	1	25.080	9.590	31.430	0.00	0.00
ATOM	926	H3	CuC	X	1	25.080	9.590	47.740	0.00	0.00
ATOM	927	H3	CuC	X	1	25.080	9.590	64.040	0.00	0.00
ATOM	928	H3	CuC	X	1	25.080	20.480	31.430	0.00	0.00

ATOM 929 H3 CuC X 1 25.080 20.480 47.740 0.00 0.00

ATOM 930 H3 CuC X 1 25.080 20.480 64.040 0.00 0.00

ATOM 931 H2 CuC X 1 3.310 9.320 28.900 0.00 0.00

ATOM 932 H2 CuC X 1 3.310 9.320 45.200 0.00 0.00

ATOM 933 H2 CuC X 1 3.310 9.320 61.510 0.00 0.00

ATOM 934 H2 CuC X 1 3.310 20.210 28.900 0.00 0.00

ATOM 935 H2 CuC X 1 3.310 20.210 45.200 0.00 0.00

ATOM 936 H2 CuC X 1 3.310 20.210 61.510 0.00 0.00

ATOM 937 H2 CuC X 1 3.310 31.100 28.900 0.00 0.00

ATOM 938 H2 CuC X 1 3.310 31.100 45.200 0.00 0.00

ATOM 939 H2 CuC X 1 3.310 31.100 61.510 0.00 0.00

ATOM 940 H2 CuC X 1 14.200 9.320 28.900 0.00 0.00

ATOM 941 H2 CuC X 1 14.200 9.320 45.200 0.00 0.00

ATOM 942 H2 CuC X 1 14.200 9.320 61.510 0.00 0.00

ATOM 943 H2 CuC X 1 14.200 20.210 28.900 0.00 0.00

ATOM 944 H2 CuC X 1 14.200 20.210 45.200 0.00 0.00

ATOM 945 H2 CuC X 1 14.200 20.210 61.510 0.00 0.00

ATOM 946 H2 CuC X 1 14.200 31.100 28.900 0.00 0.00

ATOM 947 H2 CuC X 1 14.200 31.100 45.200 0.00 0.00

ATOM 948 H2 CuC X 1 14.200 31.100 61.510 0.00 0.00

ATOM 949 H2 CuC X 1 25.100 9.320 28.900 0.00 0.00

ATOM 950 H2 CuC X 1 25.100 9.320 45.200 0.00 0.00

ATOM 951 H2 CuC X 1 25.100 9.320 61.510 0.00 0.00

ATOM 952 H2 CuC X 1 25.100 20.210 28.900 0.00 0.00

ATOM 953 H2 CuC X 1 25.100 20.210 45.200 0.00 0.00

ATOM 954 H2 CuC X 1 25.100 20.210 61.510 0.00 0.00

ATOM 955 H2 CuC X 1 25.100 31.100 28.900 0.00 0.00

ATOM 956 H2 CuC X 1 25.100 31.100 45.200 0.00 0.00

ATOM 957 H2 CuC X 1 25.100 31.100 61.510 0.00 0.00

ATOM 958 H3 CuC X 1 3.050 8.820 35.680 0.00 0.00

ATOM 959 H3 CuC X 1 3.050 8.820 51.990 0.00 0.00

ATOM 960 H3 CuC X 1 3.050 8.820 68.290 0.00 0.00

ATOM 961 H3 CuC X 1 3.050 19.710 35.680 0.00 0.00

ATOM 962 H3 CuC X 1 3.050 19.710 51.990 0.00 0.00

ATOM 963 H3 CuC X 1 3.050 19.710 68.290 0.00 0.00

ATOM 964 H3 CuC X 1 3.050 30.600 35.680 0.00 0.00

ATOM 965 H3 CuC X 1 3.050 30.600 51.990 0.00 0.00

ATOM 966 H3 CuC X 1 3.050 30.600 68.290 0.00 0.00

ATOM 967 H3 CuC X 1 13.940 8.820 35.680 0.00 0.00

ATOM 968 H3 CuC X 1 13.940 8.820 51.990 0.00 0.00

ATOM 969 H3 CuC X 1 13.940 8.820 68.290 0.00 0.00

ATOM 970 H3 CuC X 1 13.940 19.710 35.680 0.00 0.00

ATOM 971 H3 CuC X 1 13.940 19.710 51.990 0.00 0.00

ATOM 972 H3 CuC X 1 13.940 19.710 68.290 0.00 0.00

ATOM 973 H3 CuC X 1 13.940 30.600 35.680 0.00 0.00

ATOM 974 H3 CuC X 1 13.940 30.600 51.990 0.00 0.00

ATOM 975 H3 CuC X 1 13.940 30.600 68.290 0.00 0.00

ATOM 976 H3 CuC X 1 24.830 8.820 35.680 0.00 0.00

ATOM 977 H3 CuC X 1 24.830 8.820 51.990 0.00 0.00

ATOM 978 H3 CuC X 1 24.830 8.820 68.290 0.00 0.00

ATOM 979 H3 CuC X 1 24.830 19.710 35.680 0.00 0.00

ATOM 980 H3 CuC X 1 24.830 19.710 51.990 0.00 0.00

ATOM 981 H3 CuC X 1 24.830 19.710 68.290 0.00 0.00

ATOM 982 H3 CuC X 1 24.830 30.600 35.680 0.00 0.00

ATOM 983 H3 CuC X 1 24.830 30.600 51.990 0.00 0.00

ATOM 984 H3 CuC X 1 24.830 30.600 68.290 0.00 0.00

ATOM 985 H2 CuC X 1 2.990 8.880 38.210 0.00 0.00

ATOM 986 H2 CuC X 1 2.990 8.880 54.510 0.00 0.00

ATOM 987 H2 CuC X 1 2.990 8.880 70.810 0.00 0.00

ATOM 988 H2 CuC X 1 2.990 19.770 38.210 0.00 0.00

ATOM 989 H2 CuC X 1 2.990 19.770 54.510 0.00 0.00

ATOM 990 H2 CuC X 1 2.990 19.770 70.810 0.00 0.00

ATOM 991 H2 CuC X 1 2.990 30.660 38.210 0.00 0.00

ATOM 992 H2 CuC X 1 2.990 30.660 54.510 0.00 0.00

ATOM 993 H2 CuC X 1 2.990 30.660 70.810 0.00 0.00

ATOM 994 H2 CuC X 1 13.880 8.880 38.210 0.00 0.00

ATOM 995 H2 CuC X 1 13.880 8.880 54.510 0.00 0.00

ATOM 996 H2 CuC X 1 13.880 8.880 70.810 0.00 0.00

ATOM 997 H2 CuC X 1 13.880 19.770 38.210 0.00 0.00

ATOM 998 H2 CuC X 1 13.880 19.770 54.510 0.00 0.00

ATOM 999 H2 CuC X 1 13.880 19.770 70.810 0.00 0.00

ATOM 1000 H2 CuC X 1 13.880 30.660 38.210 0.00 0.00

ATOM	1001	H2	CuC	X	1	13.880	30.660	54.510	0.00	0.00
ATOM	1002	H2	CuC	X	1	13.880	30.660	70.810	0.00	0.00
ATOM	1003	H2	CuC	X	1	24.770	8.880	38.210	0.00	0.00
ATOM	1004	H2	CuC	X	1	24.770	8.880	54.510	0.00	0.00
ATOM	1005	H2	CuC	X	1	24.770	8.880	70.810	0.00	0.00
ATOM	1006	H2	CuC	X	1	24.770	19.770	38.210	0.00	0.00
ATOM	1007	H2	CuC	X	1	24.770	19.770	54.510	0.00	0.00
ATOM	1008	H2	CuC	X	1	24.770	19.770	70.810	0.00	0.00
ATOM	1009	H2	CuC	X	1	24.770	30.660	38.210	0.00	0.00
ATOM	1010	H2	CuC	X	1	24.770	30.660	54.510	0.00	0.00
ATOM	1011	H2	CuC	X	1	24.770	30.660	70.810	0.00	0.00
ATOM	1012	H2	CuC	X	1	4.560	5.050	38.540	0.00	0.00
ATOM	1013	H2	CuC	X	1	4.560	5.050	54.840	0.00	0.00
ATOM	1014	H2	CuC	X	1	4.560	5.050	71.140	0.00	0.00
ATOM	1015	H2	CuC	X	1	4.560	15.940	38.540	0.00	0.00
ATOM	1016	H2	CuC	X	1	4.560	15.940	54.840	0.00	0.00
ATOM	1017	H2	CuC	X	1	4.560	15.940	71.140	0.00	0.00
ATOM	1018	H2	CuC	X	1	4.560	26.830	38.540	0.00	0.00
ATOM	1019	H2	CuC	X	1	4.560	26.830	54.840	0.00	0.00
ATOM	1020	H2	CuC	X	1	4.560	26.830	71.140	0.00	0.00
ATOM	1021	H2	CuC	X	1	15.450	5.050	38.540	0.00	0.00
ATOM	1022	H2	CuC	X	1	15.450	5.050	54.840	0.00	0.00
ATOM	1023	H2	CuC	X	1	15.450	5.050	71.140	0.00	0.00
ATOM	1024	H2	CuC	X	1	15.450	15.940	38.540	0.00	0.00

ATOM	1025	H2	CuC	X	1	15.450	15.940	54.840	0.00	0.00
ATOM	1026	H2	CuC	X	1	15.450	15.940	71.140	0.00	0.00
ATOM	1027	H2	CuC	X	1	15.450	26.830	38.540	0.00	0.00
ATOM	1028	H2	CuC	X	1	15.450	26.830	54.840	0.00	0.00
ATOM	1029	H2	CuC	X	1	15.450	26.830	71.140	0.00	0.00
ATOM	1030	H2	CuC	X	1	26.340	5.050	38.540	0.00	0.00
ATOM	1031	H2	CuC	X	1	26.340	5.050	54.840	0.00	0.00
ATOM	1032	H2	CuC	X	1	26.340	5.050	71.140	0.00	0.00
ATOM	1033	H2	CuC	X	1	26.340	15.940	38.540	0.00	0.00
ATOM	1034	H2	CuC	X	1	26.340	15.940	54.840	0.00	0.00
ATOM	1035	H2	CuC	X	1	26.340	15.940	71.140	0.00	0.00
ATOM	1036	H2	CuC	X	1	26.340	26.830	38.540	0.00	0.00
ATOM	1037	H2	CuC	X	1	26.340	26.830	54.840	0.00	0.00
ATOM	1038	H2	CuC	X	1	26.340	26.830	71.140	0.00	0.00
ATOM	1039	H3	CuC	X	1	4.740	4.800	36.000	0.00	0.00
ATOM	1040	H3	CuC	X	1	4.740	4.800	52.310	0.00	0.00
ATOM	1041	H3	CuC	X	1	4.740	4.800	68.610	0.00	0.00
ATOM	1042	H3	CuC	X	1	4.740	15.690	36.000	0.00	0.00
ATOM	1043	H3	CuC	X	1	4.740	15.690	52.310	0.00	0.00
ATOM	1044	H3	CuC	X	1	4.740	15.690	68.610	0.00	0.00
ATOM	1045	H3	CuC	X	1	4.740	26.580	36.000	0.00	0.00
ATOM	1046	H3	CuC	X	1	4.740	26.580	52.310	0.00	0.00
ATOM	1047	H3	CuC	X	1	4.740	26.580	68.610	0.00	0.00
ATOM	1048	H3	CuC	X	1	15.630	4.800	36.000	0.00	0.00

ATOM	1049	H3	CuC	X	1	15.630	4.800	52.310	0.00	0.00
ATOM	1050	H3	CuC	X	1	15.630	4.800	68.610	0.00	0.00
ATOM	1051	H3	CuC	X	1	15.630	15.690	36.000	0.00	0.00
ATOM	1052	H3	CuC	X	1	15.630	15.690	52.310	0.00	0.00
ATOM	1053	H3	CuC	X	1	15.630	15.690	68.610	0.00	0.00
ATOM	1054	H3	CuC	X	1	15.630	26.580	36.000	0.00	0.00
ATOM	1055	H3	CuC	X	1	15.630	26.580	52.310	0.00	0.00
ATOM	1056	H3	CuC	X	1	15.630	26.580	68.610	0.00	0.00
ATOM	1057	H3	CuC	X	1	26.520	4.800	36.000	0.00	0.00
ATOM	1058	H3	CuC	X	1	26.520	4.800	52.310	0.00	0.00
ATOM	1059	H3	CuC	X	1	26.520	4.800	68.610	0.00	0.00
ATOM	1060	H3	CuC	X	1	26.520	15.690	36.000	0.00	0.00
ATOM	1061	H3	CuC	X	1	26.520	15.690	52.310	0.00	0.00
ATOM	1062	H3	CuC	X	1	26.520	15.690	68.610	0.00	0.00
ATOM	1063	H3	CuC	X	1	26.520	26.580	36.000	0.00	0.00
ATOM	1064	H3	CuC	X	1	26.520	26.580	52.310	0.00	0.00
ATOM	1065	H3	CuC	X	1	26.520	26.580	68.610	0.00	0.00
ATOM	1066	H1	CuC	X	1	7.910	7.170	27.650	0.00	0.00
ATOM	1067	H1	CuC	X	1	7.910	7.170	43.950	0.00	0.00
ATOM	1068	H1	CuC	X	1	7.910	7.170	60.260	0.00	0.00
ATOM	1069	H1	CuC	X	1	7.910	7.170	76.560	0.00	0.00
ATOM	1070	H1	CuC	X	1	7.910	18.060	27.650	0.00	0.00
ATOM	1071	H1	CuC	X	1	7.910	18.060	43.950	0.00	0.00
ATOM	1072	H1	CuC	X	1	7.910	18.060	60.260	0.00	0.00

ATOM	1073	H1	CuC	X	1	7.910	18.060	76.560	0.00	0.00
ATOM	1074	H1	CuC	X	1	7.910	28.950	27.650	0.00	0.00
ATOM	1075	H1	CuC	X	1	7.910	28.950	43.950	0.00	0.00
ATOM	1076	H1	CuC	X	1	7.910	28.950	60.260	0.00	0.00
ATOM	1077	H1	CuC	X	1	7.910	28.950	76.560	0.00	0.00
ATOM	1078	H1	CuC	X	1	18.800	7.170	27.650	0.00	0.00
ATOM	1079	H1	CuC	X	1	18.800	7.170	43.950	0.00	0.00
ATOM	1080	H1	CuC	X	1	18.800	7.170	60.260	0.00	0.00
ATOM	1081	H1	CuC	X	1	18.800	7.170	76.560	0.00	0.00
ATOM	1082	H1	CuC	X	1	18.800	18.060	27.650	0.00	0.00
ATOM	1083	H1	CuC	X	1	18.800	18.060	43.950	0.00	0.00
ATOM	1084	H1	CuC	X	1	18.800	18.060	60.260	0.00	0.00
ATOM	1085	H1	CuC	X	1	18.800	18.060	76.560	0.00	0.00
ATOM	1086	H1	CuC	X	1	18.800	28.950	27.650	0.00	0.00
ATOM	1087	H1	CuC	X	1	18.800	28.950	43.950	0.00	0.00
ATOM	1088	H1	CuC	X	1	18.800	28.950	60.260	0.00	0.00
ATOM	1089	H1	CuC	X	1	18.800	28.950	76.560	0.00	0.00
ATOM	1090	H1	CuC	X	1	29.690	7.170	27.650	0.00	0.00
ATOM	1091	H1	CuC	X	1	29.690	7.170	43.950	0.00	0.00
ATOM	1092	H1	CuC	X	1	29.690	7.170	60.260	0.00	0.00
ATOM	1093	H1	CuC	X	1	29.690	7.170	76.560	0.00	0.00
ATOM	1094	H1	CuC	X	1	29.690	18.060	27.650	0.00	0.00
ATOM	1095	H1	CuC	X	1	29.690	18.060	43.950	0.00	0.00
ATOM	1096	H1	CuC	X	1	29.690	18.060	60.260	0.00	0.00

ATOM 1097 H1 CuC X 1 29.690 18.060 76.560 0.00 0.00

ATOM 1098 H1 CuC X 1 29.690 28.950 27.650 0.00 0.00

ATOM 1099 H1 CuC X 1 29.690 28.950 43.950 0.00 0.00

ATOM 1100 H1 CuC X 1 29.690 28.950 60.260 0.00 0.00

ATOM 1101 H1 CuC X 1 29.690 28.950 76.560 0.00 0.00

ATOM 1102 H1 CuC X 1 7.910 7.050 23.320 0.00 0.00

ATOM 1103 H1 CuC X 1 7.910 7.050 39.620 0.00 0.00

ATOM 1104 H1 CuC X 1 7.910 7.050 55.930 0.00 0.00

ATOM 1105 H1 CuC X 1 7.910 7.050 72.230 0.00 0.00

ATOM 1106 H1 CuC X 1 7.910 17.940 23.320 0.00 0.00

ATOM 1107 H1 CuC X 1 7.910 17.940 39.620 0.00 0.00

ATOM 1108 H1 CuC X 1 7.910 17.940 55.930 0.00 0.00

ATOM 1109 H1 CuC X 1 7.910 17.940 72.230 0.00 0.00

ATOM 1110 H1 CuC X 1 7.910 28.830 23.320 0.00 0.00

ATOM 1111 H1 CuC X 1 7.910 28.830 39.620 0.00 0.00

ATOM 1112 H1 CuC X 1 7.910 28.830 55.930 0.00 0.00

ATOM 1113 H1 CuC X 1 7.910 28.830 72.230 0.00 0.00

ATOM 1114 H1 CuC X 1 18.800 7.050 23.320 0.00 0.00

ATOM 1115 H1 CuC X 1 18.800 7.050 39.620 0.00 0.00

ATOM 1116 H1 CuC X 1 18.800 7.050 55.930 0.00 0.00

ATOM 1117 H1 CuC X 1 18.800 7.050 72.230 0.00 0.00

ATOM 1118 H1 CuC X 1 18.800 17.940 23.320 0.00 0.00

ATOM 1119 H1 CuC X 1 18.800 17.940 39.620 0.00 0.00

ATOM 1120 H1 CuC X 1 18.800 17.940 55.930 0.00 0.00

ATOM	1121	H1	CuC	X	1	18.800	17.940	72.230	0.00	0.00
ATOM	1122	H1	CuC	X	1	18.800	28.830	23.320	0.00	0.00
ATOM	1123	H1	CuC	X	1	18.800	28.830	39.620	0.00	0.00
ATOM	1124	H1	CuC	X	1	18.800	28.830	55.930	0.00	0.00
ATOM	1125	H1	CuC	X	1	18.800	28.830	72.230	0.00	0.00
ATOM	1126	H1	CuC	X	1	29.690	7.050	23.320	0.00	0.00
ATOM	1127	H1	CuC	X	1	29.690	7.050	39.620	0.00	0.00
ATOM	1128	H1	CuC	X	1	29.690	7.050	55.930	0.00	0.00
ATOM	1129	H1	CuC	X	1	29.690	7.050	72.230	0.00	0.00
ATOM	1130	H1	CuC	X	1	29.690	17.940	23.320	0.00	0.00
ATOM	1131	H1	CuC	X	1	29.690	17.940	39.620	0.00	0.00
ATOM	1132	H1	CuC	X	1	29.690	17.940	55.930	0.00	0.00
ATOM	1133	H1	CuC	X	1	29.690	17.940	72.230	0.00	0.00
ATOM	1134	H1	CuC	X	1	29.690	28.830	23.320	0.00	0.00
ATOM	1135	H1	CuC	X	1	29.690	28.830	39.620	0.00	0.00
ATOM	1136	H1	CuC	X	1	29.690	28.830	55.930	0.00	0.00
ATOM	1137	H1	CuC	X	1	29.690	28.830	72.230	0.00	0.00
ATOM	1138	H1	CuC	X	1	10.460	7.090	23.320	0.00	0.00
ATOM	1139	H1	CuC	X	1	10.460	7.090	39.620	0.00	0.00
ATOM	1140	H1	CuC	X	1	10.460	7.090	55.920	0.00	0.00
ATOM	1141	H1	CuC	X	1	10.460	7.090	72.230	0.00	0.00
ATOM	1142	H1	CuC	X	1	10.460	17.980	23.320	0.00	0.00
ATOM	1143	H1	CuC	X	1	10.460	17.980	39.620	0.00	0.00
ATOM	1144	H1	CuC	X	1	10.460	17.980	55.920	0.00	0.00

ATOM	1145	H1	CuC	X	1	10.460	17.980	72.230	0.00	0.00
ATOM	1146	H1	CuC	X	1	10.460	28.870	23.320	0.00	0.00
ATOM	1147	H1	CuC	X	1	10.460	28.870	39.620	0.00	0.00
ATOM	1148	H1	CuC	X	1	10.460	28.870	55.920	0.00	0.00
ATOM	1149	H1	CuC	X	1	10.460	28.870	72.230	0.00	0.00
ATOM	1150	H1	CuC	X	1	21.350	7.090	23.320	0.00	0.00
ATOM	1151	H1	CuC	X	1	21.350	7.090	39.620	0.00	0.00
ATOM	1152	H1	CuC	X	1	21.350	7.090	55.920	0.00	0.00
ATOM	1153	H1	CuC	X	1	21.350	7.090	72.230	0.00	0.00
ATOM	1154	H1	CuC	X	1	21.350	17.980	23.320	0.00	0.00
ATOM	1155	H1	CuC	X	1	21.350	17.980	39.620	0.00	0.00
ATOM	1156	H1	CuC	X	1	21.350	17.980	55.920	0.00	0.00
ATOM	1157	H1	CuC	X	1	21.350	17.980	72.230	0.00	0.00
ATOM	1158	H1	CuC	X	1	21.350	28.870	23.320	0.00	0.00
ATOM	1159	H1	CuC	X	1	21.350	28.870	39.620	0.00	0.00
ATOM	1160	H1	CuC	X	1	21.350	28.870	55.920	0.00	0.00
ATOM	1161	H1	CuC	X	1	21.350	28.870	72.230	0.00	0.00
ATOM	1162	H1	CuC	X	1	32.250	7.090	23.320	0.00	0.00
ATOM	1163	H1	CuC	X	1	32.250	7.090	39.620	0.00	0.00
ATOM	1164	H1	CuC	X	1	32.250	7.090	55.920	0.00	0.00
ATOM	1165	H1	CuC	X	1	32.250	7.090	72.230	0.00	0.00
ATOM	1166	H1	CuC	X	1	32.250	17.980	23.320	0.00	0.00
ATOM	1167	H1	CuC	X	1	32.250	17.980	39.620	0.00	0.00
ATOM	1168	H1	CuC	X	1	32.250	17.980	55.920	0.00	0.00

ATOM	1169	H1	CuC	X	1	32.250	17.980	72.230	0.00	0.00
ATOM	1170	H1	CuC	X	1	32.250	28.870	23.320	0.00	0.00
ATOM	1171	H1	CuC	X	1	32.250	28.870	39.620	0.00	0.00
ATOM	1172	H1	CuC	X	1	32.250	28.870	55.920	0.00	0.00
ATOM	1173	H1	CuC	X	1	32.250	28.870	72.230	0.00	0.00
ATOM	1174	H1	CuC	X	1	10.470	7.120	27.650	0.00	0.00
ATOM	1175	H1	CuC	X	1	10.470	7.120	43.950	0.00	0.00
ATOM	1176	H1	CuC	X	1	10.470	7.120	60.260	0.00	0.00
ATOM	1177	H1	CuC	X	1	10.470	7.120	76.560	0.00	0.00
ATOM	1178	H1	CuC	X	1	10.470	18.010	27.650	0.00	0.00
ATOM	1179	H1	CuC	X	1	10.470	18.010	43.950	0.00	0.00
ATOM	1180	H1	CuC	X	1	10.470	18.010	60.260	0.00	0.00
ATOM	1181	H1	CuC	X	1	10.470	18.010	76.560	0.00	0.00
ATOM	1182	H1	CuC	X	1	10.470	28.900	27.650	0.00	0.00
ATOM	1183	H1	CuC	X	1	10.470	28.900	43.950	0.00	0.00
ATOM	1184	H1	CuC	X	1	10.470	28.900	60.260	0.00	0.00
ATOM	1185	H1	CuC	X	1	10.470	28.900	76.560	0.00	0.00
ATOM	1186	H1	CuC	X	1	21.360	7.120	27.650	0.00	0.00
ATOM	1187	H1	CuC	X	1	21.360	7.120	43.950	0.00	0.00
ATOM	1188	H1	CuC	X	1	21.360	7.120	60.260	0.00	0.00
ATOM	1189	H1	CuC	X	1	21.360	7.120	76.560	0.00	0.00
ATOM	1190	H1	CuC	X	1	21.360	18.010	27.650	0.00	0.00
ATOM	1191	H1	CuC	X	1	21.360	18.010	43.950	0.00	0.00
ATOM	1192	H1	CuC	X	1	21.360	18.010	60.260	0.00	0.00

ATOM 1193 H1 CuC X 1 21.360 18.010 76.560 0.00 0.00

ATOM 1194 H1 CuC X 1 21.360 28.900 27.650 0.00 0.00

ATOM 1195 H1 CuC X 1 21.360 28.900 43.950 0.00 0.00

ATOM 1196 H1 CuC X 1 21.360 28.900 60.260 0.00 0.00

ATOM 1197 H1 CuC X 1 21.360 28.900 76.560 0.00 0.00

ATOM 1198 H1 CuC X 1 32.250 7.120 27.650 0.00 0.00

ATOM 1199 H1 CuC X 1 32.250 7.120 43.950 0.00 0.00

ATOM 1200 H1 CuC X 1 32.250 7.120 60.260 0.00 0.00

ATOM 1201 H1 CuC X 1 32.250 7.120 76.560 0.00 0.00

ATOM 1202 H1 CuC X 1 32.250 18.010 27.650 0.00 0.00

ATOM 1203 H1 CuC X 1 32.250 18.010 43.950 0.00 0.00

ATOM 1204 H1 CuC X 1 32.250 18.010 60.260 0.00 0.00

ATOM 1205 H1 CuC X 1 32.250 18.010 76.560 0.00 0.00

ATOM 1206 H1 CuC X 1 32.250 28.900 27.650 0.00 0.00

ATOM 1207 H1 CuC X 1 32.250 28.900 43.950 0.00 0.00

ATOM 1208 H1 CuC X 1 32.250 28.900 60.260 0.00 0.00

ATOM 1209 H1 CuC X 1 32.250 28.900 76.560 0.00 0.00

ATOM 1210 O CuC X 1 3.430 5.140 24.530 0.00 0.00

ATOM 1211 O CuC X 1 3.430 5.140 40.840 0.00 0.00

ATOM 1212 O CuC X 1 3.430 5.140 57.140 0.00 0.00

ATOM 1213 O CuC X 1 3.430 5.140 73.440 0.00 0.00

ATOM 1214 O CuC X 1 3.430 16.030 24.530 0.00 0.00

ATOM 1215 O CuC X 1 3.430 16.030 40.840 0.00 0.00

ATOM 1216 O CuC X 1 3.430 16.030 57.140 0.00 0.00

ATOM 1217 O CuC X 1 3.430 16.030 73.440 0.00 0.00

ATOM 1218 O CuC X 1 3.430 26.920 24.530 0.00 0.00

ATOM 1219 O CuC X 1 3.430 26.920 40.840 0.00 0.00

ATOM 1220 O CuC X 1 3.430 26.920 57.140 0.00 0.00

ATOM 1221 O CuC X 1 3.430 26.920 73.440 0.00 0.00

ATOM 1222 O CuC X 1 14.320 5.140 24.530 0.00 0.00

ATOM 1223 O CuC X 1 14.320 5.140 40.840 0.00 0.00

ATOM 1224 O CuC X 1 14.320 5.140 57.140 0.00 0.00

ATOM 1225 O CuC X 1 14.320 5.140 73.440 0.00 0.00

ATOM 1226 O CuC X 1 14.320 16.030 24.530 0.00 0.00

ATOM 1227 O CuC X 1 14.320 16.030 40.840 0.00 0.00

ATOM 1228 O CuC X 1 14.320 16.030 57.140 0.00 0.00

ATOM 1229 O CuC X 1 14.320 16.030 73.440 0.00 0.00

ATOM 1230 O CuC X 1 14.320 26.920 24.530 0.00 0.00

ATOM 1231 O CuC X 1 14.320 26.920 40.840 0.00 0.00

ATOM 1232 O CuC X 1 14.320 26.920 57.140 0.00 0.00

ATOM 1233 O CuC X 1 14.320 26.920 73.440 0.00 0.00

ATOM 1234 O CuC X 1 25.210 5.140 24.530 0.00 0.00

ATOM 1235 O CuC X 1 25.210 5.140 40.840 0.00 0.00

ATOM 1236 O CuC X 1 25.210 5.140 57.140 0.00 0.00

ATOM 1237 O CuC X 1 25.210 5.140 73.440 0.00 0.00

ATOM 1238 O CuC X 1 25.210 16.030 24.530 0.00 0.00

ATOM 1239 O CuC X 1 25.210 16.030 40.840 0.00 0.00

ATOM 1240 O CuC X 1 25.210 16.030 57.140 0.00 0.00

ATOM 1241 O CuC X 1 25.210 16.030 73.440 0.00 0.00

ATOM 1242 O CuC X 1 25.210 26.920 24.530 0.00 0.00

ATOM 1243 O CuC X 1 25.210 26.920 40.840 0.00 0.00

ATOM 1244 O CuC X 1 25.210 26.920 57.140 0.00 0.00

ATOM 1245 O CuC X 1 25.210 26.920 73.440 0.00 0.00

ATOM 1246 O CuC X 1 4.090 5.170 26.730 0.00 0.00

ATOM 1247 O CuC X 1 4.090 5.170 43.030 0.00 0.00

ATOM 1248 O CuC X 1 4.090 5.170 59.340 0.00 0.00

ATOM 1249 O CuC X 1 4.090 5.170 75.640 0.00 0.00

ATOM 1250 O CuC X 1 4.090 16.060 26.730 0.00 0.00

ATOM 1251 O CuC X 1 4.090 16.060 43.030 0.00 0.00

ATOM 1252 O CuC X 1 4.090 16.060 59.340 0.00 0.00

ATOM 1253 O CuC X 1 4.090 16.060 75.640 0.00 0.00

ATOM 1254 O CuC X 1 4.090 26.950 26.730 0.00 0.00

ATOM 1255 O CuC X 1 4.090 26.950 43.030 0.00 0.00

ATOM 1256 O CuC X 1 4.090 26.950 59.340 0.00 0.00

ATOM 1257 O CuC X 1 4.090 26.950 75.640 0.00 0.00

ATOM 1258 O CuC X 1 14.990 5.170 26.730 0.00 0.00

ATOM 1259 O CuC X 1 14.990 5.170 43.030 0.00 0.00

ATOM 1260 O CuC X 1 14.990 5.170 59.340 0.00 0.00

ATOM 1261 O CuC X 1 14.990 5.170 75.640 0.00 0.00

ATOM 1262 O CuC X 1 14.990 16.060 26.730 0.00 0.00

ATOM 1263 O CuC X 1 14.990 16.060 43.030 0.00 0.00

ATOM 1264 O CuC X 1 14.990 16.060 59.340 0.00 0.00

```
ATOM   1265  O   CuC X   1      14.990  16.060  75.640  0.00  0.00
ATOM   1266  O   CuC X   1      14.990  26.950  26.730  0.00  0.00
ATOM   1267  O   CuC X   1      14.990  26.950  43.030  0.00  0.00
ATOM   1268  O   CuC X   1      14.990  26.950  59.340  0.00  0.00
ATOM   1269  O   CuC X   1      14.990  26.950  75.640  0.00  0.00
ATOM   1270  O   CuC X   1      25.880   5.170  26.730  0.00  0.00
ATOM   1271  O   CuC X   1      25.880   5.170  43.030  0.00  0.00
ATOM   1272  O   CuC X   1      25.880   5.170  59.340  0.00  0.00
ATOM   1273  O   CuC X   1      25.880   5.170  75.640  0.00  0.00
ATOM   1274  O   CuC X   1      25.880  16.060  26.730  0.00  0.00
ATOM   1275  O   CuC X   1      25.880  16.060  43.030  0.00  0.00
ATOM   1276  O   CuC X   1      25.880  16.060  59.340  0.00  0.00
ATOM   1277  O   CuC X   1      25.880  16.060  75.640  0.00  0.00
ATOM   1278  O   CuC X   1      25.880  26.950  26.730  0.00  0.00
ATOM   1279  O   CuC X   1      25.880  26.950  43.030  0.00  0.00
ATOM   1280  O   CuC X   1      25.880  26.950  59.340  0.00  0.00
ATOM   1281  O   CuC X   1      25.880  26.950  75.640  0.00  0.00
ATOM   1282  O   CuC X   1       3.400   9.110  26.600  0.00  0.00
ATOM   1283  O   CuC X   1       3.400   9.110  42.910  0.00  0.00
ATOM   1284  O   CuC X   1       3.400   9.110  59.210  0.00  0.00
ATOM   1285  O   CuC X   1       3.400   9.110  75.510  0.00  0.00
ATOM   1286  O   CuC X   1       3.400  20.000  26.600  0.00  0.00
ATOM   1287  O   CuC X   1       3.400  20.000  42.910  0.00  0.00
ATOM   1288  O   CuC X   1       3.400  20.000  59.210  0.00  0.00
```

ATOM	1289	O	CuC	X	1	3.400	20.000	75.510	0.00	0.00
ATOM	1290	O	CuC	X	1	14.290	9.110	26.600	0.00	0.00
ATOM	1291	O	CuC	X	1	14.290	9.110	42.910	0.00	0.00
ATOM	1292	O	CuC	X	1	14.290	9.110	59.210	0.00	0.00
ATOM	1293	O	CuC	X	1	14.290	9.110	75.510	0.00	0.00
ATOM	1294	O	CuC	X	1	14.290	20.000	26.600	0.00	0.00
ATOM	1295	O	CuC	X	1	14.290	20.000	42.910	0.00	0.00
ATOM	1296	O	CuC	X	1	14.290	20.000	59.210	0.00	0.00
ATOM	1297	O	CuC	X	1	14.290	20.000	75.510	0.00	0.00
ATOM	1298	O	CuC	X	1	25.180	9.110	26.600	0.00	0.00
ATOM	1299	O	CuC	X	1	25.180	9.110	42.910	0.00	0.00
ATOM	1300	O	CuC	X	1	25.180	9.110	59.210	0.00	0.00
ATOM	1301	O	CuC	X	1	25.180	9.110	75.510	0.00	0.00
ATOM	1302	O	CuC	X	1	25.180	20.000	26.600	0.00	0.00
ATOM	1303	O	CuC	X	1	25.180	20.000	42.910	0.00	0.00
ATOM	1304	O	CuC	X	1	25.180	20.000	59.210	0.00	0.00
ATOM	1305	O	CuC	X	1	25.180	20.000	75.510	0.00	0.00
ATOM	1306	O	CuC	X	1	4.040	9.120	24.400	0.00	0.00
ATOM	1307	O	CuC	X	1	4.040	9.120	40.710	0.00	0.00
ATOM	1308	O	CuC	X	1	4.040	9.120	57.010	0.00	0.00
ATOM	1309	O	CuC	X	1	4.040	9.120	73.310	0.00	0.00
ATOM	1310	O	CuC	X	1	4.040	20.010	24.400	0.00	0.00
ATOM	1311	O	CuC	X	1	4.040	20.010	40.710	0.00	0.00
ATOM	1312	O	CuC	X	1	4.040	20.010	57.010	0.00	0.00

ATOM 1313 O CuC X 1 4.040 20.010 73.310 0.00 0.00

ATOM 1314 O CuC X 1 14.940 9.120 24.400 0.00 0.00

ATOM 1315 O CuC X 1 14.940 9.120 40.710 0.00 0.00

ATOM 1316 O CuC X 1 14.940 9.120 57.010 0.00 0.00

ATOM 1317 O CuC X 1 14.940 9.120 73.310 0.00 0.00

ATOM 1318 O CuC X 1 14.940 20.010 24.400 0.00 0.00

ATOM 1319 O CuC X 1 14.940 20.010 40.710 0.00 0.00

ATOM 1320 O CuC X 1 14.940 20.010 57.010 0.00 0.00

ATOM 1321 O CuC X 1 14.940 20.010 73.310 0.00 0.00

ATOM 1322 O CuC X 1 25.830 9.120 24.400 0.00 0.00

ATOM 1323 O CuC X 1 25.830 9.120 40.710 0.00 0.00

ATOM 1324 O CuC X 1 25.830 9.120 57.010 0.00 0.00

ATOM 1325 O CuC X 1 25.830 9.120 73.310 0.00 0.00

ATOM 1326 O CuC X 1 25.830 20.010 24.400 0.00 0.00

ATOM 1327 O CuC X 1 25.830 20.010 40.710 0.00 0.00

ATOM 1328 O CuC X 1 25.830 20.010 57.010 0.00 0.00

ATOM 1329 O CuC X 1 25.830 20.010 73.310 0.00 0.00

ATOM 1330 C1 CuC X 1 3.730 31.450 25.510 0.00 0.00

ATOM 1331 C1 CuC X 1 3.730 31.450 41.810 0.00 0.00

ATOM 1332 C1 CuC X 1 3.730 31.450 58.110 0.00 0.00

ATOM 1333 C1 CuC X 1 3.730 31.450 74.410 0.00 0.00

ATOM 1334 C1 CuC X 1 3.730 9.670 25.510 0.00 0.00

ATOM 1335 C1 CuC X 1 3.730 9.670 41.810 0.00 0.00

ATOM 1336 C1 CuC X 1 3.730 9.670 58.110 0.00 0.00

```
ATOM   1337  C1  CuC X   1       3.730   9.670  74.410  0.00  0.00
ATOM   1338  C1  CuC X   1       3.730  20.560  25.510  0.00  0.00
ATOM   1339  C1  CuC X   1       3.730  20.560  41.810  0.00  0.00
ATOM   1340  C1  CuC X   1       3.730  20.560  58.110  0.00  0.00
ATOM   1341  C1  CuC X   1       3.730  20.560  74.410  0.00  0.00
ATOM   1342  C1  CuC X   1      14.620  31.450  25.510  0.00  0.00
ATOM   1343  C1  CuC X   1      14.620  31.450  41.810  0.00  0.00
ATOM   1344  C1  CuC X   1      14.620  31.450  58.110  0.00  0.00
ATOM   1345  C1  CuC X   1      14.620  31.450  74.410  0.00  0.00
ATOM   1346  C1  CuC X   1      14.620   9.670  25.510  0.00  0.00
ATOM   1347  C1  CuC X   1      14.620   9.670  41.810  0.00  0.00
ATOM   1348  C1  CuC X   1      14.620   9.670  58.110  0.00  0.00
ATOM   1349  C1  CuC X   1      14.620   9.670  74.410  0.00  0.00
ATOM   1350  C1  CuC X   1      14.620  20.560  25.510  0.00  0.00
ATOM   1351  C1  CuC X   1      14.620  20.560  41.810  0.00  0.00
ATOM   1352  C1  CuC X   1      14.620  20.560  58.110  0.00  0.00
ATOM   1353  C1  CuC X   1      14.620  20.560  74.410  0.00  0.00
ATOM   1354  C1  CuC X   1      25.510  31.450  25.510  0.00  0.00
ATOM   1355  C1  CuC X   1      25.510  31.450  41.810  0.00  0.00
ATOM   1356  C1  CuC X   1      25.510  31.450  58.110  0.00  0.00
ATOM   1357  C1  CuC X   1      25.510  31.450  74.410  0.00  0.00
ATOM   1358  C1  CuC X   1      25.510   9.670  25.510  0.00  0.00
ATOM   1359  C1  CuC X   1      25.510   9.670  41.810  0.00  0.00
ATOM   1360  C1  CuC X   1      25.510   9.670  58.110  0.00  0.00
```

ATOM	1361	C1	CuC	X	1	25.510	9.670	74.410	0.00	0.00
ATOM	1362	C1	CuC	X	1	25.510	20.560	25.510	0.00	0.00
ATOM	1363	C1	CuC	X	1	25.510	20.560	41.810	0.00	0.00
ATOM	1364	C1	CuC	X	1	25.510	20.560	58.110	0.00	0.00
ATOM	1365	C1	CuC	X	1	25.510	20.560	74.410	0.00	0.00
ATOM	1366	C2	CuC	X	1	3.770	3.090	25.630	0.00	0.00
ATOM	1367	C2	CuC	X	1	3.770	3.090	41.930	0.00	0.00
ATOM	1368	C2	CuC	X	1	3.770	3.090	58.240	0.00	0.00
ATOM	1369	C2	CuC	X	1	3.770	3.090	74.540	0.00	0.00
ATOM	1370	C2	CuC	X	1	3.770	13.980	25.630	0.00	0.00
ATOM	1371	C2	CuC	X	1	3.770	13.980	41.930	0.00	0.00
ATOM	1372	C2	CuC	X	1	3.770	13.980	58.240	0.00	0.00
ATOM	1373	C2	CuC	X	1	3.770	13.980	74.540	0.00	0.00
ATOM	1374	C2	CuC	X	1	3.770	24.870	25.630	0.00	0.00
ATOM	1375	C2	CuC	X	1	3.770	24.870	41.930	0.00	0.00
ATOM	1376	C2	CuC	X	1	3.770	24.870	58.240	0.00	0.00
ATOM	1377	C2	CuC	X	1	3.770	24.870	74.540	0.00	0.00
ATOM	1378	C2	CuC	X	1	14.660	3.090	25.630	0.00	0.00
ATOM	1379	C2	CuC	X	1	14.660	3.090	41.930	0.00	0.00
ATOM	1380	C2	CuC	X	1	14.660	3.090	58.240	0.00	0.00
ATOM	1381	C2	CuC	X	1	14.660	3.090	74.540	0.00	0.00
ATOM	1382	C2	CuC	X	1	14.660	13.980	25.630	0.00	0.00
ATOM	1383	C2	CuC	X	1	14.660	13.980	41.930	0.00	0.00
ATOM	1384	C2	CuC	X	1	14.660	13.980	58.240	0.00	0.00

ATOM	1385	C2	CuC	X	1	14.660	13.980	74.540	0.00	0.00
ATOM	1386	C2	CuC	X	1	14.660	24.870	25.630	0.00	0.00
ATOM	1387	C2	CuC	X	1	14.660	24.870	41.930	0.00	0.00
ATOM	1388	C2	CuC	X	1	14.660	24.870	58.240	0.00	0.00
ATOM	1389	C2	CuC	X	1	14.660	24.870	74.540	0.00	0.00
ATOM	1390	C2	CuC	X	1	25.550	3.090	25.630	0.00	0.00
ATOM	1391	C2	CuC	X	1	25.550	3.090	41.930	0.00	0.00
ATOM	1392	C2	CuC	X	1	25.550	3.090	58.240	0.00	0.00
ATOM	1393	C2	CuC	X	1	25.550	3.090	74.540	0.00	0.00
ATOM	1394	C2	CuC	X	1	25.550	13.980	25.630	0.00	0.00
ATOM	1395	C2	CuC	X	1	25.550	13.980	41.930	0.00	0.00
ATOM	1396	C2	CuC	X	1	25.550	13.980	58.240	0.00	0.00
ATOM	1397	C2	CuC	X	1	25.550	13.980	74.540	0.00	0.00
ATOM	1398	C2	CuC	X	1	25.550	24.870	25.630	0.00	0.00
ATOM	1399	C2	CuC	X	1	25.550	24.870	41.930	0.00	0.00
ATOM	1400	C2	CuC	X	1	25.550	24.870	58.240	0.00	0.00
ATOM	1401	C2	CuC	X	1	25.550	24.870	74.540	0.00	0.00
ATOM	1402	C2	CuC	X	1	2.540	2.400	25.560	0.00	0.00
ATOM	1403	C2	CuC	X	1	2.540	2.400	41.860	0.00	0.00
ATOM	1404	C2	CuC	X	1	2.540	2.400	58.170	0.00	0.00
ATOM	1405	C2	CuC	X	1	2.540	2.400	74.470	0.00	0.00
ATOM	1406	C2	CuC	X	1	2.540	13.290	25.560	0.00	0.00
ATOM	1407	C2	CuC	X	1	2.540	13.290	41.860	0.00	0.00
ATOM	1408	C2	CuC	X	1	2.540	13.290	58.170	0.00	0.00

ATOM	1409	C2	CuC	X	1	2.540	13.290	74.470	0.00	0.00
ATOM	1410	C2	CuC	X	1	2.540	24.180	25.560	0.00	0.00
ATOM	1411	C2	CuC	X	1	2.540	24.180	41.860	0.00	0.00
ATOM	1412	C2	CuC	X	1	2.540	24.180	58.170	0.00	0.00
ATOM	1413	C2	CuC	X	1	2.540	24.180	74.470	0.00	0.00
ATOM	1414	C2	CuC	X	1	13.430	2.400	25.560	0.00	0.00
ATOM	1415	C2	CuC	X	1	13.430	2.400	41.860	0.00	0.00
ATOM	1416	C2	CuC	X	1	13.430	2.400	58.170	0.00	0.00
ATOM	1417	C2	CuC	X	1	13.430	2.400	74.470	0.00	0.00
ATOM	1418	C2	CuC	X	1	13.430	13.290	25.560	0.00	0.00
ATOM	1419	C2	CuC	X	1	13.430	13.290	41.860	0.00	0.00
ATOM	1420	C2	CuC	X	1	13.430	13.290	58.170	0.00	0.00
ATOM	1421	C2	CuC	X	1	13.430	13.290	74.470	0.00	0.00
ATOM	1422	C2	CuC	X	1	13.430	24.180	25.560	0.00	0.00
ATOM	1423	C2	CuC	X	1	13.430	24.180	41.860	0.00	0.00
ATOM	1424	C2	CuC	X	1	13.430	24.180	58.170	0.00	0.00
ATOM	1425	C2	CuC	X	1	13.430	24.180	74.470	0.00	0.00
ATOM	1426	C2	CuC	X	1	24.320	2.400	25.560	0.00	0.00
ATOM	1427	C2	CuC	X	1	24.320	2.400	41.860	0.00	0.00
ATOM	1428	C2	CuC	X	1	24.320	2.400	58.170	0.00	0.00
ATOM	1429	C2	CuC	X	1	24.320	2.400	74.470	0.00	0.00
ATOM	1430	C2	CuC	X	1	24.320	13.290	25.560	0.00	0.00
ATOM	1431	C2	CuC	X	1	24.320	13.290	41.860	0.00	0.00
ATOM	1432	C2	CuC	X	1	24.320	13.290	58.170	0.00	0.00

ATOM 1433 C2 CuC X 1 24.320 13.290 74.470 0.00 0.00

ATOM 1434 C2 CuC X 1 24.320 24.180 25.560 0.00 0.00

ATOM 1435 C2 CuC X 1 24.320 24.180 41.860 0.00 0.00

ATOM 1436 C2 CuC X 1 24.320 24.180 58.170 0.00 0.00

ATOM 1437 C2 CuC X 1 24.320 24.180 74.470 0.00 0.00

ATOM 1438 C2 CuC X 1 2.520 0.990 25.540 0.00 0.00

ATOM 1439 C2 CuC X 1 2.520 0.990 41.840 0.00 0.00

ATOM 1440 C2 CuC X 1 2.520 0.990 58.140 0.00 0.00

ATOM 1441 C2 CuC X 1 2.520 0.990 74.450 0.00 0.00

ATOM 1442 C2 CuC X 1 2.520 11.880 25.540 0.00 0.00

ATOM 1443 C2 CuC X 1 2.520 11.880 41.840 0.00 0.00

ATOM 1444 C2 CuC X 1 2.520 11.880 58.140 0.00 0.00

ATOM 1445 C2 CuC X 1 2.520 11.880 74.450 0.00 0.00

ATOM 1446 C2 CuC X 1 2.520 22.770 25.540 0.00 0.00

ATOM 1447 C2 CuC X 1 2.520 22.770 41.840 0.00 0.00

ATOM 1448 C2 CuC X 1 2.520 22.770 58.140 0.00 0.00

ATOM 1449 C2 CuC X 1 2.520 22.770 74.450 0.00 0.00

ATOM 1450 C2 CuC X 1 13.410 0.990 25.540 0.00 0.00

ATOM 1451 C2 CuC X 1 13.410 0.990 41.840 0.00 0.00

ATOM 1452 C2 CuC X 1 13.410 0.990 58.140 0.00 0.00

ATOM 1453 C2 CuC X 1 13.410 0.990 74.450 0.00 0.00

ATOM 1454 C2 CuC X 1 13.410 11.880 25.540 0.00 0.00

ATOM 1455 C2 CuC X 1 13.410 11.880 41.840 0.00 0.00

ATOM 1456 C2 CuC X 1 13.410 11.880 58.140 0.00 0.00

ATOM	1457	C2	CuC	X	1	13.410	11.880	74.450	0.00	0.00
ATOM	1458	C2	CuC	X	1	13.410	22.770	25.540	0.00	0.00
ATOM	1459	C2	CuC	X	1	13.410	22.770	41.840	0.00	0.00
ATOM	1460	C2	CuC	X	1	13.410	22.770	58.140	0.00	0.00
ATOM	1461	C2	CuC	X	1	13.410	22.770	74.450	0.00	0.00
ATOM	1462	C2	CuC	X	1	24.310	0.990	25.540	0.00	0.00
ATOM	1463	C2	CuC	X	1	24.310	0.990	41.840	0.00	0.00
ATOM	1464	C2	CuC	X	1	24.310	0.990	58.140	0.00	0.00
ATOM	1465	C2	CuC	X	1	24.310	0.990	74.450	0.00	0.00
ATOM	1466	C2	CuC	X	1	24.310	11.880	25.540	0.00	0.00
ATOM	1467	C2	CuC	X	1	24.310	11.880	41.840	0.00	0.00
ATOM	1468	C2	CuC	X	1	24.310	11.880	58.140	0.00	0.00
ATOM	1469	C2	CuC	X	1	24.310	11.880	74.450	0.00	0.00
ATOM	1470	C2	CuC	X	1	24.310	22.770	25.540	0.00	0.00
ATOM	1471	C2	CuC	X	1	24.310	22.770	41.840	0.00	0.00
ATOM	1472	C2	CuC	X	1	24.310	22.770	58.140	0.00	0.00
ATOM	1473	C2	CuC	X	1	24.310	22.770	74.450	0.00	0.00
ATOM	1474	C2	CuC	X	1	3.740	0.280	25.540	0.00	0.00
ATOM	1475	C2	CuC	X	1	3.740	0.280	41.840	0.00	0.00
ATOM	1476	C2	CuC	X	1	3.740	0.280	58.140	0.00	0.00
ATOM	1477	C2	CuC	X	1	3.740	0.280	74.450	0.00	0.00
ATOM	1478	C2	CuC	X	1	3.740	11.170	25.540	0.00	0.00
ATOM	1479	C2	CuC	X	1	3.740	11.170	41.840	0.00	0.00
ATOM	1480	C2	CuC	X	1	3.740	11.170	58.140	0.00	0.00

ATOM	1481	C2	CuC	X	1	3.740	11.170	74.450	0.00	0.00
ATOM	1482	C2	CuC	X	1	3.740	22.070	25.540	0.00	0.00
ATOM	1483	C2	CuC	X	1	3.740	22.070	41.840	0.00	0.00
ATOM	1484	C2	CuC	X	1	3.740	22.070	58.140	0.00	0.00
ATOM	1485	C2	CuC	X	1	3.740	22.070	74.450	0.00	0.00
ATOM	1486	C2	CuC	X	1	14.640	0.280	25.540	0.00	0.00
ATOM	1487	C2	CuC	X	1	14.640	0.280	41.840	0.00	0.00
ATOM	1488	C2	CuC	X	1	14.640	0.280	58.140	0.00	0.00
ATOM	1489	C2	CuC	X	1	14.640	0.280	74.450	0.00	0.00
ATOM	1490	C2	CuC	X	1	14.640	11.170	25.540	0.00	0.00
ATOM	1491	C2	CuC	X	1	14.640	11.170	41.840	0.00	0.00
ATOM	1492	C2	CuC	X	1	14.640	11.170	58.140	0.00	0.00
ATOM	1493	C2	CuC	X	1	14.640	11.170	74.450	0.00	0.00
ATOM	1494	C2	CuC	X	1	14.640	22.070	25.540	0.00	0.00
ATOM	1495	C2	CuC	X	1	14.640	22.070	41.840	0.00	0.00
ATOM	1496	C2	CuC	X	1	14.640	22.070	58.140	0.00	0.00
ATOM	1497	C2	CuC	X	1	14.640	22.070	74.450	0.00	0.00
ATOM	1498	C2	CuC	X	1	25.530	0.280	25.540	0.00	0.00
ATOM	1499	C2	CuC	X	1	25.530	0.280	41.840	0.00	0.00
ATOM	1500	C2	CuC	X	1	25.530	0.280	58.140	0.00	0.00
ATOM	1501	C2	CuC	X	1	25.530	0.280	74.450	0.00	0.00
ATOM	1502	C2	CuC	X	1	25.530	11.170	25.540	0.00	0.00
ATOM	1503	C2	CuC	X	1	25.530	11.170	41.840	0.00	0.00
ATOM	1504	C2	CuC	X	1	25.530	11.170	58.140	0.00	0.00

ATOM 1505 C2 CuC X 1 25.530 11.170 74.450 0.00 0.00

ATOM 1506 C2 CuC X 1 25.530 22.070 25.540 0.00 0.00

ATOM 1507 C2 CuC X 1 25.530 22.070 41.840 0.00 0.00

ATOM 1508 C2 CuC X 1 25.530 22.070 58.140 0.00 0.00

ATOM 1509 C2 CuC X 1 25.530 22.070 74.450 0.00 0.00

ATOM 1510 C2 CuC X 1 4.980 0.970 25.590 0.00 0.00

ATOM 1511 C2 CuC X 1 4.980 0.970 41.890 0.00 0.00

ATOM 1512 C2 CuC X 1 4.980 0.970 58.200 0.00 0.00

ATOM 1513 C2 CuC X 1 4.980 0.970 74.500 0.00 0.00

ATOM 1514 C2 CuC X 1 4.980 11.860 25.590 0.00 0.00

ATOM 1515 C2 CuC X 1 4.980 11.860 41.890 0.00 0.00

ATOM 1516 C2 CuC X 1 4.980 11.860 58.200 0.00 0.00

ATOM 1517 C2 CuC X 1 4.980 11.860 74.500 0.00 0.00

ATOM 1518 C2 CuC X 1 4.980 22.750 25.590 0.00 0.00

ATOM 1519 C2 CuC X 1 4.980 22.750 41.890 0.00 0.00

ATOM 1520 C2 CuC X 1 4.980 22.750 58.200 0.00 0.00

ATOM 1521 C2 CuC X 1 4.980 22.750 74.500 0.00 0.00

ATOM 1522 C2 CuC X 1 15.870 0.970 25.590 0.00 0.00

ATOM 1523 C2 CuC X 1 15.870 0.970 41.890 0.00 0.00

ATOM 1524 C2 CuC X 1 15.870 0.970 58.200 0.00 0.00

ATOM 1525 C2 CuC X 1 15.870 0.970 74.500 0.00 0.00

ATOM 1526 C2 CuC X 1 15.870 11.860 25.590 0.00 0.00

ATOM 1527 C2 CuC X 1 15.870 11.860 41.890 0.00 0.00

ATOM 1528 C2 CuC X 1 15.870 11.860 58.200 0.00 0.00

ATOM 1529 C2 CuC X 1 15.870 11.860 74.500 0.00 0.00

ATOM 1530 C2 CuC X 1 15.870 22.750 25.590 0.00 0.00

ATOM 1531 C2 CuC X 1 15.870 22.750 41.890 0.00 0.00

ATOM 1532 C2 CuC X 1 15.870 22.750 58.200 0.00 0.00

ATOM 1533 C2 CuC X 1 15.870 22.750 74.500 0.00 0.00

ATOM 1534 C2 CuC X 1 26.760 0.970 25.590 0.00 0.00

ATOM 1535 C2 CuC X 1 26.760 0.970 41.890 0.00 0.00

ATOM 1536 C2 CuC X 1 26.760 0.970 58.200 0.00 0.00

ATOM 1537 C2 CuC X 1 26.760 0.970 74.500 0.00 0.00

ATOM 1538 C2 CuC X 1 26.760 11.860 25.590 0.00 0.00

ATOM 1539 C2 CuC X 1 26.760 11.860 41.890 0.00 0.00

ATOM 1540 C2 CuC X 1 26.760 11.860 58.200 0.00 0.00

ATOM 1541 C2 CuC X 1 26.760 11.860 74.500 0.00 0.00

ATOM 1542 C2 CuC X 1 26.760 22.750 25.590 0.00 0.00

ATOM 1543 C2 CuC X 1 26.760 22.750 41.890 0.00 0.00

ATOM 1544 C2 CuC X 1 26.760 22.750 58.200 0.00 0.00

ATOM 1545 C2 CuC X 1 26.760 22.750 74.500 0.00 0.00

ATOM 1546 C2 CuC X 1 4.990 2.380 25.660 0.00 0.00

ATOM 1547 C2 CuC X 1 4.990 2.380 41.960 0.00 0.00

ATOM 1548 C2 CuC X 1 4.990 2.380 58.260 0.00 0.00

ATOM 1549 C2 CuC X 1 4.990 2.380 74.570 0.00 0.00

ATOM 1550 C2 CuC X 1 4.990 13.270 25.660 0.00 0.00

ATOM 1551 C2 CuC X 1 4.990 13.270 41.960 0.00 0.00

ATOM 1552 C2 CuC X 1 4.990 13.270 58.260 0.00 0.00

ATOM	1553	C2	CuC	X	1	4.990	13.270	74.570	0.00	0.00
ATOM	1554	C2	CuC	X	1	4.990	24.160	25.660	0.00	0.00
ATOM	1555	C2	CuC	X	1	4.990	24.160	41.960	0.00	0.00
ATOM	1556	C2	CuC	X	1	4.990	24.160	58.260	0.00	0.00
ATOM	1557	C2	CuC	X	1	4.990	24.160	74.570	0.00	0.00
ATOM	1558	C2	CuC	X	1	15.880	2.380	25.660	0.00	0.00
ATOM	1559	C2	CuC	X	1	15.880	2.380	41.960	0.00	0.00
ATOM	1560	C2	CuC	X	1	15.880	2.380	58.260	0.00	0.00
ATOM	1561	C2	CuC	X	1	15.880	2.380	74.570	0.00	0.00
ATOM	1562	C2	CuC	X	1	15.880	13.270	25.660	0.00	0.00
ATOM	1563	C2	CuC	X	1	15.880	13.270	41.960	0.00	0.00
ATOM	1564	C2	CuC	X	1	15.880	13.270	58.260	0.00	0.00
ATOM	1565	C2	CuC	X	1	15.880	13.270	74.570	0.00	0.00
ATOM	1566	C2	CuC	X	1	15.880	24.160	25.660	0.00	0.00
ATOM	1567	C2	CuC	X	1	15.880	24.160	41.960	0.00	0.00
ATOM	1568	C2	CuC	X	1	15.880	24.160	58.260	0.00	0.00
ATOM	1569	C2	CuC	X	1	15.880	24.160	74.570	0.00	0.00
ATOM	1570	C2	CuC	X	1	26.770	2.380	25.660	0.00	0.00
ATOM	1571	C2	CuC	X	1	26.770	2.380	41.960	0.00	0.00
ATOM	1572	C2	CuC	X	1	26.770	2.380	58.260	0.00	0.00
ATOM	1573	C2	CuC	X	1	26.770	2.380	74.570	0.00	0.00
ATOM	1574	C2	CuC	X	1	26.770	13.270	25.660	0.00	0.00
ATOM	1575	C2	CuC	X	1	26.770	13.270	41.960	0.00	0.00
ATOM	1576	C2	CuC	X	1	26.770	13.270	58.260	0.00	0.00

ATOM	1577	C2	CuC	X	1	26.770	13.270	74.570	0.00	0.00
ATOM	1578	C2	CuC	X	1	26.770	24.160	25.660	0.00	0.00
ATOM	1579	C2	CuC	X	1	26.770	24.160	41.960	0.00	0.00
ATOM	1580	C2	CuC	X	1	26.770	24.160	58.260	0.00	0.00
ATOM	1581	C2	CuC	X	1	26.770	24.160	74.570	0.00	0.00
ATOM	1582	H1	CuC	X	1	12.490	2.970	25.530	0.00	0.00
ATOM	1583	H1	CuC	X	1	12.490	2.970	41.840	0.00	0.00
ATOM	1584	H1	CuC	X	1	12.490	2.970	58.140	0.00	0.00
ATOM	1585	H1	CuC	X	1	12.490	2.970	74.440	0.00	0.00
ATOM	1586	H1	CuC	X	1	12.490	13.860	25.530	0.00	0.00
ATOM	1587	H1	CuC	X	1	12.490	13.860	41.840	0.00	0.00
ATOM	1588	H1	CuC	X	1	12.490	13.860	58.140	0.00	0.00
ATOM	1589	H1	CuC	X	1	12.490	13.860	74.440	0.00	0.00
ATOM	1590	H1	CuC	X	1	12.490	24.750	25.530	0.00	0.00
ATOM	1591	H1	CuC	X	1	12.490	24.750	41.840	0.00	0.00
ATOM	1592	H1	CuC	X	1	12.490	24.750	58.140	0.00	0.00
ATOM	1593	H1	CuC	X	1	12.490	24.750	74.440	0.00	0.00
ATOM	1594	H1	CuC	X	1	23.380	2.970	25.530	0.00	0.00
ATOM	1595	H1	CuC	X	1	23.380	2.970	41.840	0.00	0.00
ATOM	1596	H1	CuC	X	1	23.380	2.970	58.140	0.00	0.00
ATOM	1597	H1	CuC	X	1	23.380	2.970	74.440	0.00	0.00
ATOM	1598	H1	CuC	X	1	23.380	13.860	25.530	0.00	0.00
ATOM	1599	H1	CuC	X	1	23.380	13.860	41.840	0.00	0.00
ATOM	1600	H1	CuC	X	1	23.380	13.860	58.140	0.00	0.00

ATOM	1601	H1	CuC	X	1	23.380	13.860	74.440	0.00	0.00
ATOM	1602	H1	CuC	X	1	23.380	24.750	25.530	0.00	0.00
ATOM	1603	H1	CuC	X	1	23.380	24.750	41.840	0.00	0.00
ATOM	1604	H1	CuC	X	1	23.380	24.750	58.140	0.00	0.00
ATOM	1605	H1	CuC	X	1	23.380	24.750	74.440	0.00	0.00
ATOM	1606	H1	CuC	X	1	12.460	0.440	25.510	0.00	0.00
ATOM	1607	H1	CuC	X	1	12.460	0.440	41.810	0.00	0.00
ATOM	1608	H1	CuC	X	1	12.460	0.440	58.120	0.00	0.00
ATOM	1609	H1	CuC	X	1	12.460	0.440	74.420	0.00	0.00
ATOM	1610	H1	CuC	X	1	12.460	11.330	25.510	0.00	0.00
ATOM	1611	H1	CuC	X	1	12.460	11.330	41.810	0.00	0.00
ATOM	1612	H1	CuC	X	1	12.460	11.330	58.120	0.00	0.00
ATOM	1613	H1	CuC	X	1	12.460	11.330	74.420	0.00	0.00
ATOM	1614	H1	CuC	X	1	12.460	22.220	25.510	0.00	0.00
ATOM	1615	H1	CuC	X	1	12.460	22.220	41.810	0.00	0.00
ATOM	1616	H1	CuC	X	1	12.460	22.220	58.120	0.00	0.00
ATOM	1617	H1	CuC	X	1	12.460	22.220	74.420	0.00	0.00
ATOM	1618	H1	CuC	X	1	23.350	0.440	25.510	0.00	0.00
ATOM	1619	H1	CuC	X	1	23.350	0.440	41.810	0.00	0.00
ATOM	1620	H1	CuC	X	1	23.350	0.440	58.120	0.00	0.00
ATOM	1621	H1	CuC	X	1	23.350	0.440	74.420	0.00	0.00
ATOM	1622	H1	CuC	X	1	23.350	11.330	25.510	0.00	0.00
ATOM	1623	H1	CuC	X	1	23.350	11.330	41.810	0.00	0.00
ATOM	1624	H1	CuC	X	1	23.350	11.330	58.120	0.00	0.00

ATOM 1625 H1 CuC X 1 23.350 11.330 74.420 0.00 0.00

ATOM 1626 H1 CuC X 1 23.350 22.220 25.510 0.00 0.00

ATOM 1627 H1 CuC X 1 23.350 22.220 41.810 0.00 0.00

ATOM 1628 H1 CuC X 1 23.350 22.220 58.120 0.00 0.00

ATOM 1629 H1 CuC X 1 23.350 22.220 74.420 0.00 0.00

ATOM 1630 H1 CuC X 1 5.920 0.400 25.590 0.00 0.00

ATOM 1631 H1 CuC X 1 5.920 0.400 41.890 0.00 0.00

ATOM 1632 H1 CuC X 1 5.920 0.400 58.190 0.00 0.00

ATOM 1633 H1 CuC X 1 5.920 0.400 74.500 0.00 0.00

ATOM 1634 H1 CuC X 1 5.920 11.290 25.590 0.00 0.00

ATOM 1635 H1 CuC X 1 5.920 11.290 41.890 0.00 0.00

ATOM 1636 H1 CuC X 1 5.920 11.290 58.190 0.00 0.00

ATOM 1637 H1 CuC X 1 5.920 11.290 74.500 0.00 0.00

ATOM 1638 H1 CuC X 1 5.920 22.190 25.590 0.00 0.00

ATOM 1639 H1 CuC X 1 5.920 22.190 41.890 0.00 0.00

ATOM 1640 H1 CuC X 1 5.920 22.190 58.190 0.00 0.00

ATOM 1641 H1 CuC X 1 5.920 22.190 74.500 0.00 0.00

ATOM 1642 H1 CuC X 1 16.810 0.400 25.590 0.00 0.00

ATOM 1643 H1 CuC X 1 16.810 0.400 41.890 0.00 0.00

ATOM 1644 H1 CuC X 1 16.810 0.400 58.190 0.00 0.00

ATOM 1645 H1 CuC X 1 16.810 0.400 74.500 0.00 0.00

ATOM 1646 H1 CuC X 1 16.810 11.290 25.590 0.00 0.00

ATOM 1647 H1 CuC X 1 16.810 11.290 41.890 0.00 0.00

ATOM 1648 H1 CuC X 1 16.810 11.290 58.190 0.00 0.00

ATOM	1649	H1	CuC	X	1	16.810	11.290	74.500	0.00	0.00
ATOM	1650	H1	CuC	X	1	16.810	22.190	25.590	0.00	0.00
ATOM	1651	H1	CuC	X	1	16.810	22.190	41.890	0.00	0.00
ATOM	1652	H1	CuC	X	1	16.810	22.190	58.190	0.00	0.00
ATOM	1653	H1	CuC	X	1	16.810	22.190	74.500	0.00	0.00
ATOM	1654	H1	CuC	X	1	27.700	0.400	25.590	0.00	0.00
ATOM	1655	H1	CuC	X	1	27.700	0.400	41.890	0.00	0.00
ATOM	1656	H1	CuC	X	1	27.700	0.400	58.190	0.00	0.00
ATOM	1657	H1	CuC	X	1	27.700	0.400	74.500	0.00	0.00
ATOM	1658	H1	CuC	X	1	27.700	11.290	25.590	0.00	0.00
ATOM	1659	H1	CuC	X	1	27.700	11.290	41.890	0.00	0.00
ATOM	1660	H1	CuC	X	1	27.700	11.290	58.190	0.00	0.00
ATOM	1661	H1	CuC	X	1	27.700	11.290	74.500	0.00	0.00
ATOM	1662	H1	CuC	X	1	27.700	22.190	25.590	0.00	0.00
ATOM	1663	H1	CuC	X	1	27.700	22.190	41.890	0.00	0.00
ATOM	1664	H1	CuC	X	1	27.700	22.190	58.190	0.00	0.00
ATOM	1665	H1	CuC	X	1	27.700	22.190	74.500	0.00	0.00
ATOM	1666	H1	CuC	X	1	5.940	2.930	25.720	0.00	0.00
ATOM	1667	H1	CuC	X	1	5.940	2.930	42.020	0.00	0.00
ATOM	1668	H1	CuC	X	1	5.940	2.930	58.330	0.00	0.00
ATOM	1669	H1	CuC	X	1	5.940	2.930	74.630	0.00	0.00
ATOM	1670	H1	CuC	X	1	5.940	13.820	25.720	0.00	0.00
ATOM	1671	H1	CuC	X	1	5.940	13.820	42.020	0.00	0.00
ATOM	1672	H1	CuC	X	1	5.940	13.820	58.330	0.00	0.00

ATOM 1673 H1 CuC X 1 5.940 13.820 74.630 0.00 0.00

ATOM 1674 H1 CuC X 1 5.940 24.710 25.720 0.00 0.00

ATOM 1675 H1 CuC X 1 5.940 24.710 42.020 0.00 0.00

ATOM 1676 H1 CuC X 1 5.940 24.710 58.330 0.00 0.00

ATOM 1677 H1 CuC X 1 5.940 24.710 74.630 0.00 0.00

ATOM 1678 H1 CuC X 1 16.830 2.930 25.720 0.00 0.00

ATOM 1679 H1 CuC X 1 16.830 2.930 42.020 0.00 0.00

ATOM 1680 H1 CuC X 1 16.830 2.930 58.330 0.00 0.00

ATOM 1681 H1 CuC X 1 16.830 2.930 74.630 0.00 0.00

ATOM 1682 H1 CuC X 1 16.830 13.820 25.720 0.00 0.00

ATOM 1683 H1 CuC X 1 16.830 13.820 42.020 0.00 0.00

ATOM 1684 H1 CuC X 1 16.830 13.820 58.330 0.00 0.00

ATOM 1685 H1 CuC X 1 16.830 13.820 74.630 0.00 0.00

ATOM 1686 H1 CuC X 1 16.830 24.710 25.720 0.00 0.00

ATOM 1687 H1 CuC X 1 16.830 24.710 42.020 0.00 0.00

ATOM 1688 H1 CuC X 1 16.830 24.710 58.330 0.00 0.00

ATOM 1689 H1 CuC X 1 16.830 24.710 74.630 0.00 0.00

ATOM 1690 H1 CuC X 1 27.720 2.930 25.720 0.00 0.00

ATOM 1691 H1 CuC X 1 27.720 2.930 42.020 0.00 0.00

ATOM 1692 H1 CuC X 1 27.720 2.930 58.330 0.00 0.00

ATOM 1693 H1 CuC X 1 27.720 2.930 74.630 0.00 0.00

ATOM 1694 H1 CuC X 1 27.720 13.820 25.720 0.00 0.00

ATOM 1695 H1 CuC X 1 27.720 13.820 42.020 0.00 0.00

ATOM 1696 H1 CuC X 1 27.720 13.820 58.330 0.00 0.00

ATOM 1697 H1 CuC X 1 27.720 13.820 74.630 0.00 0.00

ATOM 1698 H1 CuC X 1 27.720 24.710 25.720 0.00 0.00

ATOM 1699 H1 CuC X 1 27.720 24.710 42.020 0.00 0.00

ATOM 1700 H1 CuC X 1 27.720 24.710 58.330 0.00 0.00

ATOM 1701 H1 CuC X 1 27.720 24.710 74.630 0.00 0.00

ATOM 1702 C1 CuC X 1 3.770 4.590 25.640 0.00 0.00

ATOM 1703 C1 CuC X 1 3.770 4.590 41.940 0.00 0.00

ATOM 1704 C1 CuC X 1 3.770 4.590 58.240 0.00 0.00

ATOM 1705 C1 CuC X 1 3.770 4.590 74.550 0.00 0.00

ATOM 1706 C1 CuC X 1 3.770 15.490 25.640 0.00 0.00

ATOM 1707 C1 CuC X 1 3.770 15.490 41.940 0.00 0.00

ATOM 1708 C1 CuC X 1 3.770 15.490 58.240 0.00 0.00

ATOM 1709 C1 CuC X 1 3.770 15.490 74.550 0.00 0.00

ATOM 1710 C1 CuC X 1 3.770 26.380 25.640 0.00 0.00

ATOM 1711 C1 CuC X 1 3.770 26.380 41.940 0.00 0.00

ATOM 1712 C1 CuC X 1 3.770 26.380 58.240 0.00 0.00

ATOM 1713 C1 CuC X 1 3.770 26.380 74.550 0.00 0.00

ATOM 1714 C1 CuC X 1 14.660 4.590 25.640 0.00 0.00

ATOM 1715 C1 CuC X 1 14.660 4.590 41.940 0.00 0.00

ATOM 1716 C1 CuC X 1 14.660 4.590 58.240 0.00 0.00

ATOM 1717 C1 CuC X 1 14.660 4.590 74.550 0.00 0.00

ATOM 1718 C1 CuC X 1 14.660 15.490 25.640 0.00 0.00

ATOM 1719 C1 CuC X 1 14.660 15.490 41.940 0.00 0.00

ATOM 1720 C1 CuC X 1 14.660 15.490 58.240 0.00 0.00

ATOM 1721 C1 CuC X 1 14.660 15.490 74.550 0.00 0.00

ATOM 1722 C1 CuC X 1 14.660 26.380 25.640 0.00 0.00

ATOM 1723 C1 CuC X 1 14.660 26.380 41.940 0.00 0.00

ATOM 1724 C1 CuC X 1 14.660 26.380 58.240 0.00 0.00

ATOM 1725 C1 CuC X 1 14.660 26.380 74.550 0.00 0.00

ATOM 1726 C1 CuC X 1 25.550 4.590 25.640 0.00 0.00

ATOM 1727 C1 CuC X 1 25.550 4.590 41.940 0.00 0.00

ATOM 1728 C1 CuC X 1 25.550 4.590 58.240 0.00 0.00

ATOM 1729 C1 CuC X 1 25.550 4.590 74.550 0.00 0.00

ATOM 1730 C1 CuC X 1 25.550 15.490 25.640 0.00 0.00

ATOM 1731 C1 CuC X 1 25.550 15.490 41.940 0.00 0.00

ATOM 1732 C1 CuC X 1 25.550 15.490 58.240 0.00 0.00

ATOM 1733 C1 CuC X 1 25.550 15.490 74.550 0.00 0.00

ATOM 1734 C1 CuC X 1 25.550 26.380 25.640 0.00 0.00

ATOM 1735 C1 CuC X 1 25.550 26.380 41.940 0.00 0.00

ATOM 1736 C1 CuC X 1 25.550 26.380 58.240 0.00 0.00

ATOM 1737 C1 CuC X 1 25.550 26.380 74.550 0.00 0.00

ATOM 1738 H3 CuC X 1 4.310 5.650 33.800 0.00 0.00

ATOM 1739 H3 CuC X 1 4.310 5.650 50.110 0.00 0.00

ATOM 1740 H3 CuC X 1 4.310 5.650 66.410 0.00 0.00

ATOM 1741 H3 CuC X 1 4.310 16.540 33.800 0.00 0.00

ATOM 1742 H3 CuC X 1 4.310 16.540 50.110 0.00 0.00

ATOM 1743 H3 CuC X 1 4.310 16.540 66.410 0.00 0.00

ATOM 1744 H3 CuC X 1 4.310 27.430 33.800 0.00 0.00

ATOM	1745	H3	CuC	X	1	4.310	27.430	50.110	0.00	0.00
ATOM	1746	H3	CuC	X	1	4.310	27.430	66.410	0.00	0.00
ATOM	1747	H3	CuC	X	1	15.200	5.650	33.800	0.00	0.00
ATOM	1748	H3	CuC	X	1	15.200	5.650	50.110	0.00	0.00
ATOM	1749	H3	CuC	X	1	15.200	5.650	66.410	0.00	0.00
ATOM	1750	H3	CuC	X	1	15.200	16.540	33.800	0.00	0.00
ATOM	1751	H3	CuC	X	1	15.200	16.540	50.110	0.00	0.00
ATOM	1752	H3	CuC	X	1	15.200	16.540	66.410	0.00	0.00
ATOM	1753	H3	CuC	X	1	15.200	27.430	33.800	0.00	0.00
ATOM	1754	H3	CuC	X	1	15.200	27.430	50.110	0.00	0.00
ATOM	1755	H3	CuC	X	1	15.200	27.430	66.410	0.00	0.00
ATOM	1756	H3	CuC	X	1	26.090	5.650	33.800	0.00	0.00
ATOM	1757	H3	CuC	X	1	26.090	5.650	50.110	0.00	0.00
ATOM	1758	H3	CuC	X	1	26.090	5.650	66.410	0.00	0.00
ATOM	1759	H3	CuC	X	1	26.090	16.540	33.800	0.00	0.00
ATOM	1760	H3	CuC	X	1	26.090	16.540	50.110	0.00	0.00
ATOM	1761	H3	CuC	X	1	26.090	16.540	66.410	0.00	0.00
ATOM	1762	H3	CuC	X	1	26.090	27.430	33.800	0.00	0.00
ATOM	1763	H3	CuC	X	1	26.090	27.430	50.110	0.00	0.00
ATOM	1764	H3	CuC	X	1	26.090	27.430	66.410	0.00	0.00
ATOM	1765	H3	CuC	X	1	3.440	8.630	33.610	0.00	0.00
ATOM	1766	H3	CuC	X	1	3.440	8.630	49.910	0.00	0.00
ATOM	1767	H3	CuC	X	1	3.440	8.630	66.210	0.00	0.00
ATOM	1768	H3	CuC	X	1	3.440	19.520	33.610	0.00	0.00

ATOM 1769 H3 CuC X 1 3.440 19.520 49.910 0.00 0.00

ATOM 1770 H3 CuC X 1 3.440 19.520 66.210 0.00 0.00

ATOM 1771 H3 CuC X 1 3.440 30.410 33.610 0.00 0.00

ATOM 1772 H3 CuC X 1 3.440 30.410 49.910 0.00 0.00

ATOM 1773 H3 CuC X 1 3.440 30.410 66.210 0.00 0.00

ATOM 1774 H3 CuC X 1 14.330 8.630 33.610 0.00 0.00

ATOM 1775 H3 CuC X 1 14.330 8.630 49.910 0.00 0.00

ATOM 1776 H3 CuC X 1 14.330 8.630 66.210 0.00 0.00

ATOM 1777 H3 CuC X 1 14.330 19.520 33.610 0.00 0.00

ATOM 1778 H3 CuC X 1 14.330 19.520 49.910 0.00 0.00

ATOM 1779 H3 CuC X 1 14.330 19.520 66.210 0.00 0.00

ATOM 1780 H3 CuC X 1 14.330 30.410 33.610 0.00 0.00

ATOM 1781 H3 CuC X 1 14.330 30.410 49.910 0.00 0.00

ATOM 1782 H3 CuC X 1 14.330 30.410 66.210 0.00 0.00

ATOM 1783 H3 CuC X 1 25.220 8.630 33.610 0.00 0.00

ATOM 1784 H3 CuC X 1 25.220 8.630 49.910 0.00 0.00

ATOM 1785 H3 CuC X 1 25.220 8.630 66.210 0.00 0.00

ATOM 1786 H3 CuC X 1 25.220 19.520 33.610 0.00 0.00

ATOM 1787 H3 CuC X 1 25.220 19.520 49.910 0.00 0.00

ATOM 1788 H3 CuC X 1 25.220 19.520 66.210 0.00 0.00

ATOM 1789 H3 CuC X 1 25.220 30.410 33.610 0.00 0.00

ATOM 1790 H3 CuC X 1 25.220 30.410 49.910 0.00 0.00

ATOM 1791 H3 CuC X 1 25.220 30.410 66.210 0.00 0.00

ATOM 1792 O CuC X 1 1.790 7.450 24.400 0.00 0.00

ATOM	1793	O	CuC	X	1	1.770	6.780	26.600	0.00	0.00
ATOM	1794	O	CuC	X	1	1.790	7.450	40.710	0.00	0.00
ATOM	1795	O	CuC	X	1	1.770	6.780	42.900	0.00	0.00
ATOM	1796	O	CuC	X	1	1.790	7.450	57.010	0.00	0.00
ATOM	1797	O	CuC	X	1	1.770	6.780	59.200	0.00	0.00
ATOM	1798	O	CuC	X	1	1.790	7.450	73.310	0.00	0.00
ATOM	1799	O	CuC	X	1	1.770	6.780	75.510	0.00	0.00
ATOM	1800	O	CuC	X	1	1.790	18.340	24.400	0.00	0.00
ATOM	1801	O	CuC	X	1	1.770	17.670	26.600	0.00	0.00
ATOM	1802	O	CuC	X	1	1.790	18.340	40.710	0.00	0.00
ATOM	1803	O	CuC	X	1	1.770	17.670	42.900	0.00	0.00
ATOM	1804	O	CuC	X	1	1.790	18.340	57.010	0.00	0.00
ATOM	1805	O	CuC	X	1	1.770	17.670	59.200	0.00	0.00
ATOM	1806	O	CuC	X	1	1.790	18.340	73.310	0.00	0.00
ATOM	1807	O	CuC	X	1	1.770	17.670	75.510	0.00	0.00
ATOM	1808	O	CuC	X	1	1.790	29.230	24.400	0.00	0.00
ATOM	1809	O	CuC	X	1	1.770	28.560	26.600	0.00	0.00
ATOM	1810	O	CuC	X	1	1.790	29.230	40.710	0.00	0.00
ATOM	1811	O	CuC	X	1	1.770	28.560	42.900	0.00	0.00
ATOM	1812	O	CuC	X	1	1.790	29.230	57.010	0.00	0.00
ATOM	1813	O	CuC	X	1	1.770	28.560	59.200	0.00	0.00
ATOM	1814	O	CuC	X	1	1.790	29.230	73.310	0.00	0.00
ATOM	1815	O	CuC	X	1	1.770	28.560	75.510	0.00	0.00
ATOM	1816	H3	CuC	X	1	3.300	31.370	31.430	0.00	0.00

ATOM 1817 H3 CuC X 1 3.300 31.370 47.740 0.00 0.00

ATOM 1818 H3 CuC X 1 3.300 31.370 64.040 0.00 0.00

ATOM 1819 H3 CuC X 1 14.190 31.370 31.430 0.00 0.00

ATOM 1820 H3 CuC X 1 14.190 31.370 47.740 0.00 0.00

ATOM 1821 H3 CuC X 1 14.190 31.370 64.040 0.00 0.00

ATOM 1822 H3 CuC X 1 25.080 31.370 31.430 0.00 0.00

ATOM 1823 H3 CuC X 1 25.080 31.370 47.740 0.00 0.00

ATOM 1824 H3 CuC X 1 25.080 31.370 64.040 0.00 0.00

ATOM 1825 O CuC X 1 4.040 30.890 24.400 0.00 0.00

ATOM 1826 O CuC X 1 3.400 30.880 26.600 0.00 0.00

ATOM 1827 O CuC X 1 4.040 30.890 40.710 0.00 0.00

ATOM 1828 O CuC X 1 3.400 30.880 42.910 0.00 0.00

ATOM 1829 O CuC X 1 4.040 30.890 57.010 0.00 0.00

ATOM 1830 O CuC X 1 3.400 30.880 59.210 0.00 0.00

ATOM 1831 O CuC X 1 4.040 30.890 73.310 0.00 0.00

ATOM 1832 O CuC X 1 3.400 30.880 75.510 0.00 0.00

ATOM 1833 O CuC X 1 14.940 30.890 24.400 0.00 0.00

ATOM 1834 O CuC X 1 14.290 30.880 26.600 0.00 0.00

ATOM 1835 O CuC X 1 14.940 30.890 40.710 0.00 0.00

ATOM 1836 O CuC X 1 14.290 30.880 42.910 0.00 0.00

ATOM 1837 O CuC X 1 14.940 30.890 57.010 0.00 0.00

ATOM 1838 O CuC X 1 14.290 30.880 59.210 0.00 0.00

ATOM 1839 O CuC X 1 14.940 30.890 73.310 0.00 0.00

ATOM 1840 O CuC X 1 14.290 30.880 75.510 0.00 0.00

ATOM	1841	O	CuC	X	1	25.830	30.890	24.400	0.00	0.00
ATOM	1842	O	CuC	X	1	25.180	30.880	26.600	0.00	0.00
ATOM	1843	O	CuC	X	1	25.830	30.890	40.710	0.00	0.00
ATOM	1844	O	CuC	X	1	25.180	30.880	42.910	0.00	0.00
ATOM	1845	O	CuC	X	1	25.830	30.890	57.010	0.00	0.00
ATOM	1846	O	CuC	X	1	25.180	30.880	59.210	0.00	0.00
ATOM	1847	O	CuC	X	1	25.830	30.890	73.310	0.00	0.00
ATOM	1848	O	CuC	X	1	25.180	30.880	75.510	0.00	0.00
ATOM	1849	H1	CuC	X	1	1.590	2.970	25.530	0.00	0.00
ATOM	1850	H1	CuC	X	1	1.590	2.970	41.840	0.00	0.00
ATOM	1851	H1	CuC	X	1	1.590	2.970	58.140	0.00	0.00
ATOM	1852	H1	CuC	X	1	1.590	2.970	74.440	0.00	0.00
ATOM	1853	H1	CuC	X	1	1.590	13.860	25.530	0.00	0.00
ATOM	1854	H1	CuC	X	1	1.590	13.860	41.840	0.00	0.00
ATOM	1855	H1	CuC	X	1	1.590	13.860	58.140	0.00	0.00
ATOM	1856	H1	CuC	X	1	1.590	13.860	74.440	0.00	0.00
ATOM	1857	H1	CuC	X	1	1.590	24.750	25.530	0.00	0.00
ATOM	1858	H1	CuC	X	1	1.590	24.750	41.840	0.00	0.00
ATOM	1859	H1	CuC	X	1	1.590	24.750	58.140	0.00	0.00
ATOM	1860	H1	CuC	X	1	1.590	24.750	74.440	0.00	0.00
ATOM	1861	H1	CuC	X	1	1.570	0.440	25.510	0.00	0.00
ATOM	1862	H1	CuC	X	1	1.570	0.440	41.810	0.00	0.00
ATOM	1863	H1	CuC	X	1	1.570	0.440	58.120	0.00	0.00
ATOM	1864	H1	CuC	X	1	1.570	0.440	74.420	0.00	0.00

ATOM 1865 H1 CuC X 1 1.570 11.330 25.510 0.00 0.00

ATOM 1866 H1 CuC X 1 1.570 11.330 41.810 0.00 0.00

ATOM 1867 H1 CuC X 1 1.570 11.330 58.120 0.00 0.00

ATOM 1868 H1 CuC X 1 1.570 11.330 74.420 0.00 0.00

ATOM 1869 H1 CuC X 1 1.570 22.220 25.510 0.00 0.00

ATOM 1870 H1 CuC X 1 1.570 22.220 41.810 0.00 0.00

ATOM 1871 H1 CuC X 1 1.570 22.220 58.120 0.00 0.00

ATOM 1872 H1 CuC X 1 1.570 22.220 74.420 0.00 0.00

ATOM 1873 CB MEOHX 2 19.580 14.190 40.420 0.00 0.00

ATOM 1874 OG MEOHX 2 19.630 13.400 41.630 0.00 0.00

ATOM 1875 HG1 MEOHX 2 19.250 13.860 42.380 0.00 0.00

ATOM 1876 HB1 MEOHX 2 20.080 13.700 39.560 0.00 0.00

ATOM 1877 HB2 MEOHX 2 20.110 15.150 40.570 0.00 0.00

ATOM 1878 HB3 MEOHX 2 18.560 14.370 40.040 0.00 0.00

END
